# Supplementary material for: Community ageing research 75+ study (CARE75+): an experimental ageing and frailty research cohort
Source: BMJ Open. 2019 Mar 7;9(3):e026744. doi: 10.1136/bmjopen-2018-026744 (PMC6429944; doi:10.1136/bmjopen-2018-026744)
Supplement: Supplementary file 1 [file bmjopen-2018-026744supp001.pdf]

1. Data items are grouped in tabs

2. Please indicate which items you require using **CUT AND PASTE (plain text) TO POPULATE** the SampleRequestForm.

3. EVERY item must have a variable name to be extracted. **DO NOT** only state 'all'

3. Additions / ammendments to data requests will require the submission of a new SampleRequestForm with the additions/ammendments highlighted

NB:

Any variable with an associated ‘valid’ column has been checked for an acceptable level of missing data. Valid=1.

Missing data is recorded as follows:

Not applicable will be recorded in the dataset as -95

Unable will be recorded in the dataset as -96

Unwilling will be recorded in the dataset as -97

Unavailable information will be recorded in the dataset as -98

All other missing blank fields will be -99

Required Field (please X)	Variable	Variable Label	Values	Value Label	comment
	participant_id	unique participant id for linkage			
	nhs_number				
	registration_date	date of upload to database			
	surname				
	forenames				
	title				
	address_l1				
	address_l2				
	address_l3				
	address_l4				
	address_l5				
	postcode				
	home_telephone				
	work_telephone				
	mobile_telephone				
	SixMonthFollowUpPreference	how the participant wc	1	face to face	
			2	on-line	
			3	telephone	
			9	no preference	
	EmailAddress		verabtim		

Required Field (please X)	Variable	Variable Label	Values	Value Labels	comment		
	participantid	unique identification number	100000+				
	Site	unique site number	Null	Bradford Leeds			
	NHSNumber	NHS assigned number	verbatim				
	surname	participant surname	verbatim				
	forenames	participant forenames	verbatim				
	gp_code	participant's general practice	verbatim				
	status	study status of participant	0	Not contacted			
			1	Attempt contact again			
			2	Consent visit			
			99	OOS			
			100	Baseline stage, Visit {0}			
			101	Baseline stage, COMPLETE			
			200	6 month stage, Visit {0}			
			201	6 month stage, COMPLETE			
			300	12 month stage, Visit {0}			
			301	12 month stage, COMPLETE			
			400	24 month stage, Visit {0}			
			401	24 month stage, COMPLETE			
			500	48 month stage, Visit {0}			
			501	48 month stage, COMPLETE			
			600	Post 48 month stage, Visit {0}			
			601	Post 48 month stage, COMPLETE			
			999	OOS			
	StartOfStageDate	date and time that data is changed entered in nev	yyyy-mm-dd / hh/mm/ss				
	dob	participant's date of birth	yyyy/mm/dd				
	date_of_death	participant's date of death	yyyy/mm/dd				
	sex	participant's gender	1	male			
			2	female			
	ethnicity	participant's ethnic origin	1	white			
			2	mixed white /black caribbean			
			3	mixed white / asian			
			4	mixed white / black african			
			5	other mixed			
			6	black african			
			7	asian indian			
			8	asian bangladeshi			
			9	asian pakistani			
			10	other asian			
			11	black caribbean			
			12	other black			
			13	chinese			
			14	other			
	Otherethnicity		verbatim				
	MaritalStatus	participant's martial status	1	Single, that is never married			
			2	Married			
			3	Remarried			
			4	Separated but still legally married			
			5	Divorced			
			6	Widowed			
			97/98/99	missing			
	FutureStudies_ParticipantConsent	interest in taking part in future studies	1	Yes			
			2	No			
			97/98/99	missing			
			2	no			
			97/98/99	missing			
	otherStudies	are you currently participating in any other resear	1	yes			
			2	no			
			97/98/99	missing			
	otherStudiesYes	details of other studies	verbatim				

Required Field (please X)	Variable	Variable Label	Values	Value Label	comment
	HouseType	housing type	1	Bungalow	
			2	Semi-detached house	
			3	Detached house	
			4	Terraced house	
			5	Flat (which floor)	
			6	Sheltered housing (which floor)	
			7	Extra care housing	
			8	Care home (residential)	
			9	Care home (nursing)	
			97/98/99	Missing	
	Temp	temperature in c of the ro	verbatim		
	OtherRoomTemp	are all your rooms a simila	1	yes	
			2	no	
			97/98/99	missing	
	WhyNotRoomsSimilarTemp	reason for temperature di	verbatim		
	LivingCircumstances	participant's living circum	1	lives alone	
			2	lives with partner/spouse	
			3	lives with family	
			4	lives with a friend	
			5	lives with other residents of a care home	
			97/98/99	missing	
	OwnHome	Participant/husband/wife	1	Yes	
			2	No	
			97/98/99	missing	
	HowLongAtCurrentAddress	How long lived at current	Verbatim		
	GoOutAloneInDay	Go out alone in day	1	Yes	
			2	No	
			97/98/99	missing	
	GoOutAloneInNight	Go out alone in night	1	Yes	
			2	No	
			97/98/99	missing	
	HowSafeDoYouFeelDuringDay	How safe (would) feel wal	1	Very safe	NB this area=within 15 min walk
			2	Fairly safe	
			3	A bit unsafe	
			4	Very unsafe	
			97/98/99	missing	
	HowSafeDoYouFeelDuringNight	How safe (would) feel wal	1	Very safe	NB this area=within 15 min walk
			2	Fairly safe	
			3	A bit unsafe	
			4	Very unsafe	
			97/98/99	missing	
Yes value as follows as adjacent question					
1	ConcernsGettingOutNone	What concerns, if any, stop you getting out & about			
2	ConcernsGettingOutTraffic				
3	ConcernsGettingOutAntiSocialBeha		97/98/99	missing	
4	ConcernsGettingOutFalling				
5	ConcernsGettingOutCrime				
6	ConcernsGettingOutOther				
	ConcernsGettingOutOtherDetails		Verbatim		
	HowSafeDoYouFeelCrossingRoads	How safe feel crossing roa	1	Very safe	
			2	Fairly safe	
			3	A bit unsafe	
			4	Very unsafe	
			97/98/99	missing	
	HowWorriedAboutBeingVictimOfC	How worried about being	1	Very	
			2	Fairly	
			3	Not very	
			4	Not at all	
			97/98/99		

Required Field (please X)	Variable	Variable Label	Values	Value Label	comment
	ageEducation	how old were you when you finished full time continuous education '	number		
	PaidWork	what was the last paid work that you did?'	number		
	Qualifications	what was the highest educational qualification you attained?'	1	GCSE	
			2	HNS/HND	
			3	diploma	
			4	AS and A level	
			5	bachelor's degree	
			6	postgraduate	
			7	no qualifications	
			97/98/99	missing	
	VoluntaryWork	do you currently do any voluntary work?'	1	yes	
			2	no	
			97/98/99	missing	

Required Field (please X)	Variable	Variable Label	Values	Value Label	comment
	HaveBrothers	did/do you have any...	number		
	HaveSisters	did/do you have any...	97/98/99	missing	
	HaveSons	did/do you have any...			
	HaveDaughters	did/do you have any...			
	HaveGrandsons	did/do you have any...			
	HaveGranddaughters	did/do you have any...			
	BrotherFirstName	did/do you have any...	verbatim		
	SisterFirstName	did/do you have any...			
	SonFirstName	did/do you have any...			
	DaughterFirstName	did/do you have any...			
	GrandsonFirstName	did/do you have any...			
	GraddaughterFirstName	did/do you have any...			
	BrotherAliveorDead	did/do you have any...	1	alive	
	SisterAliveorDead	did/do you have any...	2	dead	
	SonAliveorDead	did/do you have any...	97/98/99	missing	
	DaughterAliveorDead	did/do you have any...			
	GrandsonAliveorDead	did/do you have any...			
	GranddaughterAliveorDead	did/do you have any...			

Required Field (please X)	Variable	Variable Label	Values	Value Label	comment		
		InformalSupportLift	If you needed a lift to somewhere urgently, could you ask someone for help?'	1	Yes		
		InformalSupportIll	If you wer ill in bed and needed help at home, could you ask someone for help?'	2	No		
				97/98/99	Missing		
		informal SupportCrisisNumberof PeopleforSupport	If you had a serious personal crisis, how many people could you turn to for comfort and support?'	number			
				97/98/99	Missing		
		informalSupporPeopleLiveClose	How many of these people live within a 15-20 minute drive, if any?'	number			
				97/98/99	Missing		
		InformalSupportBrother	Can you tellme the people to whom you could turn to for help (you can choose more than one)?'	1	yes		
		InformalSupportSister		2	no		
		InformalSupportDaughter		97/98/99	missing		
		InformalSupportSon					
		InformalSupportGranddaughter					
		InformalSupportGrandson					
		InformalSupportFriend					
		InformalSupportNeighbour					
		InformalSupportOther		verbatim			
		informalSupportLastFourWeeks	approximately how many hours of informal suport have you had in the last four weeks? (unpaid)	number			
				97/98/99	Missing		
		ServicesVisitWarden_ServicesVisitsFrequency	In the last 4 weeks, have you seen or had a visit from, or to, any of the following services? If so, how often?'	1	Several times a day		
		ServicesVisitHomecare_ServicesVisitsFrequency		2	Once a day		
		ServicesVisitNightattendentsitter_ServicesVisitsFrequency		3	One or more times a week		
		ServicesVisitDaysitter_ServicesVisitsFrequency		4	Less than once a week		
		ServicesVisitMealsProvision_ServicesVisitsFrequency		5	No contact		
		ServicesVisitCommunityNurse_ServicesVisitsFrequency		6	Don't know		
		ServicesVisitPhysiotherapist_ServicesVisitsFrequency		7	Not applicable		
		ServicesVisitOccupationalTherapist_ServicesVisitsFrequency		97/98/99	Missing		
		ServicesVisitChiropodist_ServicesVisitsFrequency					
		ServicesVisitSpeachTherapist_ServicesVisitsFrequency					
		ServicesVisitDietician_ServicesVisitsFrequency					
		ServicesVisitSocialWorker_ServicesVisitsFrequency					
		FormalSupportLastFourWeeks	Approximately how many hours of formal support have you had in the last four weeks?'	number			
				97/98/99	Missing		
		GPVisit	In the last 4 weeks have you been to see or had a visit from a GP?'	1	Yes		
		OutpatientClinic	In the last 4 weeks have you attended an outpatients clinic?'	2	No		
				97/98/99	Missing		
		GPVisitHowOften	number of visits	number			
		OutpatientClinicHowOften	number of visits	number			
				97/98/99	Missing		
		AidAtHomeGrabRails	Do you use any of the following adi/adaptations to help you at home (>1)	1	yes		
		AidAtHomeToiletSeat		2	yes		
		AidAtHomeBathSeat		3	yes		
		AidAtHomeReclinerChair		4	yes		
		AidAtHomeBedRail		5	yes		
		AidAtHomeBedRisers		6	yes		
		AidAtHomeHoist		7	yes		
		AidAtHomeHelpingHand		8	yes		
		AidAtHomeKeySafe		9	yes		
		AidAtHomePendantAlarm		10	yes		
		AidAtHomeStairLift		11	yes		
		AidAtHomeOutsideStep		12	yes		
		AidAtHomeWheelchair		13	yes		
				0	no		
				97/98/99	Missing		

Required Field (please X)	Variable	Variable Label	Values	Value Label comment
	Smoker	Have you ever smoked (this can include cigaretters, roll-ups, piopes or cigars)?		
			1	Yes
			2	No
			97/98/99	Missing
	SmokeRecently	Do you smoke at all nowadays'?		
			1	Yes
			2	No
			97/98/99	Missing
	HowManyCigarettes	How many cigarettes as day do y	number	
			97/98/99	Missing
	HowMnayRollUpsOrPipes	If you smoke, or have smoked, ro	number	
			97/98/99	Missing
	HowManyYears	Approximately how mnay years h	number	
			97/98/99	Missing
	CALC_CigarettePackYears	Lifetime exposure		
			number	
			97/98/99	Missing
	CALC_TobaccoPackYears	Lifetime exposure		
			number	
			97/98/99	Missing
	drinkAlcohol	Do you ever drink alcohol incuding drinks you brew at home?'		
			1	Yes
			2	No
			97/98/99	Missing
	AlcoholLast12Months	...how often have you had na alcoholoic drink of any kind during the last 12 months?'		
			1	almost every day
			2	five or 6 days a week
			3	three or four days a week
			4	once or twice a week
			5	once or twice a month
			6	once every couple of months
			7	once or twice a year
			8	not at all in the last 12 months
			9	unsure
			97/98/99	Missing
	AlcoholLast7Days	how many days out of the last 7	number	
			97/98/99	Missing
	smallGlassOfWine	Thinking about the last 7 days, h	number	
	standardGlassOfWine		97/98/99	Missing
	LargeGlassOfWine			
	lowerStrengthBeer			
	botlleOfBeer			
	canOfBeer			
	Alcopop			
	SingleSpirit			

Required Field (please X)	Variable	Variable Label	Values	Value Label	comment
	HearingAid	Do you have a hearing aid?'			
			1	Yes	
			2	No	
			3	Don't know	
			97/98/99	Missing	
	HearingAidUsage	How often do you use a hearing aid?'			
			1	Always	
			2	Regularly	
			3	Only on special occasions	
			4	Never	
			97/98/99	Missing	
	HearingTest	Have you had a hearing test in the last year?'			
			1	Yes	
			2	No	
			3	Don't know	
			97/98/99	Missing	
	DifficultyHearing	Interviewer's assessment of participant's difficulty hearing so far			
			1	No difficulty	
			2	Some difficulty	
			3	Unable to hear at all	
			97/98/99	Missing	
	HearingInQuietRoom	Do you have difficultly hearing someo	1	Yes	
			2	No	
			3	Don't know	
			97/98/99	Missing	
	HearingInLoudRoom	Do you find it difficult to follow a conv	1	Yes	
			2	No	
			3	Don't know	
			97/98/99	Missing	
	WhisperTestResult	Whisper hearing test result	1	Pass	
			2	Fail	
			97/98/99	Missing	
	GlassesOrLenses	Do you use glasses/contact lenses?'	1	Yes	
			2	No	
			3	Don't know	
			97/98/99	Missing	
	GlassesOrLensesUsedFor	purpose of glasses/lenses	1	Distance	
			2	Reading	
			3	Both distance & reading	
			4	Don't know	
			97/98/99	Missing	
	EyesightTested	...eyesight tested by an optician in the	1	Yes	
			2	No	
			3	Don't know	
			97/98/99	Missing	
	RecognisingPeople	....difficulty recognisign a friend across	1	Yes	
			2	No	
			3	Don't know	
			97/98/99	Missing	
	ReadingNewsprint	...difficulty reading ordinary newsprint	1	Yes	
			2	No	
			3	Don't know	
			97/98/99	Missing	
	BlindorPartiallySighted	Registered blind or partially sighted	1	Yes	
			2	No	
			3	Don't know	
			97/98/99	Missing	
	LeftEye	(no pinholes) is the participant able to	1	Yes	
			2	No- form vision only	
			3	No- no vision	
			97/98/99	Missing	
	LeftEyeYesMarginLabel	logMAR margin of the last line read co	number		
			97/98/99	Missing	
	LeftEyeYesNumLettersMissed	total number of letters missed or inco	number		
			97/98/99		
	RightEye	(no pinholes) is the participant able to	1	Yes	
			2	No- form vision only	
			3	No- no vision	
			97/98/99	Missing	
	RightEyeYesMarginLabel	logMAR margin of the last line read co	number		
			97/98/99	Missing	
	RightEyeYesNumLettersMissed	total number of letters missed or inco	number		
			97/98/99		
	LeftEyePinholes	(with pinholes) is the participant able	1	Yes	
			2	No- form vision only	
			3	No- no vision	
			97/98/99	Missing	
	LeftEyePinholesYesMarginLabel	logMAR margin of the last line read co	number		
			97/98/99	Missing	
	LeftEyePinholesYesNumLettersMissed	total number of letters missed or inco	number		
			97/98/99		
	RightEyePinholes	(with pinholes) is the participant able	1	Yes	
			2	No- form vision only	
			3	No- no vision	
			97/98/99	Missing	
	RightEyePinholesYesMarginLabel	logMAR margin of the last line read co	number		
			97/98/99	Missing	
	RightEyePinholesYesNumLettersMissed	total number of letters missed or inco	number		
			97/98/99		
	CALC_LogMARLeftEye	no pinhole score	number		
			97/98/99		
	CALC_LogMARRightEye	no pinhole score	number		
			97/98/99		
	CALC_LogMARLeftEyePinholes	with pinholes score	number		
			97/98/99		
	CALC_LogMARRightEyePinholes	with pinholes	number		
			97/98/99		

Required Field (please X)	Variable	Variable Label	Values	Value Label	comment		
	SF-36HealthSurvey	In general how would you say your health is?'	1	Excellent			
			2	Very Good			
			3	Good			
			4	Fair			
			5	Poor			
			97/98/99	Missing			
	healthComparedToAYearAgo	Compared to one year ago, how would your rate your health in general now?	1	Much better now than one year ago			
			2	Somewhat better now than one year ago			
			3	About the same as one year ago			
			4	Somewhat worse now than one year ago			
			5	Much worse now than one year ago			
			97/98/99	Missing			
	VigorousActivities_SF36Activities	...does you health now limit you in these activities? If so how much?'	1	Limited a lot			
	ModerateActivities_SF36Activities		2	Limited a little			
	LiftingOrCarryingActivities_SF36Activities		3	Not limited at all			
	ClimbingSeveralStairsActivities_SF36Activities		97/98/99	Missing			
	ClimbingStairsActivities_SF36Activities						
	BendingKneelingStoopingActivities_SF36Activities						
	WalkingMoreThanAMile_SF36Activities						
	WalkingSeveralBlocksActivities_SF36Activities						
	WalkingOneBlockActivities_SF36Activities						
	BathingOrDressingYourself_SF36Activities						
	HealthIssuesReducedTimeSpentOnWork	During the past 4 weeks, have you had any of the following problems with your work	1	Yes			
	HealthIssuesAccomplishedLess		2	No			
	HealthIssuesLimitedOtherActivities		97/98/99	Missing			
	HealthIssuesDifficultiesPerformingWork						
	EmotionalIssuesReducedTimeSpentOnWork	During the past 4 weeks, have you had any of the following problems with your work	1	Yes			
	EmotionalIssuesAccomplishedLess		2	No			
	EmotionalIssuesDidntDoWorkAsCarefully		97/98/99	Missing			
	HealthInterferedActivities	During the past 4 weeks, to what extent has your physical health or emotional proble	1	Not at all			
			2	Slightly			
			3	Moderately			
			4	Quite a bit			
			5	Extremely			
			97/98/99	Missing			
	BodilyPain	How much bodily pain have you had during the past 4 weeks?'	1	None			
			2	Very mild			
			3	Mild			
			4	Moderate			
			5	Severe			
			6	Very severe			
			97/98/99	Missing			
	PainInterfereWithWork	During the past 4 weeks, how much did pain interfere with your normal work (includi	1	Not at all			
			2	A little bit			
			3	Moderately			
			4	Quite a bit			
			5	Extremely			
			97/98/99	Missing			
	FeelFullOfPep_FeelingResponses	How much time during the past 4 weeks have/did you ...?'	1	All of the Time			
	FeelNervous_FeelingResponses		2	Most of the Time			
	FeelDown_FeelingResponses		3	A Good Bit of the Time			
	FeltCalm_FeelingResponses		4	Some of the Time			
	ALotOfEnergy_FeelingResponses		5	A Little of the Time			
	FeltDownhearted_FeelingResponses		6	None of the Time			
	FeelWornOut_FeelingResponses		97/98/99	Missing			
	BeenHappy_FeelingResponses						
	FeelTired_FeelingResponses						
	HowOftenHealthInterferedActivities	During the past 4 weeks, how much of the time has your physical health or emotional	1	All of the time			
			2	Most of the time			
			3	Some of the time			
			4	A little of the time			
			5	None of the time			
			97/98/99	Missing			
	SickEasier_HealthExpectation	How True or False is each of the following statements for you?'	1	Definitely True			
	HealthyasAnybody_HealthExpectation		2	Mostly True			
	HealthToGetWorse_HealthExpectation		3	Don't Know			
	HealthIsExcellent_HealthExpectation		4	Mostly False			
			5	Definitely False			
			97/98/99	Missing			
	CALC_AvgSF36PhysicalFunctioning	total SF36 score for each domain	number				
	CALC_AvgSF36RoleLimitationPhysicalHealth						
	CALC_AvgSF36RoleLimitationEmotionalProblems						
	CALC_AvgSF36EnergyOrFatigue						
	CALC_AvgSF36EmotionalWellbeing						
	CALC_AvgSF36SocialFunctioning						
	CALC_AvgSF36Pain						
	CALC_AvgSF36GeneralHealth						
	CALC_AvgSF36HealthChange						

Required Field (please X)	Variable	Variable Label	Values	Value Label	comment
	MoCAlternatingTrailMaking	number letter sequencing	1	correct	
	MoCAVisuoconsturctinalCube	completion of 3D cube	2	incorrect	
	MoCAVisuoconsturctinalContour	drawing of clock face outline	97/98/99	Missing	
	MoCAVisuoconsturctinalHands	placement of clock hands			
	MoCAVisuoconsturctinalNumbers	placement of clock numbers			
	MocANamingLion		1	correct	
	MocANamingRhino		2	incorrect	
	MocANamingCamel		97/98/99	Missing	
	MoCADigitsBackward	repetition of digits forward	1	correct	
	MoCADigitsForward	repetition of digits backwards	2	incorrect	
			97/98/99	Missing	
	MoCAVigalence	recognition of 'A' in letter sequence	1	0-1 error	
			2	>1 error	
			97/98/99	Missing	
	MoCASerial	number of correct subtractions of 7 starting	0	correct subtractions	
			1		
			2		
			3		
			4		
			5		
			97/98/99	Missing	
	MoCASentenceRepetitionOne	repeat sentence	1	correct	
	MoCASentenceRepetitionTwo	repeat sentence	2	incorrect	
			97/98/99	Missing	
	MoCAVerbalFluency	tell me as many words as you can beginning	number		
			97/98/99	Missing	
	MoCAAbstractionOne	what is the likeness of train and bicycle?	1	correct	
	MoCAAbstractionTwo	what is the likeness of ruler and watch	2	incorrect	
			97/98/99	Missing	
	MoCADelayedRecallFace	recall of words read earlier	1	recalled	
	MoCADelayedRecallVelvet		2	not recalled	
	MoCADelayedRecallChurch		3	incorrect	
	MoCADelayedRecallDaisy		97/98/99	Missing	
	MoCADelayedRecallRed				
	MoCAOrientationDate	orientation to time and place	1	correct	
	MoCAOrientationMonth		2	incorrect	
	MoCAOrientationYear		97/98/99	Missing	
	MoCAOrientationDay				
	MoCAOrientationPlace				
	MoCAOrientationCity				
	MoCAOrientationEducation	years of formal education	1	<13	
			2	>=13	
			97/98/99	Missing	
	CALC_MoCA	score out of 30 >=26 'normal'	0-30		
			97/98/99	Missing	

Required Field (please X)	Variable	Variable Label	Values	Value Label comment
	MyocardialInfarction	Have you ever had a heart attack	1	yes
	CongestiveHeartFailure	Have you ever been treated for heart failure?	2	no
	PeripheralVascularDisease	Have you had an operation to unclog or bypass the art	3	not sure
	CerebrovascularAccident	Have you had a stroke, cerebrovascular accident, bloo	97/98/99	Missing
	Hemiplegia	Do you have difficulty moving an arm or leg as a result of the stroke or cerebrovascular accident?		
	Asthma	Do you have asthma?		
	AsthmaMedicines	Do you take medications for your asthma?'	2	no
			1	yes, only with flare ups of my asthma
			3	yes, I take medicine regularly even when I'm not having a flare up
			97/98/99	Missing
	emphysema	Do you have emphysema, chronic bronchitis, or chron	1	yes
			2	no
			3	not sure
			97/98/99	Missing
	emphysemaMedicines	Do you take medicines for your lung disease?'	2	no
			1	yes, only with flare ups
			3	yes, I take medicine regularly even when I'm not having a flare up
			97/98/99	Missing
	UlcerDisease	Do you have stomach ulcers or peptic ulcer disease diagnosed by endoscopy or upper gi or barium swallow study?		
	HasDiabetes	Do you have diabetes?	1	Yes, treated by modifying my diet
			2	No
			3	Yes, treated by medications taken by mouth
			4	Yes, treated by insulin
			97/98/99	Missing
	DiabetesCausedProblems	Has the diabetes caused any of the following problem:	1	Problems with kidneys
			2	Problems with eyes, treated by an ophthalmologist
			3	unsure
			97/98/99	Missing
	ProblemsWithKidneys	Have you ever had problems with your kidneys?'	1	Poor kidney function (blood tests show high creatinine)
			2	No
			3	Have used hemodialysis or peritoneal dialysis
			4	Have received kidney transplantation
			97/98/99	Missing
	ConnectiveTissueDisese	Do you have Rheumatoid Arthritis?	1	Yes
			2	No
			3	Not sure
			97/98/99	Missing
	ConnectiveTissueDiseaseMedications	Do you take medications for it regularly?'	1	Yes
			2	No
			3	Not sure
			97/98/99	Missing
	**ConnectiveTissueDiseaseType**	do you have....?'	1	Lupua
			2	Polymyalgia rheumatica
			3	neither lupus nor polymalgia
			4	not sure
			97/98/99	Missing
	**Dementia**	Do you have any of the following conditions..?	1	Yes
	**Cirrhosis**		2	No
	**Leukemia**		3	Not sure
	**Lymphoma**		97/98/99	Missing
	**Aids**			
	**Cancer**			
		Has the cancer spread or metastasized to other parts c	1	Yes
	**CancerSpread**		2	No
			3	Not sure
			97/98/99	Missing
	**CALC_KatzCormbidity**	total score plus age based on the Charlson index scori	number	
	**Hypertension**	Does the participant's GP record identify...	1	Yes
	**1b. Any atherosclerotic disease**		2	No
	**1c. Ischaemic heart disease**		3	Not sure
	**Any atherosclerotic disease**		2	No
	**Ischaemic heart disease**		97/98/99	Missing
	**Cerebrovascular disease**			
	**Peripheral vascular disease**			
	**Heart failure**			
	**Atrial fibrillation**			
	**Atrial flutter**			
	**Osteoarthritis**			
	**Cervical or lumbar spondylosis**			
	**Rheumatoid arthritis**			
	**Other arthritis (specified)**			
	**Arthritis (type not specified)**			
	**Joint replacement**			
	**Cataract**			
	**Cataract surgery**			
	**Age related macular degeneration**			
	**Glaucoma Diabetic eye disease**			
	**Registered blind**			
	**Registered partially sighted**			
	**Any cancer**			
	**Any cancer, excluding nonmelanoma skin ca**			
	**Any cancer <5 years since diagnosis**			
	**Chronic obstructive pulmonary disease (COP**			
	**Asthma**			
	**Other respiratory disease**			
	**Diabetes mellitus**			
	**Hypothyroidism**			
	**Hyperthyroidism**			
	**Dementia**			
	**Parkinson's disease**			
	**Anxiety**			
	**Depression**			
	**Anaemia: WHO criteria for haemoglobin con**			
	**Anaemia: Joosten's criterion for haemoglobi**			
	**Osteoporosis**			
	**Fractured hip, wrist or backbone**			
	**Renal function using modification of diet in r**		1	Normal/mildly reduced
			2	Moderately reduced (stage 3††)
			3	Severely reduced (Stage 4††)
			4	Very severely reduced (stage 5††)
			97/98/99	Missing
	**Calculate total number of diseases**	Total number of diseases from GP record (excluding os	Number	
			97/98/99	Missing

Required Field (please X)	Variable	Variable Label	Values	Value Label	comment
	**FallsInLast12Months**	in the last 12 months have you had a fall	1	Yes	
			2	no	
			3	not sure	
			97/98/99	missing	
	**HowManyFalls**	How mnay time s have you fallen in the l	number		
			97/98/99	missing	
	**FallsFractures**	...have you broken any bones/had and fra	1	Yes	
			2	no	
			3	not sure	
			97/98/99	missing	
	**FallsBrokenBones**	How many times has a fall resulted in a b	number		
			97/98/99	missing	
	**FallsAccidentEmergency**	...did you go to Aand E follwing a fall?	1	Yes	
			2	no	
			3	not sure	
			97/98/99	missing	
	**FallsGetAandE**	How many times did you attend A&E	number		
			97/98/99	missing	
	**FallsStayOvernight**	...were you admitted to a hospital follow	1	Yes	
			2	no	
			3	not sure	
			97/98/99	missing	
	**FallsGetAdmited**	How many times were you admitted to a	number		
			97/98/99	missing	
	**FallsSeenGP**	Have you (or your carer) ever seen your (	1	Yes	
			2	no	
			3	not sure	
			97/98/99	missing	
	**FallsSpecialist**	Have you ever seen a falls specialist?'	1	Yes	
			2	no	
			3	not sure	
			97/98/99	missing	
	**LossOfConfidence**	Have your falls caused any of the followi	1	Yes	
	**WorryAboutFalling**		2	no	
	**GoingOutLessOften**		3	not sure	
	**IncreaseCareReceive**		97/98/99	missing	

Required Field (please X)	Variable	Variable Label	Values	Value Label	comment
	TookPresMed	Number of prescribed medications	number		
			97/98/99	Missing	
	Medication*_MedicationName	Name of prescribed medication 1-30	verbatim		
			97/98/99	Missing	
	Medication*_Dosage	Dosage of prescribed medicationsverbatim	number		
			97/98/99	Missing	
	Medication*_Frequency	Frequency of prescribed medication	number		
			97/98/99	Missing	
	**TookNonPresMed**	Number of non-prescribed medicines, vitamin or mineral supplement	number		
			97/98/99	Missing	
	**NonPresMedication*_NonPresMedicationName**	Name of non-prescribed medication 1-30	verbatim		
			97/98/99	Missing	
	**NonPresMedication*_NonPresMedicationDosage**	Dosage of non-prescribed medicationsverbatim	number		
			97/98/99	Missing	
	**NonPresMedication*_NonPresMedicationFrequency**	Frequency of non-prescribed medication	number		
			97/98/99	Missing	
	**COUNT_PrescribedMedication**		number		
	**COUNT_NonPrescribedMedication**		number		

Required Field (please X)	Variable	Variable Label	Values	Value Label	comment
	WalkAroundOutside_NEADLPartOne	...what have you *actually*done in the l	0	not at all	
	ClimbStairs_NEADLPartOne		1	with help	
	GetInAndOutOfCar_NEADLPartOne		2	on your own with difficulty	
	WalkOverUnevenGround_NEADLPartOne		3	on your own	
	CossRoads_NEADLPartOne		97/98/99	Missing	
	TravelOnPublicTransport_NEADLPartOne				
	ManageToFeedYourself_NEADLPartOne				
	MakeYourselfAHotDrink_NEADLPartOne				
	TakeHotDrinksFromOneDrinksToAnother_NEADLPartOne				
	DoTheWashingUp_NEADLPartOne				
	MakeYourselfAHotSnack_NEADLPartOne				
	ManageOwnMoney_NEADLPartTwo	...what have you *actually*done in the l	0	no	
	WashItemsOfClothing_NEADLPartTwo		1	with help	
	DoOwnHousework_NEADLPartTwo		2	on your own with difficulty	
	DoOwnShopping_NEADLPartTwo		3	on your own	
	DoFullClothesWash_NEADLPartTwo		97/98/99	Missing	
	ReadNewspapersOrBooks_NEADLPartTwo				
	UseTelephone_NEADLPartTwo				
	WriteLetters_NEADLPartTwo				
	GoOutSocially_NEADLPartTwo				
	ManageGarden_NEADLPartTwo				
	Drive_NEADLPartTwo				
	CALC_NEADL	Total NEADL. Higher score = more ind	number 0-66		
			97/98/99	Missing	
	Feeding	Do you have any difficulty with the fol	0	uanble	
			1	needs help cutting, spreading butter or requires modified diet	
			2	independent	
			97/98/99	Missing	
	Bathing		0	dependent	
			1	independent (or in shower)	
			97/98/99	Missing	
	Grooming		0	needs help with personal care	
			1	independent face/hair/teeth/shaving	
			97/98/99	Missing	
	Dressing		0	dependent	
			1	needs help but can do about half unaided	
			2	independent including buttons and zips	
			97/98/99	Missing	
	Bowels		0	incontinent or needs enemas	
			1	occasional accident	
			2	continent	
			97/98/99	Missing	
	Bladder		0	unable (or catherterised and unable to manage alone)	
			1	occasional accident	
			2	continent	
			97/98/99	Missing	
	ToiletUse		0	dependent	
			1	needs some help but can so somethings alone	
			2	independent (on/off an wiping)	
			97/98/99	Missing	
	Transfers		0	unable, no sitting balance	
			1	major help (one or two people physical) can sit up	
			2	minor help (verbal or physical)	
			3	independent	
			97/98/99	Missing	
	Mobility		0	immobile or <50 yards	
			1	wheelchair independent including corners >50 yards	
			2	walks with the help of one person (verbal or physical) >50 yards	
			3	independent but may use any walking aide >50 yards	
			97/98/99	Missing	
	Stairs		0	unable	
			1	needs help (verbal physical, carrying aid)	
			2	independent	
			97/98/99	Missing	
	CALC_Barthel_Index	Total BARTHEL. Higher score means m	number 0-20		
			97/98/99	Missing	

Required Field (please X)	Variable	Variable Label	Values	Value Label	comment
	HeightWeighttimeofDay	time measurements are taken	hh.mm		
	Demispan	from middel of collar bone to index finger	number cm		
	DemispanHeight	Calculation of height from demispan	number cm		
	Height		number cm		
	Weight		number kg		
	BodyFatPercentage		number %		
	FatMass		number kg		
	FatFreeMass		number kg		
	BodyWaterPercentage		number %		
	MuscleMass		number kg		
	BoneMass		number kg		
	CALC_DemiSpanHeight				
	CALC_BMI	mass (kg) /demi span height (m)2	number		
	WeightLoss	In the last year, have you lost more than 10lb	1	yes	
			2	no	
			3	not sure	
			97/98/99	Missing	
	SittingBloodPresssure		yes	1	
			no	2	
			97/98/99	Missing	
	ReasonNoSittingBloodPressure		verbatim		
			1	Participant refused	
			3	Cuff the wrong size	
			4	Monitor malfunction	
			5	StandingBloodPressureTaken	
	SittingSystolic	BP reading	number mm Hg		
	Sitting Diastolic	BP reading	97/98/99	Missing	
	SittingPulse	pulse			
	StandingBloodPresssure		yes	1	
			no	2	
			97/98/99	Missing	
	ReasonNoStandingBloodPressure		verbatim		
	StandingSystolic	BP reading	number mm Hg		
	Standing Diastolic	BP reading	97/98/99	Missing	
	StandingPulse	pulse			
	DominantHand	Which is the dominant hand?	1	right	
			2	left	
	DominantHandFirstAttempt	grip strength in dominant hand	number kPA		
	DominantHandSecondAttempt				
	DominantHandThirdAttempt				
	NondominantHandFirstAttempt	grip strength in passive hand	number kPA		
	NondominantHandSecondAttempt				
	NondominantHandThirdAttempt				
	CALC_DominantMeanGripStrength	mean grip strength dominant hand	number kPA		
	CAL_NonDominantMeanGripStrength	mean grip strength passive hand			

Required Field (please X)	Variable	Variable Label	Values	Value Label	comment
	WalkTime	over a 3 metre distance	number seconds		
			97/98/99	Missing	
	ExhaustionPartA	How often in the last week did you feel 'everything I did was an effort?'	0	rarely or none of the time	
			1	some or a little of the time	
			2	moderate amount of the time	
			3	most of the time	
			97/98/99	Missing	
	ExhaustionPartB	How often in the last week did you feel 'I could not get going'?'	0	rarely or none of the time	
			1	some or a little of the time	
			2	moderate amount of the time	
			3	most of the time	
			97/98/99	Missing	
	TUGTSkipped	was the TUGT missed out?	1	yes	
			2	no	
	TUGNotDoneReason	reason for skipping TUGT	verbatim		
	chairUsed		1	yes	
			2	no	
	ChairHeight	height of the chair used to stand up from	number mm		
	AbleToGetUpFromChair		1	yes	
			2	no	
			97/98/99	Missing	
	TimeToGetUp	time taken to complete in seconds	number secs.		
			97/98/99	Missing	
	**WalkingAidUsed**	walking aid used to complete the TUGT	1	Independent	
			2	1x walking stick	
			3	2x walking sticks	
			4	Walking Zimmer frame	
			5	Wheeled Zimmer frame	
			6	3-wheeled walker	
			7	4-wheeled walker	
			8	Kitchen trolley	
			97/98/99	Missing	

Required Field (please X)	Variable	Variable Label	Values	Value Label comment
	ClinicalFrailtyResearcher	Researcher frailty assessment based on observatio	1	very fit; robust, active, energetic, well motivated and fit. These people commonly exercise regularlry and are in the most fit group for their age
			2	well; without active disease, but less fit htan people in category 1
			3	well; with terated comorbid disease. Disease symptoms are controlled compared with those in category 4
			4	apparently vulnerable; although not frankly dependent, these people commonly complain of beign 'slowed up' or have disease symptoms
			5	mildly frail; with limited dependence on others for instrumental activities of daily living
			6	moderately frail; help is needed with both instrumental and non-instrumental activities of daily living
			7	severely frail; completely dependent on others for the activities of daily living or terminally ill
			99	missing
	**ClinicalFrailtyIsClinicianPresent**	Is a registrar present to complete 2nd part of the a	1	yes
			2	no
	**ClinicalFrailtyClinican**	Clinician frailty assessment based on observation	1	very fit; robust, active, energetic, well motivated and fit. These people commonly exercise regularlry and are in the most fit group for their age
			2	well; without active disease, but less fit htan people in category 1
			3	well; with terated comorbid disease. Disease symptoms are controlled compared with those in category 4
			4	apparently vulnerable; although not frankly dependent, these people commonly complain of beign 'slowed up' or have disease symptoms
			5	mildly frail; with limited dependence on others for instrumental activities of daily living
			6	moderately frail; help is needed with both instrumental and non-instrumental activities of daily living
			7	severely frail; completely dependent on others for the activities of daily living or terminally ill
			99	missing
	CALC_FriedTotalScore	Fried frailty score	0	not frail
			1	pre frail
			2	pre frail
			3	mild frailty
			4	moderate frailty
			5	severe frailty
			97/98/99	missing
	PenFromHand	Can you take this oen from my hand if I hold it her	1	yes
			2	no
			97/98/99	missing
	BustrainTimetable	Are you able to use a bus or train timetable?'	1	yes
			2	no
			97/98/99	missing
	SitUprightforTwoHours	Are you able to sit upright in a chair for two hours'	1	yes
			2	no
	CoinFromTable	Are you able to pick up a 10p coin from a table?'	1	yes
			2	no
			97/98/99	missing
	EFSCognition	draw 10 past 11 on clock face	0	no errors
			1	minor spacing errors
			2	other errors
			97/98/99	missing
	EFSAdmitted	How many hospital admissions in the past year	0	0
			1	1 or 2
			2	>2
			97/98/99	missing
	EFSSocialSupport	can you count on someone?	0	always
			1	sometimes
			2	never
			97/98/99	missing
	EFSForgetMedication	At times do you forget to take your prescription m	0	no
			1	yes
			97/98/99	missing
	EFSNutrition	Have you lost weight and clothes become loose?	0	no
			1	yes
			97/98/99	missing
	EFSMood	Do you often feel sad or depressed?'	0	no
			1	yes
			97/98/99	missing
	EFSContinence	Do you have a problem losing control of urine whe	0	no
			1	yes
			97/98/99	missing
	CALC_EdmontonFrailScale	total score out of 17	0-5	not frail
			6-7	vulnerable
			8-9	mild frailty
			10-11	moderate frailty
			12-17	severe frailty
			97/98/99	missing
	EFIGPScore	data extracted from GP summary records		
	Fit (eFI score 0 - 0.12)	: People who have no or few long-term conditions that are usually well controlled. This group would mainly be independent in day to day living activities.		
	Mild frailty (eFI score 0.13 – 0.24)	: People who are slowing up in older age and may need help with personal activities of daily living such as finances, shopping, transportation.		
	Moderate Frailty (eFI score 0.25 – 0.36)	: People who have difficulties with outdoor activities and may have mobility problems or require help with activites such as washing and dressing.		
	Severe Frailty (eFI score > 0.36)	: People who are often dependent for personal cares and have a range of long-term conditions/multimorbidity. Some of this group may be medically stable but others can be unstable and at risk of dying within 6 - 12 months		
	99	missing		
	CALC_ELSAFrailtyIndex			
	0-10	very fit		
	11-14	well		
	15-24	vulnerable		
	>=25	frail		
	missing 99			
	http://www.elsa-project.ac.uk/publicationDetails/id/7167			

Required Field (please X)	Variable	Variable La	Values	Value Label	comment		
	Sleep	Have you sl	1	Yes			
			2	No			
			3	Not sure			
			97/98/99	Missing			
	HealthRelatedMobility	EQ5D	1	no problems walking about			
			2	slight problems			
			3	moderate problems			
			4	severe problems			
			5	unable to walk			
			97/98/99	Missing			
	HealthRelatedSelfCare		1	no problems washing or dressing			
			2	slight problems			
			3	moderate problems			
			4	severe problems			
			5	unable to wash or dress			
			97/98/99	Missing			
	HealthRelatedUsualActivities		1	no problems doing usual activities			
			2	slight problems			
			3	moderate problems			
			4	severe problems			
			5	unable to do usual activities			
			97/98/99	Missing			
	HealthRelatedPain		1	no pain or discomfort			
			2	slight pain or discomfort			
			3	moderate pain or discomfort			
			4	severe pain or discomfort			
			5	extreme pain or discomfort			
			97/98/99	Missing			
	HealthRelatedAnxiety		1	not anxious or depressed			
			2	slightly anxious or depressed			
			3	moderately anxious or depressed			
			4	severely anxious or depressed			
			5	extremely anxious or depressed			
			97/98/99	Missing			
	CALC_EQ5D5L	total EQ5D score					

Required Field (please X)	Variable	Variable Label	Values	Value Label	comment
	PainStoppedActivity	has pain…	0	no	
	PainStoppedClimbing		2	yes	
	PainStoppedWalkingMoreThan200		97/98/99	Missing	
	PainStoppedWalkingLessThan200				
	PainCutDownActivities				
	PainAccomplishingLess				
	PainLimitedWork				
	PainWorkRequireExtraEffort				
	PainTroubleSleeping				
	PainPreventSocialActivities				
	PainToday	on a scale of 0-10 with 0	number 0-10		
	PainLast7Days		97/98/99	Missing	
	CALC_GeriatricPainMeasure	Geriatric Pain Measure t	number 0-40		
			97/98/99	Missing	

Required Field (please X)	Variable	Variable Label	Values	Value Label	comment		
	SomeoneToTalkTo_LoninessScale	...the extent to which the statements apply	1	yes			
	MissHavingCloseFriend_LoninessScale		2	more or less			
	SenseOfEmptiness_LoninessScale		3	no			
	PeopleICanLeanOn_LoninessScale		97/98/99	missing			
	MissCompanyOfOthers_LoninessScale						
	CircleOfFriendsLimited_LoninessScale						
	PeopleITrustCompletely_LoninessScale						
	PeopleIFeelCloseTo_LoninessScale						
	MissHavingPeopleAround_LoninessScale						
	FeelRejected_LoninessScale						
	CallFriendsWheneverINeed_LoninessScal						
	CALC_emotionalLoneliness	yes or more or less on ? 2,3,5,6,9,10	number				
	CALC_missingemotionalLoneliness	number of mising items					
	CALC_socialLoneliness	no or more or less on ?1,4,7,8,11					
	CALC_misssingsocialLoneliness	number of missing items					
	CALC_totalLonliness	De Jong Gierveld scale sum of emotional and social loneliness	0-2	not lonely			
			3-8	moderately lonely			
			9-10	severely lonely			
			11	very serverly lonely			
			97/98/99	Missing			
	CALC_LonelinessScore	loneliness category	1	Not lonely			
			2	Moderately lonely			
			3	Severely lonely			
			4	Very severely lonely			
			97/98/99	Missing			
1	SatisfiedWithLife	basically satisfied with life	0	yes		1	yes
2	DroppedActivities	dropped many activities and interests	1	no		0	no
3	FeelEmpty	feel life is empty	97/98/99	Missing		97/98/99	Missing
4	GetBored	often get bored					
5	GetBored2 -?goodspirits?	in good spirits most of the time					
6	SomethingBadGoingToHappen	afraid that something bad is going to happen					
7	FeelHappy	feel happy most of the time					
8	FeelHelpless	often feel helpless					
9	PreferToStayAtHome	prefer to stay at home rather than going out and doing things					
10	ProblemsWithMemory	more problems with memory than most					
11	WonderfulToBeAlive	think it's wonderful to be alive					
12	Worthless	worthless the way you are now					
13	FullOfEnergy	full of energy					
14	SituationIsHopeless	situation is hopeless					
15	BetterOffThanYou	most people better off than you					
	GeriatricDepressionScale	total from 15 questions reverse score for items 1,5,7,11,13 >=5 suggests depressed	number 0-15				
			97/98/99	Missing			

Required Field (please X)	Variable	Variable Label	Values	Value Label comment	
	BounceBackQuickly_BriefResilienceScale	agreement with resiliance	1	strongly agree	
	HardTimeThroughStressfulEvents_BriefResilienceScale		2	agree	
	LongToRecoverFromStress_BriefResilienceScale		3	neutral	
	SnapBackFromSomethingBad_BriefResilienceScale		4	disagree	
	DifficultTimesLittleTrouble_BriefResilienceScale		5	strongly disagree	
	TimeToGetOverSetBacks_BriefResilienceScale		97/98/99	Missing	
	CALC_briefResilianceScale	sum/6 items reverse scorir	number 1-6 higer=more resiliant		
			97/98/99	Missing	
	SolveDifficultProblems_SelfEfficacyScale	agreement with general se	1	not at all true	
	WaysToGetWhatIWant_SelfEfficacyScale		2	hardly true	
	AccomplishMyGoals_SelfEfficacyScale		3	moderately true	
	DealWithUnexpectedEvents_SelfEfficacyScale		4	exactly true	
	HandleUnforeseenSituations_SelfEfficacyScale		97/98/99	Missing	
	SolveMostProblems_SelfEfficacyScale				
	RemainCalm_SelfEfficacyScale				
	FindSeveralSolutions_SelfEfficacyScale				
	ThinkOfASolution_SelfEfficacyScale				
	HandleWhateverComes_SelfEfficacyScale				
	CALC_SelfEfficacyScale	Total self-efficacy score- hi[illegible]	number 10-40		
			97/98/99	Missing	

NB: Individual items in ELSA are pulled from other assessm				
WalkingOneBlock_SF-36Activities				
SitUprightForTwoHours				
AbleToGetUpFromChair				
ClimbingSeveralStairs_SF-36Activities				
ClimbingStairs_SF-36Activities				
BendingKneelingStooping_SF-36Activities				
PenFromHand				
ModerateActivities_SF-36Activities				
VigorousActivities_SF-36Activities				
CoinFromTable				
HealthRelatedSelfCare				
HealthRelatedMobility				
Bathing				
Feeding				
Transfers				
ToiletUse				
BustrainTimetable				
MakeYourslefAHotSnack				
DoOwnShopping_NEADLPartTwo				
UseTelephone_NEADLPartTwo				
EFSForgetMedication				
ManageOwnMoney_NEADLPartTwo				
DoOwnHousework_NEADLPartTwo				
ExhaustionPartA				
Sleep				
GoodSpirits				
WonderfulToBeAlive				
FeelHappy				
ExhaustionPartB				
Hypertension				
Atherosclerotic				
MyocardialInfarction				
HeartFailure				
DiabetesMellitus				
Cerebrovascular				
COPD				
RespiratoryAsthma				
Osteoporosis				
ExcludingNonmelanoma				
Parkinson				
NeurologicalDementia				
eyesight 4 & 5				
hearing & vision 6 & 7				
FallsInLast12Months				
FracturedHipWristBackbone				
Joint				
geriatric pain measure 3 or				
MoCAOrientationDate				
MoCAOrientationMonth				
MoCAOrientationYear				
MoCAOrientationDay				
MoCASentenceRepetitionOne				
MoCAVerbalFluency				
delayed recall combines 5 questions				
SF-36HealthSurvey'				
GeriatricDepressionScale >= 5				
CALC_totalLonliness) >= 3				
AtrialFibrillation				
AtrialFlutter				
Osteoarthritis				
RheumatoidArthritis				
OtherArthritis				
Arthritis				
Anxiety				
Depression				
RecognisingPeople				
ReadingNewsprint				
HearingInQuietRoom				
HearingInLoudRoom				
PainStoppedWalkingMoreThan200				
PainStoppedWalkingLessThan200				
MoCADelayedRecallFace				
MoCADelayedRecallVelvet				
MoCADelayedRecallChurch				
MoCADelayedRecallDaisy				
MoCADelayedRecallRed				
CALC_ELSAFrailtyIndex				

NB: Individual items in Fried are pulled from other assessment data where possible to avoid overburdening the participant								
CALC_DemiSpanHeight								
CALC_BMI								
WalkTime								
WeightLoss								
ExhaustionPartA								
ExhaustionPartB								
DominantHand								
DominantHandFirstAttempt								
DominantHandSecondAttempt								
DominantHandThirdAttempt								
NonDominantHandFirstAttempt								
NonDominantHandSecondAttempt								
NonDominantHandThirdAttempt								
CALC_DominantMeanGripStrength								
CALC_NonDominantMeanGripStrength								
CALC_FriedTotalScore								
