# Supplementary material for: Community ageing research 75+ study (CARE75+): an experimental ageing and frailty research cohort
Source: BMJ Open. 2019 Mar 7;9(3):e026744. doi: 10.1136/bmjopen-2018-026744 (PMC6429944; doi:10.1136/bmjopen-2018-026744)
Supplement: Supplementary file 2 [file bmjopen-2018-026744supp002.pdf]

Enquiries to lesley.brown@bthft.nhs.uk
Project number: [office use only]

CARE75+ DATA REQUEST FORM

This form is to be used for all data request purposes including; sampling, preparatory work and research.

The request contact will be responsible for the transfer, storage and governance of the data in line with the data sharing agreement (appendix 1).

All sections *must* be completed.

1. Office use only

Data request number	
Date of request	
Date of review by DRRC	
Date of DRRC query	
Date of query review	
Data sent	

2. Request contact

Study Name:	
Requested by:	
Contact:	
Email	
Telephone	
Job role	
Organisation	
Date of request	
Date required	

Enquiries to lesley.brown@bthft.nhs.uk
Project number: [office use only]

3. Data Handling. If anyone else is expected to handle (i.e. view/analyse/transfer/store) these data in association with the study named in section 1, please list them here:

Name	Affiliation	Title	Role in the project

4. Purpose of request

Sampling i.e. participant contacts	
Scoping exercise e.g feasibility/protocol development	
Research e.g. analysis for funded/approved projects	

5. Research question and brief summary of research (350 words)

6. What type of data do you require?

Individual identifiable data (contains personal details)	
Pseudo-anonymised (contains unique id for data linkage)	
Anonymised (contains no identifiable details)	

7. What stage do you require (NB: full data will not be available for all participants)

Baseline	
Six month follow-up	
12 month follow-up	
24 month follow-up	
48 month follow-up	
Latest time-point	

8. Selection criteria

Included if:	
But excluded if:	

9. Specific data items required

Date of Assessment required?		
**Data dictionary sheet title**	**Variable name** (please cut and paste from data dictionary)	
Contact information		
Personal details		
Housing, Living Circumstance		
Education, Occupation		
Family Data		
Formal and Informal Support		
Smoking, Alcohol		
Hearing, Eyesight		
SF-36		

Enquiries to lesley.brown@bthft.nhs.uk
Project number: [office use only]

MoCA	
Co-morbidities	
Falls	
Medications	
Activities of Daily Living	
Height, Weight, BP, Grip	
Timed Up and Go, Walking	
Frailty	
Quality of Life and Sleep	
Pain	
Loneliness, Depression	
Resilience, Self-Efficacy	

10. Please can you provide details of your intended output (for example, publication or report)
