# Supplementary material for: Community ageing research 75+ study (CARE75+): an experimental ageing and frailty research cohort
Source: BMJ Open. 2019 Mar 7;9(3):e026744. doi: 10.1136/bmjopen-2018-026744 (PMC6429944; doi:10.1136/bmjopen-2018-026744)
Supplement: Author's manuscript [file bmjopen-2018-026744.draft_revisions.pdf]

BMJ Open

BMJ Open is committed to open peer review. As part of this commitment we make the peer review history of every article we publish publicly available.

When an article is published we post the peer reviewers' comments and the authors' responses online. We also post the versions of the paper that were used during peer review. These are the versions that the peer review comments apply to.

The versions of the paper that follow are the versions that were submitted during the peer review process. They are not the versions of record or the final published versions. They should not be cited or distributed as the published version of this manuscript.

BMJ Open is an open access journal and the full, final, typeset and author-corrected version of record of the manuscript is available on our site with no access controls, subscription charges or pay-per-view fees (http://bmjopen.bmj.com).

If you have any questions on BMJ Open's open peer review process please email info.bmjopen@bmj.com

BMJ Open

The Community Ageing Research 75+ Study (CARE75+): an experimental ageing and frailty research cohort

Journal:	*BMJ Open*
Manuscript ID	bmjopen-2018-026744
Article Type:	Protocol
Date Submitted by the Author:	19-Sep-2018
Complete List of Authors:	Heaven, Anne; Bradford Institute for Health Research, Academic Unit of Elderly Care & Rehabilitation<br>Brown, Lesley; Bradford Institute for Health Research, Academic Unit of Elderly Care & Rehabilitation<br>Young, John; University of Leeds, Academic Unit of Elderly Care & Rehabilitation<br>Teale, Elizabeth; University of Leeds, Academic Unit of Elderly Care & Rehabilitation<br>Hawkins, Rebecca; University of Leeds, Academic Unit of Elderly Care and Rehabilitation<br>Splisbury, Karen; University of Leeds, School of Healthcare<br>Mountain, Gail; University of Bradford, Centre for Applied Dementia Studies<br>Young, T; The University of Sheffield, School of Health and Related Research<br>Goodwin, Victoria; University of Exeter, PenCLAHRC<br>Hanratty, Barbara; Newcastle University, Institute of Health and Society<br>Chew-Graham, Carolyn; University of Keele, Research Institute for Primary Care & Health Sciences<br>Brundle, Caroline; Bradford Institute for Health Research, Academic Unit of Elderly Care & Rehabilitation<br>Mahmood, Farhat; Bradford Institute for Health Research, Academic Unit of Elderly Care & Rehabilitation<br>Jacob, Ikhlaq; Bradford Institute for Health Research, Academic Unit of Elderly Care & Rehabilitation<br>Daffu-O'Reilly, Amrit; University of Leeds, Academic Unit of Midwifery, Social Work, Pharmacy and Counselling and Psychotherapy<br>Clegg, Andrew; University of Leeds, Academic Unit of Elderly Care & Rehabilitation
Keywords:	frailty, CARE75+, cohort, TwiCs, ageing

SCHOLARONE™
Manuscripts

The Community Ageing Research 75+ Study (CARE75+): an experimental ageing and frailty research cohort

Authors: Anne Heaven[1], Lesley Brown[1], John Young[1], Elizabeth Teale[1], Rebecca Hawkins[1], Karen Spilsbury[2], Gail Mountain[3], Tracey Young[4], Victoria Goodwin[5], Barbara Hanratty[6], Carolyn A. Chew Graham[7], Caroline Brundle[1], Farhat Mahmood[1], Ikhlaq Jacob[1], Amrit Daffu-O'Reilly[8], Andrew Clegg[1]

[1]Academic Unit of Elderly Care & Rehabilitation, University of Leeds, Bradford Institute for Health Research, Bradford Teaching Hospitals NHS Foundation Trust, Bradford, BD9 6RJ, and NIHR CLAHRC Yorkshire & Humber

[2]Academic Unit: Adult, Child and Mental Health Nursing, School of Healthcare, University of Leeds, LS2 9JT, and NIHR CLAHRC Yorkshire & Humber

[3]Centre for Applied Dementia Studies, Faculty of Health Studies, Bradford University, BD7 1DP, and NIHR CLAHRC Yorkshire & Humber

[4] School of Health and Related Research, University of Sheffield, Regent Court, 30 Regent St, Sheffield, S1 4DA, and NIHR CLAHRC Yorkshire & Humber

[5] NIHR CLAHRC South West Peninsula, University of Exeter Medical School, EX1 2LU

[6] Institute of Health & Society, Newcastle University Institute for Ageing, Biomedical Research Building, Newcastle University, NE4 5PL

[7]Research Institute for Primary Care & Health Sciences, Keele University, Staffordshire ST5 5BG and NIHR CLAHRC West Midlands

[8] Academic Unit of Midwifery, Social Work, Pharmacy and Counselling and Psychotherapy, University of Leeds, LS2 9JT

Email addresses

anne.heaven@bthft.nhs.uk

lesley.brown@bthft.nhs.uk

For peer review only

For peer review only - http://bmjopen.bmj.com/site/about/guidelines.xhtml

john.young@bthft.nhs.uk

elizabeth.teale@bthft.nhs.uk

r.hawkins@leeds.ac.uk

k.spilsbury@leeds.ac.uk

g.mountain@bradford.ac.uk

t.a.young@sheffield.ac.uk

v.goodwin@exeter.ac.uk

barbara.hanratty@newcastle.ac.uk

c.a.chew-graham@keele.ac.uk

caroline.brundle@bthft.nhs.uk

farhat.mahmood@bthft.nhs.uk

ikhlaq.jacob@bthft.nhs.uk

a.k.daffuoreilly@leeds.ac.uk

a.p.clegg@leeds.ac.uk

Corresponding author: Andrew Clegg (a.p.clegg@leeds.ac.uk)

Word count: 4,665 words (main manuscript)

For peer review only

Abstract

Introduction: The Community Ageing Research 75+ Study (CARE75+) is a longitudinal cohort study collecting an extensive range of health, social and economic data, with a focus on frailty, independence and quality of life in older age. CARE75+ is the first international experimental frailty research cohort designed using Trial within Cohort (TwiCs) methodology, to align applied epidemiological research with clinical trial evaluation of interventions to improve the health and wellbeing of older people living with frailty.

Methods and analysis: Prospective cohort study using a trial within cohort (TwiCs) design. One thousand community-dwelling older people (≥75years) will be recruited from UK general practices. Nursing home residents, those with an estimated life expectancy of three months or less and people receiving palliative care will be excluded. Data collection assessments will be face-to-face in the person's home at baseline, six months, 12 months, 24 month and 48 months, including assessments of frailty, cognition, mood, health-related quality of life, comorbidity, medications, resilience, loneliness, pain and self-efficacy. A modified protocol for follow-up by telephone or web-based will be offered at six months. Consent will be sought for data linkage and invitations to additional studies, including intervention studies using the TwiCs design. A blood sample bio-bank will be established for future basic science studies.

Ethics and dissemination: CARE75+ was approved by the NRES Committee Yorkshire & the Humber - Bradford Leeds 10th October 2014 (14/YH/1120). Formal written consent is sought if an individual is willing to participate and has capacity to provide informed consent. Consultee assent is sought if an individual lacks capacity.

Study results will be disseminated in peer-reviewed scientific journals and scientific conferences. Key study results will be summarised and disseminated to all study participants

For peer review only - http://bmjopen.bmj.com/site/about/guidelines.xhtml

via newsletters, local older people's publications and local engagement events. Results will be reported on a bespoke CARE75+ website.

Trial registration

ID ISRCTN16588124 (date of registration 26th February 2016 - retrospectively registered)

Strengths and limitations of this study

- CARE75+ is a prospective cohort study recruiting older people aged 75 and over, designed using Trial within Cohort (TwiCs) methods, collecting an extensive range of demographic, health and socioeconomic data at baseline, six, 12, 24 and 48 months.
- Our recruitment strategy, including home consent visits, home assessments, and use of researchers with community language skills, is designed to optimise the recruitment of older people across the frailty spectrum.
- CARE75+ will recruit participants from a variety of ethnic backgrounds and those with advanced frailty who are often underrepresented in research.
- Care home residents are not eligible for the study, aligned with the TwiCs design, meaning that findings cannot be generalised to this group of especially frail older people.
- CARE75+ is a cohort of high strategic relevance, which will help shape future UK and international health and research policy in ageing and frailty.

For peer review only

Introduction

Global ageing demographic projections indicate that there will be two billion people aged over 65 worldwide by 2050 [1, 2]. Frailty is an especially problematic expression of population ageing, with profound implications for planning and delivery of health and social care services globally. It is a condition characterised by loss of biological reserves, failure of homeostatic mechanisms and increased vulnerability to adverse outcomes following relatively minor stressor events [3, 4]. Thus, a mild infection, new medication, or minor surgery can result in a sudden, disproportionate change in health status or functional status for an older person with frailty, for example a change from independence to dependence, a fall, or development of delirium. Frailty is also associated with an increased risk of a range of adverse outcomes, including future disability, admission to hospital, long-term care residence and mortality [5].

To date, the healthcare response to frailty has been predominantly reactive and secondary care based. However, there is increasing recognition that frailty should be identified and managed as a long-term condition with preventative and proactive care models [6]. Furthermore, with the widespread introduction of robustly developed tools to detect frailty in primary care such as the electronic Frailty Index (eFI) based on routinely available primary care electronic health record (EHR) data in the UK [7], primary care teams can now more readily and reliably identify older people with frailty within their patient populations. These novel approaches are providing opportunities to develop and deliver services according to frailty status rather than chronological age.

Improved management of frailty requires an integrated approach spanning primary care, secondary care and social services that incorporates consideration of frailty transitions and health trajectories. Where possible, integrated care pathways should be developed and implemented based on suitably targeted, evidence-based interventions. Although

For peer review only - http://bmjopen.bmj.com/site/about/guidelines.xhtml

recruitment to ageing and frailty observational research studies has historically been relatively high [8, 9], recruitment rates to clinical trials of frailty interventions have frequently been low.

The Trial within Cohort (TwiCs) design [10] is an innovative research methodology that has the potential to enhance participation of older people with frailty in a range of studies including clinical trials, and to increase the capacity to conduct high quality frailty research [11]. The TwiCs design has several key features including the establishment of an observational cohort to both provide longitudinal data and function as a recruitment platform for multiple trials and other research studies. Each individual trial uses random selection of some (not all) participants from the cohort; intervention-centred information and consent is applied. The process aims to replicate the real world of routine health care by taking informed consent only from those randomised to receive an intervention, as the ongoing cohort study provides a natural control group.

Methods and analysis

Aim

Our aim is to establish a longitudinal cohort of older people to investigate frailty, disability and quality of life in older age and to act a recruitment platform for future studies (sub-studies) to enable the development and evaluation of interventions to improve outcomes for older people.

Patient and public involvement

We have established a Frailty Oversight Group (FOG) as a central component of the CARE75+ study. The FOG comprises a core reference group of four key individuals with links to local community organisations involved in the support of older people living with frailty, and a minority ethnic group advocate from the local authority. The FOG play a key

For peer review only - http://bmjopen.bmj.com/site/about/guidelines.xhtml

role in developing research questions for the cohort, including reviewing any proposed data analyses or nested studies.

The FOG had close involvement in developing and piloting the outcome assessment schedule for the study, highlighting the need to include measures that extend beyond traditional health domains into areas such as loneliness and resilience in later life. The FOG contributed to the development of all study materials, including invitation letters and participant information sheets, to ensure alignment with the needs of older people. Results are disseminated widely to participants, including through regular newsletters and an annual celebration event.

Design

A multi-site, community-based cohort study using a TwiCs design [10].

Inclusion criteria

Community dwelling older people aged ≥75 years.

Exclusion criteria

People with terminal cancer, life expectancy of three months or less and people in receipt of palliative care services will be excluded. Care home residents and people living at home who are bedbound will be excluded. However, we will attempt to follow-up people who transition to a care home during the course of the study.

Assessments

The CARE75+ assessment includes detailed information on the demographic, health and social circumstances of participants. An extensive range of measures are collected using validated instruments, including assessments of frailty, cognition, mood, health-related

For peer review only - http://bmjopen.bmj.com/site/about/guidelines.xhtml

quality of life, comorbidity, medications, resilience, loneliness and self-efficacy. The selected measures have been carefully chosen to ensure that CARE75+ includes measures with the necessary validity, reliability and responsiveness to enable both applied epidemiological investigation and randomised trial evaluation of future interventions to improve outcomes.

List of current assessments

- Demographic information (age, sex, ethnicity, marital status, living circumstances, housing type, education, previous occupation).
- Family networks and informal support (self-report).
- Resource use: GP, hospital and outpatient admissions. Use of aids and adaptations (self-report)
- Formal care (self-report).
- Smoking habits & alcohol consumption (self-report).
- Vision LogMar Vision test [12] (Thompson Software Solutions) [13].
- Hearing (the Whispered Voice test) [14].
- Sleep (self-report).
- Medication (prescribed) details (name, dose, frequency) will be collected from Primary Care Electronic Health Records (EHR). Non-prescribed medication will be self-reported.
- Cognitive function assessed using the Montreal Cognitive Assessment (MoCA) [15], a brief cognitive assessment instrument. The MoCA assesses different cognitive domains: attention and concentration; executive function; memory; language; conceptual thinking; calculations; and orientation. The total possible score is 30, with higher scores indicating better cognitive function, and a score of ≥26 considered normal.

For peer review only - http://bmjopen.bmj.com/site/about/guidelines.xhtml

- Comorbidities data, collected via the primary care electronic health record (EHR), and by self-report using the Katz comorbidity questionnaire [16]. This questionnaire asks questions on various health conditions requiring a ‘yes’ or ‘no’ response.
- General health and health related quality of life, using the RAND short-form 36-Item Health Survey (SF36) [17] which includes 36 questions spanning eight health domains: physical functioning; bodily pain; role limitations due to physical health problems; role limitations due to personal or emotional problems; general mental health; social functioning; energy/fatigue; and general health perceptions. It also includes a single item that provides an indication of perceived change in health. The SF36 enables calculation of Physical Component Summary (PCS) and Mental Component Summary (MCS) scores, and derivation of an overall health utility score, the short-form 6 dimension score (SF6D) suitable for use in economic evaluations [18].
- Health related quality of life using the EuroQol five dimension health questionnaire (five-level version) EQ5D-5L [19]. The EQ5D-5L five dimensions are: mobility, self-care, usual activities, pain/discomfort and anxiety/depression. Each dimension has 5 levels of severity: no problems, slight problems, moderate problems, severe problems and extreme problems. The scores for each of the five dimensions are combined in a five digit number representing health status that can be converted into a utility index (0 for dead, 1 for perfect health and negative values for states worse than death) for use in economic evaluations.
- Basic activities of daily living (ADL) using the Barthel index (BI) [20]. The BI assesses functional status on a 20 point scale by recording ability to complete ten basic activities of daily living; bathing, bladder function, bowel function, dressing, feeding, grooming, mobility, stairs, toilet use and transfers. Higher scores indicate greater independence.

For peer review only

For peer review only - http://bmjopen.bmj.com/site/about/guidelines.xhtml

- Instrumental ADL, measured using the Nottingham Extended Activities of Daily Living (NEADL) scale [21]. The NEADL includes questions on everyday activities in the domains of mobility, kitchen, domestic and leisure and is scored between 0 and 66, with higher scores indicating greater independence.
- Measures of frailty:
    - Research standard 60 item frailty index, based on the cumulative deficit model of frailty [22], and previously validated as part of the English Longitudinal Study of Ageing (ELSA) [23]. The frailty index score is calculated an equally weighted proportion of the number of deficits present in an individual relative to the total possible.
    - The phenotype model of frailty, based on the five physical characteristics as reported in the original Cardiovascular Health Study (slow walking speed, weight loss, exhaustion, weak grip strength, low energy expenditure) [3]. Slow walking speed is assessed by a timed three metre walk and results stratified by height and gender using values described in the original Cardiovascular Health Study, from which the phenotype model was derived [3]. Weight loss is determined by the following question. "In the last year, have you lost more than 10 pounds unintentionally?" Exhaustion is identified using the following questions: "How often in the last week do you feel that everything you did was an effort?" and "could not get going?". Responses are: rarely or none of the time (< 1 day) = 0; some or a little of the time (1 - 2 days) = 1; moderate amount of the time (3 - 4 days) = 2; most of the time = 3. If the participant answers "2" or "3" to either question they meet the criterion for exhaustion. Hand grip strength is assessed using a Jamar dynamometer and stratified using criteria from the Cardiovascular Health Study [3] with the mean of three attempts calculated for the dominant and non-dominant hand. Low activity is assessed using data obtained from the Physical Activity domain of the SF36

For peer review only - http://bmjopen.bmj.com/site/about/guidelines.xhtml

[17]. Those with no characteristics are identified as fit, one or two characteristics as pre-frail and three to five characteristics as frail.

- The seven category Clinical Frailty Scale (CFS) [22], which is a validated measure of frailty based on clinical descriptors and pictographs, designed for specialist and non-specialist use in routine clinical practice. The CFS is an ordinal measure, with scores ranging from one (fit) to seven (severe frailty).
- The Edmonton Frail Scale (EFS) [24], which is a validated frailty measure designed for specialist and non-specialist use that records information on nine frailty domains (cognition, general health, functional independence, social support, medication use, nutrition, mood, continence, functional performance). The EFS is scored out of a total of 17, with higher scores indicating increasing frailty.
- The electronic frailty index (eFI) score [7], based on the cumulative deficit model of frailty, including 36 variables recorded in the primary care EHR as part of routine care. The eFI score is calculated as an equally weighted proportion of the number of deficits present in an individual relative to the total possible. The eFI enables identification of frailty categories (fit, mild frailty, moderate frailty, severe frailty) and is obtained directly from the primary care EHR.

- Height weight and body composition: researcher assessment using bio-impedance scales (Marsden BFA-220P Body fat analyser). Weight loss is obtained by self-report at baseline and calculated from previously recorded weight data at follow-up time-points.
- Blood pressure (Life source auto inflation blood pressure monitor): sitting (three times), standing (once).
- Mobility, calculated using the timed-up-and-go-test (TUGT) [25]. The TUGT assesses a person's mobility and requires both static and dynamic balance. It measures the

For peer review only - http://bmjopen.bmj.com/site/about/guidelines.xhtml

time that a person takes to rise from a chair, walk three metres, turn around, walk back to the chair, and sit down. A person's usual walking aid is used if needed. People completing the test in less than 20 seconds tend to be independently mobile, able to get in and out of a chair without assistance and climb stairs. People completing the test in 20-29 seconds demonstrate greater variability in mobility, balance and functional ability. Completion of the TUGT in 30 seconds or more identifies people likely to require assistance with getting in and out of a chair, climbing stairs and leaving the house.

- Pain, measured using the Geriatric Pain Measure Short Form [26]. This questionnaire includes items of pain intensity (current and last 7 days), and dichotomous items on how pain is impacting on a person's mobility, ability to accomplish tasks and to sleep. Items are combined to derive an overall summary score.
- Loneliness recorded using the 11 item De Jong Gierveld Loneliness scale [27]. Sub-categories of social and emotional loneliness are calculated and a total score is derived enabling identification of categories: not lonely; moderately lonely; severely lonely; very severely lonely.
- Resilience, measured using the Brief Resilience scale (BRS) [28]. The six items in the BRS include five response options, enabling calculation of an overall score ranging from 1 to 6, with higher scores indicating greater resilience.
- Self-efficacy, measured using the General Self-Efficacy Scale [29]. This scale lists ten items with four response options enabling generation of a summary score ranging from 10 to 40, with higher scores indicating greater resilience.
- Low mood, assessed using the Geriatric Depression Scale Short-Form with a score of ≥5 indicating an abnormal low mood state [30].
- Self-reported falls.

For peer review only

For peer review only - http://bmjopen.bmj.com/site/about/guidelines.xhtml

- Full blood count (Leeds & Bradford sties only): haemoglobin and mean cell volume; red blood cell (RBC) count; mean cell haemoglobin concentration; mean cell haemoglobin; RBC distribution width, white blood cell count (including neutrophils, lymphocytes; monocytes; eosinophils; basophils); and platelets
- Frozen blood aliquots (Leeds & Bradford sites only) for future biochemical analysis, including:
    - Routine biochemistry and haematology: renal profile; liver profile; serum albumin; bone profile; glucose; glycosylated haemoglobin; lipid profile; uric acid; clotting.
    - Endocrine function: cortisol; thyroid function; IGF-1; DHEAS; testosterone; oestradiol; vitamin D; PTH; neuronal specific protein.
    - Immune function: highly sensitive CRP; inflammatory cytokines; rheumatoid factor; markers of immunosenescence.
    - Nutritional markers: vitamin A; vitamins B2, B6, B12; vitamin C; ferritin; folate; homocysteine.
    - Biomarkers of ageing: DNA repair capacity; telomere length; markers of oxidative stress.
    - Genetic markers: DNA; RNA; plasma.

The CARE75+ data dictionary is available as an appendix file (see Additional file 1).

Assessment schedule

Participants will be assessed at baseline, six, 12, 24 and 48 months. Face-to-face assessments will be conducted in the participant's home. The feasibility of a modified, telephone-based or web-based assessment protocol will be tested at the six month time point for participants who are willing and able to undertake assessments in the alternative formats.

For peer review only

For peer review only - http://bmjopen.bmj.com/site/about/guidelines.xhtml

The assessment schedule for CARE75+ (baseline, six, 12, 24 and 48 months) has been carefully designed to accelerate the frailty translational research pathway by aligning robust epidemiological investigation with the typical follow-up schedule for feasibility and definitive trials of interventions.

Sample size

The CARE75+ study will generate a comprehensive dataset for applied epidemiological research and will act as a recruitment platform for additional studies (sub-studies), including qualitative studies as well as randomised controlled trials (RCTs) using TwiCs methods. Therefore, the initial recruitment target is based on appropriate sample size calculations for pilot RCTs of interventions to inform the design of future definitive RCTs alongside applied epidemiological investigation of modifiable component of frailty.

Previous observational studies involving older people with frailty have identified that between 600 - 1000 participants are required for reliable estimates of the main effects [31]. Following an initial pilot phase involving 200 participants to test recruitment methods and gather data on rates of assent to participation in future trials, we plan to recruit 1000 participants over a four year period. Previous observational studies involving the oldest old have reported 18 month attrition rates of around 25% due to mortality and withdrawal of consent [8]. As our cohort will include older people with frailty who are at increased risk of adverse outcomes we plan to recruit a minimum of 250 participants per year thereafter, to maintain a legacy cohort for future clinical trials. Findings from the CARE75+ study will inform the design of a future definitive experimental frailty research cohort of sufficient size to nest a series of definitive intervention trials targeted at a range of potentially modifiable components of frailty, including people living with different frailty severity grades.

For peer review only - http://bmjopen.bmj.com/site/about/guidelines.xhtml

Recruitment

We will work with general practices to identify and recruit participants in primary care. Following initial piloting of recruitment methods in Bradford and Leeds, West Yorkshire, we will extend recruitment to other practices in England, using the skills and experience of staff within the National Institute for Health Research Clinical Research Networks (NIHR CRN).

Participant contact

Potential participants will be posted a study invitation pack containing a letter of invitation, a user-friendly participant information leaflet with photographs of the research staff involved in the home visits, and a supporting letter from their general practice. Potential participants who are not interested in participating in the study will be invited to contact their general practice to opt out. If potential participants do not opt out, contact details of eligible participants will be provided to the research team via a secure email system. The invitation letter will be followed up after two weeks with a telephone call from a researcher to discuss the study in more detail. If initial interest is expressed, the researcher and potential participant will arrange a home visit for an in-depth discussion of the study, where informed, written consent to participate will be sought.

The recruitment methods take into account the range of physical and cognitive challenges encountered by older people. Experience from previous cohort studies involving older people with frailty, disability and cognitive impairment has demonstrated that direct telephone calls or in-person visits are the only reliable methods of finding out whether potential participants are interested in participating, and may be preferred because they are seen as less of a burden [31]. Recruitment procedures will ensure that an older person with frailty receives all the necessary information to make an informed decision about participation. Procedures have been developed in close partnership with lay representatives through our Patient and Public Involvement Frailty Oversight Group [32], established as part of the National Institute

For peer review only

For peer review only - http://bmjopen.bmj.com/site/about/guidelines.xhtml

for Health Research Collaboration for Leadership in Applied Health Research and Care, Yorkshire and Humber (NIHR CLAHRC Yorkshire & Humber) programme.

Participant consent

Following initial telephone contact, researchers will visit participants who express an interest in participation and verbally explain the study in detail, including providing a comprehensive study information leaflet. Potential participants will be able to have an advocate, family member or friend present and will be offered 48 hours to reflect on the information before deciding to consent. For individuals whose first language is not English, a community language speaking researcher will be assigned where possible, or a suitable advocate identified.

Researchers will assess an individual's capacity to consent in accordance with the Mental Capacity Act (MCA) [33]. Formal written consent will be sought if an individual is willing to participate and has capacity to provide informed consent. The consent form will detail all processing and disclosure of the information collected including data analysis, data linkage, providing contact details to future researchers, and the storage and use of blood samples. Some components of the consent will be optional (for example taking and storing blood, consenting to be approached about other studies). Written consultee assent will be sought if individual participants do not have capacity to consent.

Data collection methods

We plan face-to-face data collection, but we will test the feasibility of telephone or web-based modified data collection procedure for participants who are willing, and able, at the six month time point.

For peer review only

For peer review only - http://bmjopen.bmj.com/site/about/guidelines.xhtml

Prescribed medications, comorbidity data and eFI scores will be obtained from general practice EHRs, extracted using standardised reporting templates developed for the SystmOne [34] and EMISWeb [35] primary care EHR systems.

All data will be collected using a bespoke electronic data capture application (EDCA), the CARE75+app developed and tested by Tigerteam Software Ltd[a]. Blood samples will be collected at baseline and 12 months from participants in the Bradford and Leeds sites.

Research staff training

Research staff will undertake a bespoke training programme, depending on skills and experience, including: the Mental Capacity Act [33]; research with older people; phlebotomy and safeguarding vulnerable adults. Additionally, staff will receive training in completion of the individual assessment measures and data entry into the Electronic Data Capture Application (EDCA).

Plans to promote participant retention and complete follow-up

We will seek broad and enduring consent for data linkage and use of collected data following withdrawal or death, aligned with Medical Research Council (MRC) guidelines for maximising the use of cohort data [36].

We will post newsletters to participants at least twice a year to provide study updates and encourage continued engagement. We will hold annual engagement events, where feasible to do so, and promote the study locally via affiliated newsletters (e.g. Age UK Voice magazine) and local forums.

For peer review only - http://bmjopen.bmj.com/site/about/guidelines.xhtml

Data entry, coding, security and storage

The EDCA will comprise two main components: a Data Collection Application (DCA) and Back Office System (BOS) containing personal identifiable information. The DCA will run on Microsoft Windows platform using an encrypted embedded database to temporarily store data. The BOS database will be on a Microsoft SQL server hosted at Bradford Teaching Hospitals NHS Foundation Trust (BTHFT). All data will be captured off-line in the community. Data will be uploaded regularly to ensure no identifiable data remains on the portable device for longer than 48 hours. Named researchers will have access to the individual details only whilst data collection takes place. A participant's details will only be released to one researcher at a time via the BOS management system. Access to modules and functions of both the DCA and BOS will governed by usernames, passwords and role specific access permissions, to maximise data security.

Remote site data (outside BTHFT) and the on-line completion forms (optional 6 month follow-up protocol) will be transferred to the BIHR-CARE database via the web application auecr.bradfordhospitals.nhs.uk hosted on the web server bhts-bihrweb. The site will be protected by SSL certificates, to encrypt the transfer of data over the internet. Access to the web application https://auecr.bradfordhospitals.nhs.uk on the server bhts-bihrweb will be restricted and protected by the Threat Management Gateway (TMG) software and SSL certificates. Remote site administrators and researchers will only have access to their own local participants.

Access to the BIHR-CARE database information will be based on role specific permissions. The chief investigator and project manager will have access to all data, at all levels for administration and governance purposes. Local site administrators will have access to local participant details. Researchers will have access to individual (site specific) case information only at the time of data collection. Researchers will have a maximum of three participants

For peer review only - http://bmjopen.bmj.com/site/about/guidelines.xhtml

available on portable devices (laptops) at any one time. Pathology laboratory staff will have access to blood sample data entry pages only. Statisticians and other members of the CARE75+ research team will only have access to pseudo anonymised i.e. those with unique identifiers for use in data linkage or anonymous data. Individual participants will be limited to access to a blank follow up questionnaire to complete and submit. All submitted data is final and data access is only available to the Super Administrator at BTHFT.

Data quality

Data quality will be enhanced by integral features of the data capture software, which will identify missing data and outlying values in real time. The software will automatically calculate the total scores for composite assessments. This will increase research efficiency and research data quality by reducing resource required for data cleansing, coding for analysis and reduce inputting errors.

Statistical methods

We plan interim data analyses after the completion of each stage i.e. baseline, 6, 12, 24 and 48 month follow-up of the study. We will assess frailty transitions using multivariate statistical methods. We will estimate health and social care resource use associated with frailty using economic modelling techniques.

We will conduct applied epidemiological investigation of the association between potentially modifiable components of frailty and outcomes, including: how pain modifies the association between frailty and disability; how resilience modifies the association between frailty and disability; and the association between frailty, mood and outcomes. We will assess frailty transitions using transition modelling. We will estimate health and social care resource use associated with frailty using economic modelling techniques.

For peer review only

For peer review only - http://bmjopen.bmj.com/site/about/guidelines.xhtml

Methods for any additional analyses (subgroup and adjusted analyses)

Data will be made available to external investigators upon request and reviewed by the CARE75+ Data Request Review Committee (DRRC), comprising the Chief Investigator, CARE75+ project manager, database manager, an independent member and independent lay representative from the Frailty Oversight Group [32].

The ethnic diversity of our planned recruitment sites will enable the investigation of ageing, frailty and disability in different cultural contexts.

Missing data

Methods for dealing with missing data will depend on the amount of missing data and patterns of missingness for individual variables as part of individual analyses. We will undertake sensitivity analysis to investigate the impact of missing data and we will explore the use of appropriate imputation methods.

Ethics and dissemination

This study was approved by the NRES Committee Yorkshire & the Humber - Bradford Leeds on the 10th October 2014 (14/YH/1120). CARE75+ is an observational study with low risk to participants. Cohort governance will be provided by the National Institute for Health Research Collaboration for Leadership in Applied Health Research and Care Yorkshire & Humber (NIHR CLAHRC YH) Frailty Theme [37] Operational Group comprised of the Theme Leads, Theme Manger, Project Managers and co-applicants. Independent scrutiny will be provided by the Frailty Oversight Group [32], which is comprised of lay members with networks into the wider community of older people in Bradford. Day to day monitoring, including data quality checks and validations will be the responsibility of a dedicated database manager.

For peer review only - http://bmjopen.bmj.com/site/about/guidelines.xhtml

Access to data

BTHFT will be the data controller for CARE75+. Data will be made available to external researchers in accordance with CARE75+ data sharing protocols following review of the CARE75+ data dictionary (supplementary file 1) and completion of the CARE75+ data request form (supplementary file 2), review by the DRRC and completion of a data sharing transfer agreement.

Ancillary and post-study care

We anticipate that some participants may have potentially unmet care needs and may wish to discuss these with the researcher. We will ensure that researchers are able to signpost participants to local statutory and voluntary organisations (e.g. Age UK), or request a GP referral for social services assessment so that appropriate plans can be made for ongoing care.

Safeguarding issues identified during the assessment visits will be reported to the Research Project Manager who will then take advice from the Adult Safeguarding Co-ordinator in the relevant local authorities.

Dissemination policy

Study results will be disseminated in peer-reviewed scientific journals and submitted for consideration at local, national and international scientific conferences. Key study results will be summarised and disseminated to all study participants via newsletters, local older people's publication (e.g. Voice magazine, Age UK) and local engagement events. Results will be reported on a bespoke CARE75+ website.

Research outputs using data from the CARE75+ study will be required to acknowledge the data source and funder using standardised wording. Additionally, studies involving

For peer review only - http://bmjopen.bmj.com/site/about/guidelines.xhtml

participants identified from the cohort (sub-studies) will be required to acknowledge the CARE75+ cohort in all reports. The full protocol and participant level dataset will be made available to not-for-profit investigators. Enquiries should be made to the CARE75+ Chief Investigator and will be reviewed by the DRRC.

Discussion

CARE75+ will use novel TwiCs methodology to align applied epidemiological research into ageing and frailty with clinical trials of interventions, potentially accelerating the translational research pathway in this important area.

We describe methods to recruit a cohort of older people and collect an extensive range of health, social and economic outcome data. We plan to collect a range of validated measurements of frailty in CARE75+, including the eFI, which has been made available to every general practice in England through a national implementation project, facilitating the rapid translation of research findings into clinical practice. Our recruitment strategy, including home consent visits, home assessments, and use of researchers with community language skills, is designed to optimise the recruitment of older people across the frailty spectrum and from a variety of ethnic backgrounds, including those with advanced frailty who are often underrepresented in research. Care home residents are not eligible for the study, aligned with the TwiCs design, meaning that findings cannot be generalised to this group of especially frail older people.

Our vision for CARE75+ is a cohort of high strategic relevance, which will help shape future UK and international health and research policy in ageing and frailty.

References

For peer review only - http://bmjopen.bmj.com/site/about/guidelines.xhtml

1. Kinsella KG, Phillips DR. Global aging: The challenge of success. Vol. 60. 2005: Population Reference Bureau Washington, DC.

2. United Nations. The World at Six Billion. 1999. www.un.org/esa/population/publications/sixbillion/sixbilpart1.pdf. Accessed 15 Feb 2018.

3. Fried LP, Tangen CM, Walston J, Newman AB, Hirsch C, Gottdiener J, Seeman T, Tracy, R, Kop WJ, Burke G, McBurnie MA. Frailty in older adults: evidence for a phenotype. J. Gerontol. A Biol. Sci. Med. Sci. 2001; 56: M146-M157.

4. Walston J, Hadley EC, Ferrucci L, Guralnik JM, Newman AB, Studenski SA, Ershler WB, Harris T, Fried LP. Research agenda for frailty in older adults: toward a better understanding of physiology and aetiology: summary from the American Geriatrics Society/National Institute on Aging Research Conference on Frailty in Older Adults. J Am Geriatr Soc. 2006; 54: 991-1001.

5. Clegg A, Young J, Iliffe S, Rikkert MO, Rockwood K. Frailty in elderly people. Lancet. 2013; 381; 752-62.

6. Harrison JK, Clegg A, Conroy SP, Young J. Managing frailty as a long-term condition. Age and Ageing: 2015; 44: 732- 5.

7. Clegg A, Bates C, Young J, Ryan R, Nichols L, Ann Teale, E, Mohammed MA, Parry J, Marshall T. Development and validation of an electronic frailty index using routine primary care electronic health record data. Age Ageing. 2016; 45: 353-60.

8. Davies K, Collerton JC, Jagger C, Bond J, Barker SA, Edwards J, Hughes J, Hunt JM, Robinson L. Engaging the oldest old in research: lessons from the Newcastle 85+ study. BMC Geriatr. 2010; 10: 64.

9. Bootsma-Van der Wiel A, Van Exel E, De Craen AJM, Gussekloo J, Lagaay AM, Knook DL, Westendorp RGJ. A high response is not essential to prevent selection bias: results from the Leiden 85-plus study. J Clin Epidemiol. 2002; 55: 1119-25.

10. Relton C, Torgerson D, O'Cathain A, Nicholl J. Rethinking pragmatic randomised controlled trials: introducing the cohort multiple randomised controlled trial design. BMJ. 2010; 340: c1066

For peer review only - http://bmjopen.bmj.com/site/about/guidelines.xhtml

11. Clegg A, Relton C, Young J, Witham M. Improving recruitment of older people to clinical trials: use of the cohort multiple randomised controlled trial design. Age Ageing. 2015; 44: 547-50.

12. Evans JR, Fletcher AE, Wormald RP, Ng ES, Stirling S, Smeeth L, Breeze E, Bulpitt CJ, Nunes M, Jones D, Tulloch A. Prevalence of visual impairment in people aged 75 years and older in Britain: results from the MRC trial of assessment and management of older people in the community. British Journal of Ophthalmology. 2002; 86: 795-800.

13. Electronic LogMar Vision Test, Thompson Software Soultions; http://www.thomson-software-solutions.com/test-chart-lite. Accessed 15 Feb 2018.

14. Pirozzo S, Papinczak T, Glasziou P. Whispered voice test for screening for hearing impairment in adults and children: systematic review. BMJ. 2003; 327: 967.

15. Nasreddine ZS, Phillips NA, Bédirian V, Charbonneau S, Whitehead V, Collin I, Cummings JL, Chertkow H. The Montreal Cognitive Assessment, MoCA: a brief screening tool for mild cognitive impairment. J Am Geriatr Soc. 2005; 53: 695-9.

16. Katz JN, Chang LC, Sangha O, Fossel AH, Bates DW. Can comorbidity be measured by questionnaire rather than medical record review? Med Care. 1996; 34: 73-84.

17. Hays RD, Sherbourne CD, Mazel RM. The rand 36-item health survey 1.0. Health economics. 1993; 2:217-27.

18. Brazier J, Roberts J, Deverill M. The estimation of a preference-based measure of health from the SF-36. Journal of Health Economics. 2002; 21:271-92.

19. EuroQoL. EQ-5D-5L. 24/01/2018]; Available from: https://euroqol.org/eq-5d-instruments/eq-5d-5l-about/. Accessed 15 Feb 2018.

20. Mahoney FI, Barthel DW. Functional Evaluation: The Barthel Index. Md State Med J, 1965; 14: 61-5.

21. Nouri F, Lincoln N. An extended activities of daily living scale for stroke patients. Clin Rehabil. 1987; 1: 301-5.

For peer review only - http://bmjopen.bmj.com/site/about/guidelines.xhtml

22. Rockwood K, Song X, MacKnight C, Bergman H, Hogan DB, McDowell I, Mitnitski A. A global clinical measure of fitness and frailty in elderly people. Canadian Medical Association Journal. 2005;173:489-95.

23. Marshall A, Nazroo J, Tampubolon G, Vanhoutte B. Cohort differences in the levels and trajectories of frailty among older people in England. J Epidemiol Community Health. 2015; 69:316-21.

24. Rolfson DB, Majumdar SR, Tsuyuki RT, Tahir A, Rockwood K. Validity and reliability of the Edmonton Frail Scale. Age Ageing. 2006; 35: 526-9.

25. Podsiadlo D, Richardson S. The timed "Up & Go": a test of basic functional mobility for frail elderly persons. J Am Geriatr Soc. 1991; 39:142-8.

26. Blozik E, Stuck AE, Niemann S, Ferrell BA, Harari D, Renteln-Kruse WV, Gillmann G, Beck JC, Clough-Gorr KM. Geriatric Pain Measure short form: development and initial evaluation. J Am Geriatr Soc. 2007; 55: 2045-50.

27. Gierveld JD, Van Tilburg T. The De Jong Gierveld short scales for emotional and social loneliness: tested on data from 7 countries in the UN generations and gender surveys. Eur J Ageing. 2010; 7:121-30.

28. Smith BW, Dalen J, Wiggins K, Tooley E, Christopher P, Bernard J. The brief resilience scale: assessing the ability to bounce back. Int J Behav Med. 2008; 15:194-200.

29. Schwarzer R, Jerusalem M. The general self-efficacy scale (GSE). Anxiety Stress Coping. 2010; 12: 329-45.

30. Yesavage JA, Sheikh JI. 9/Geriatric depression scale (GDS) recent evidence and development of a shorter version. Clin Gerontol. 1986; 5:165-73.

31. Collerton J, Barrass K, Bond J, Eccles M, Jagger C, James O, Martin-Ruiz C, Robinson L, von Zglinicki T, Kirkwood T. The Newcastle 85+ study: biological, clinical and psychosocial factors associated with healthy ageing: study protocol. BMC Geriatr. 2007; 7: 14.

32. Heaven A, Brown L, Foster M, Clegg A. Keeping it credible in cohort multiple Randomised Controlled Trials: the Community Ageing Research 75+ (CARE75+) study

For peer review only - http://bmjopen.bmj.com/site/about/guidelines.xhtml

model of patient and public involvement and engagement. Research involvement and engagement. 2016; 2: 30.

33. Department of Health, Mental Capacity Act. 2005, London, HMSO.

34. tpp. Systmone. https://www.tpp-uk.com/products/systmonline Accessed 15 Feb 2018.

35. emis health. EMIS web. https://www.emishealth.com/products/emis-web/?tab=primary-care. Accessed 15 Feb 2018.

36. MRC. Maximising the value of UK population cohorts. 2014 https://www.mrc.ac.uk/publications/browse/maximising-the-value-of-uk-population-cohorts/. Accessed 15 Feb 2018.

37. CLAHRC Yorkshire & Humber. Primary care-based management of frailty in older people. 2017 07/12/17]; Available from: http://clahrc-yh.nihr.ac.uk/our-themes/primary-care-based-management-of-frailty-in-older-people. Accessed 15 Feb 2018.

For peer review only

Author contributions

AH, LB, AC & JY provided major contributions to the manuscript. ET, RH, KS, GM, TY, VG, BH, CCG provided oversight and guidance to the study design. All authors contributed to the writing and approved the final manuscript.

Funding

This research was funded by the NIHR CLAHRC Yorkshire and Humber - www.clahrc-yh.nihr.ac.uk (study funding number IS-CLA-0113-10020) and supported by the NIHR CLAHRC South West Peninsula and West Midlands CLAHRC. The views and opinions expressed are those of the author(s), and not necessarily those of the NHS, the NIHR or the Department of Health and Social Care.

Acknowledgements

For peer review only - http://bmjopen.bmj.com/site/about/guidelines.xhtml

We are especially grateful to the substantial contributions of members of our Frailty Oversight Group: Anne Grice, Marilyn Foster, Chris McDermott, David Walker, Akhlak Rauf and Ernie Lloyd, who helped to finalise the protocol, test the assessment procedures and reviewed study materials. We would also like to thank the Newcastle 85+ study team for their invaluable advice and support in setting up CARE75+. Finally, we would like to acknowledge staff from the National Institute for Health Research Clinical Research Networks (NIHR CRNs) in North East & North Cumbria, South West Peninsula, West Midlands, and Yorkshire & Humber and all participating general practices for their support and enthusiasm in recruiting participants.

Competing interests

None.

Word count

4,495 words (main manuscript)

Additional Files

Additional file 1

Excel .xls

CAREDataDictionaryPub

Data collected during CARE75+ assessments, including all variable and value names and labels.

References

1. Heaven, A., et al., *Keeping it credible in cohort multiple Randomised Controlled Trials: the Community Ageing Research 75+ (CARE 75+) study model of patient and public involvement and engagement.* Res Involv Engagem, 2016. **2**: p. 30.

For peer review only

1. Data items are grouped in tabs
2. Please indicate which items you require using **CUT AND PASTE (plain text) TO POPULATE** the SampleRequestForm.
3. EVERY item must have a variable name to be extracted. **DO NOT** only state 'all'
3. Additions / ammendments to data requests will require the submission of a new SampleRequestForm with the additions/ammendments highlighted

NB:

Any variable with an associated ‘valid’ column has been checked for an acceptable level of missing data. Valid=1.

Missing data is recorded as follows:

- Not applicable will be recorded in the dataset as -95
- Unable will be recorded in the dataset as -96
- Unwilling will be recorded in the dataset as -97
- Unavailable information will be recorded in the dataset as -98
- All other missing blank fields will be -99

Required Field (please X)	Variable	Variable Label	Values	Value Label	comment
	participant_id	unique participant id for linkage			
	nhs_number				
	registration_date	date of upload to database			
	surname				
	forenames				
	title				
	address_l1				
	address_l2				
	address_l3				
	address_l4				
	address_l5				
	postcode				
	home_telephone				
	work_telephone				
	mobile_telephone				
	SixMonthFollowUpPreference	how the participant wo	1	face to face	
			2	on-line	
			3	telephone	
			9	no preference	
	EmailAddress		verabtim		

Required Field (please X)	Variable	Variable Label	Values	Value Labels	comment
	participantid	unique identification number	100000+		
	Site	unique site number	Null	Bradford Leeds	
	NHSNumber	NHS assigned number	verbatim		
	surname	participant surname	verbatim		
	forenames	participant forenames	verbatim		
	gp_code	participant's general practice	verbatim		
	status	study status of participant	0	Not contacted	
			1	Attempt contact again	
			2	Consent visit	
			99	OOS	
			100	Baseline stage, Visit {0}	
			101	Baseline stage, COMPLETE	
			200	6 month stage, Visit {0}	
			201	6 month stage, COMPLETE	
			300	12 month stage, Visit {0}	
			301	12 month stage, COMPLETE	
			400	24 month stage, Visit {0}	
			401	24 month stage, COMPLETE	
			500	48 month stage, Visit {0}	
			501	48 month stage, COMPLETE	
			600	Post 48 month stage, Visit {0}	
			601	Post 48 month stage, COMPLETE	
			999	OOS	
	StartOfStageDate	date and time that data is changed entered in nev	yyyy-mm-dd / hh/mm/ss		
	dob	participant's date of birth	yyyy/mm/dd		
	date_of_death	participant's date of death	yyyy/mm/dd		
	sex	participant's gender	1	male	
			2	female	
	ethnicity	participant's ethnic origin	1	white	
			2	mixed white /black caribbean	
			3	mixed white / asian	
			4	mixed white / black african	
			5	other mixed	
			6	black african	
			7	asian indian	
			8	asian bangladeshi	
			9	asian pakistani	
			10	other asian	
			11	black caribbean	
			12	other black	
			13	chinese	
			14	other	
	Otherethnicity		verbatim		
	MaritalStatus	participant's martial status	1	Single, that is never married	
			2	Married	
			3	Remarried	
			4	Separated but still legally married	
			5	Divorced	
			6	Widowed	
			97/98/99	missing	
	FutureStudies_ParticipantConsent	interest in taking part in future studies	1	Yes	
			2	No	
			97/98/99	missing	
			2	no	
			97/98/99	missing	
	otherStudies	are you currently participating in any other resear	1	yes	
			2	no	
			97/98/99	missing	
	otherStudiesYes	details of other studies	verbatim		

Required Field (please X)	Variable	Variable Label	Values	Value Label	comment
	HouseType	housing type	1	Bungalow	
			2	Semi-detached house	
			3	Detached house	
			4	Terraced house	
			5	Flat (which floor)	
			6	Sheltered housing (which floor)	
			7	Extra care housing	
			8	Care home (residential)	
			9	Care home (nursing)	
			97/98/99	Missing	
	Temp	temperature in c of the ro	verbatim		
	OtherRoomTemp	are all your rooms a simila	1	yes	
			2	no	
			97/98/99	missing	
	WhyNotRoomsSimilarTemp	reason for temperature di	verbatim		
	LivingCircumstances	participant's living circum	1	lives alone	
			2	lives with partner/spouse	
			3	lives with family	
			4	lives with a friend	
			5	lives with other residents of a care home	
			97/98/99	missing	
	OwnHome	Participant/husband/wife	1	Yes	
			2	No	
			97/98/99	missing	
	HowLongAtCurrentAddress	How long lived at current	Verbatim		
	GoOutAloneInDay	Go out alone in day	1	Yes	
			2	No	
			97/98/99	missing	
	GoOutAloneInNight	Go out alone in night	1	Yes	
			2	No	
			97/98/99	missing	
	HowSafeDoYouFeelDuringDay	How safe (would) feel wal	1	Very safe	NB this area=within 15 min walk
			2	Fairly safe	
			3	A bit unsafe	
			4	Very unsafe	
			97/98/99	missing	
	HowSafeDoYouFeelDuringNight	How safe (would) feel wal	1	Very safe	NB this area=within 15 min walk
			2	Fairly safe	
			3	A bit unsafe	
			4	Very unsafe	
			97/98/99	missing	
Yes value as follows as adjacent question					
1	ConcernsGettingOutNone	What concerns, if any, stop you getting out & about			
2	ConcernsGettingOutTraffic				
3	ConcernsGettingOutAntiSocialBeha		97/98/99	missing	
4	ConcernsGettingOutFalling				
5	ConcernsGettingOutCrime				
6	ConcernsGettingOutOther				
	ConcernsGettingOutOtherDetails		Verbatim		
	HowSafeDoYouFeelCrossingRoads	How safe feel crossing roa	1	Very safe	
			2	Fairly safe	
			3	A bit unsafe	
			4	Very unsafe	
			97/98/99	missing	
	HowWorriedAboutBeingVictimOfC	How worried about being	1	Very	
			2	Fairly	
			3	Not very	
			4	Not at all	
			97/98/99		

Required Field (please X)	Variable	Variable Label	Values	Value Label	comment
	ageEducation	how old were you when you finished full time continuous education '	number		
	PaidWork	what was the last paid work that you did?'	number		
	Qualifications	what was the highest educational qualification you attained?'	1	GCSE	
			2	HNS/HND	
			3	diploma	
			4	AS and A level	
			5	bachelor's degree	
			6	postgraduate	
			7	no qualifications	
			97/98/99	missing	
	VoluntaryWork	do you currently do any voluntary work?'	1	yes	
			2	no	
			97/98/99	missing	

Required Field (please X)	Variable	Variable Label	Values	Value Label	comment
	HaveBrothers	did/do you have any...	number		
	HaveSisters	did/do you have any...	97/98/99	missing	
	HaveSons	did/do you have any...			
	HaveDaughters	did/do you have any...			
	HaveGrandsons	did/do you have any...			
	HaveGranddaughters	did/do you have any...			
	BrotherFirstName	did/do you have any...	verbatim		
	SisterFirstName	did/do you have any...			
	SonFirstName	did/do you have any...			
	DaughterFirstName	did/do you have any...			
	GrandsonFirstName	did/do you have any...			
	GraddaughterFirstName	did/do you have any...			
	BrotherAliveorDead	did/do you have any...	1	alive	
	SisterAliveorDead	did/do you have any...	2	dead	
	SonAliveorDead	did/do you have any...	97/98/99	missing	
	DaughterAliveorDead	did/do you have any...			
	GrandsonAliveorDead	did/do you have any...			
	GranddaughterAliveorDead	did/do you have any...			

Required Field (please X)	Variable	Variable Label	Values	Value Label	comment
		InformalSupportLift	If you needed a lift to somewhere urgently, could you ask someone for help?'	1	Yes
		InformalSupportIll	If you wer ill in bed and needed help at home, could you ask someone for help?'	2	No
				97/98/99	Missing
		informal SupportCrisisNumberof PeopleforSupport	If you had a serious personal crisis, how many people could you turn to for comfort and support?'	number	
				97/98/99	Missing
		informalSupporPeopleLiveClose	How many of these people live within a 15-20 minute drive, if any?'	number	
				97/98/99	Missing
		InformalSupportBrother	Can you tellme the people to whom you could turn to for help (you can choose more than one)?'	1	yes
		InformalSupportSister		2	no
		InformalSupportDaughter		97/98/99	missing
		InformalSupportSon			
		InformalSupportGranddaughter			
		InformalSupportGrandson			
		InformalSupportFriend			
		InformalSupportNeighbour			
		InformalSupportOther		verbatim	
		informalSupportLastFourWeeks	approximately how many hours of informal suport have you had in the last four weeks? (unpaid)	number	
				97/98/99	Missing
		ServicesVisitWarden_ServicesVisitsFrequency	In the last 4 weeks, have you seen or had a visit from, or to, any of the following services? If so, how often?'	1	Several times a day
		ServicesVisitHomecare_ServicesVisitsFrequency		2	Once a day
		ServicesVisitNightattendentsitter_ServicesVisitsFrequency		3	One or more times a week
		ServicesVisitDaysitter_ServicesVisitsFrequency		4	Less than once a week
		ServicesVisitMealsProvision_ServicesVisitsFrequency		5	No contact
		ServicesVisitCommunityNurse_ServicesVisitsFrequency		6	Don't know
		ServicesVisitPhysiotherapist_ServicesVisitsFrequency		7	Not applicable
		ServicesVisitOccupationalTherapist_ServicesVisitsFrequency		97/98/99	Missing
		ServicesVisitChiropodist_ServicesVisitsFrequency			
		ServicesVisitSpeachTherapist_ServicesVisitsFrequency			
		ServicesVisitDietician_ServicesVisitsFrequency			
		ServicesVisitSocialWorker_ServicesVisitsFrequency			
		FormalSupportLastFourWeeks	Approximately how many hours of formal support have you had in the last four weeks?'	number	
				97/98/99	Missing
		GPVisit	In the last 4 weeks have you been to see or had a visit from a GP?'	1	Yes
		OutpatientClinic	In the last 4 weeks have you attended an outpatients clinic?'	2	No
				97/98/99	Missing
		GPVisitHowOften	number of visits	number	
		OutpatientClinicHowOften	number of visits	number	
				97/98/99	Missing
		AidAtHomeGrabRails	Do you use any of the following adi/adaptations to help you at home (>1)	1	yes
		AidAtHomeToiletSeat		2	yes
		AidAtHomeBathSeat		3	yes
		AidAtHomeReclinerChair		4	yes
		AidAtHomeBedRail		5	yes
		AidAtHomeBedRisers		6	yes
		AidAtHomeHoist		7	yes
		AidAtHomeHelpingHand		8	yes
		AidAtHomeKeySafe		9	yes
		AidAtHomePendantAlarm		10	yes
		AidAtHomeStairLift		11	yes
		AidAtHomeOutsideStep		12	yes
		AidAtHomeWheelchair		13	yes
				0	no
				97/98/99	Missing

Required Field (please X)	Variable	Variable Label	Values	Value Label comment
	Smoker	Have you ever smoked (this can include cigaretters, roll-ups, piopes or cigars)?		
			1	Yes
			2	No
			97/98/99	Missing
	SmokeRecently	Do you smoke at all nowadays'?		
			1	Yes
			2	No
			97/98/99	Missing
	HowManyCigarettes	How many cigarettes as day do y	number	
			97/98/99	Missing
	HowMnayRollUpsOrPipes	If you smoke, or have smoked, ro	number	
			97/98/99	Missing
	HowManyYears	Approximately how mnay years h	number	
			97/98/99	Missing
	CALC_CigarettePackYears	Lifetime exposure		
			number	
			97/98/99	Missing
	CALC_TobaccoPackYears	Lifetime exposure		
			number	
			97/98/99	Missing
	drinkAlcohol	Do you ever drink alcohol incuding drinks you brew at home?'		
			1	Yes
			2	No
			97/98/99	Missing
	AlcoholLast12Months	...how often have you had na alcoholoic drink of any kind during the last 12 months?'		
			1	almost every day
			2	five or 6 days a week
			3	three or four days a week
			4	once or twice a week
			5	once or twice a month
			6	once every couple of months
			7	once or twice a year
			8	not at all in the last 12 months
			9	unsure
			97/98/99	Missing
	AlcoholLast7Days	how many days out of the last 7 (	number	
			97/98/99	Missing
	smallGlassOfWine	Thinking about the last 7 days, h	number	
	standardGlassOfWine		97/98/99	Missing
	LargeGlassOfWine			
	lowerStrengthBeer			
	botlleOfBeer			
	canOfBeer			
	Alcopop			
	SingleSpirit			

Required Field (please X)	Variable	Variable Label	Values	Value Label	comment	
	HearingAid	Do you have a hearing aid?'				
			1	Yes		
			2	No		
			3	Don't know		
			97/98/99	Missing		
	HearingAidUsage	How often do you use a hearing aid?'				
			1	Always		
			2	Regularly		
			3	Only on special occasions		
			4	Never		
			97/98/99	Missing		
	HearingTest	Have you had a hearing test in the last year?'				
			1	Yes		
			2	No		
			3	Don't know		
			97/98/99	Missing		
	DifficultyHearing	Interviewer's assessment of participant's difficulty hearing so far				
			1	No difficulty		
			2	Some difficulty		
			3	Unable to hear at all		
			97/98/99	Missing		
	HearingInQuietRoom	Do you have difficultly hearing someo	1	Yes		
			2	No		
			3	Don't know		
			97/98/99	Missing		
	HearingInLoudRoom	Do you find it difficult to follow a conv	1	Yes		
			2	No		
			3	Don't know		
			97/98/99	Missing		
	WhisperTestResult	Whisper hearing test result	1	Pass		
			2	Fail		
			97/98/99	Missing		
	GlassesOrLenses	Do you use glasses/contact lenses?'	1	Yes		
			2	No		
			3	Don't know		
			97/98/99	Missing		
	GlassesOrLensesUsedFor	purpose of glasses/lenses	1	Distance		
			2	Reading		
			3	Both distance & reading		
			4	Don't know		
			97/98/99	Missing		
	EyesightTested	...eyesight tested by an optician in the	1	Yes		
			2	No		
			3	Don't know		
			97/98/99	Missing		
	RecognisingPeople	....difficulty recognisign a friend across	1	Yes		
			2	No		
			3	Don't know		
			97/98/99	Missing		
	ReadingNewsprint	...difficulty reading ordinary newsprint	1	Yes		
			2	No		
			3	Don't know		
			97/98/99	Missing		
	BlindorPartiallySighted	Registered blind or partially sighted	1	Yes		
			2	No		
			3	Don't know		
			97/98/99	Missing		
	LeftEye	(no pinholes) is the participant able to	1	Yes		
			2	No- form vision only		
			3	No- no vision		
			97/98/99	Missing		
	LeftEyeYesMarginLabel	logMAR margin of the last line read co	number			
			97/98/99	Missing		
	LeftEyeYesNumLettersMissed	total number of letters missed or incor	number			
			97/98/99			
	RightEye	(no pinholes) is the participant able to	1	Yes		
			2	No- form vision only		
			3	No- no vision		
			97/98/99	Missing		
	RightEyeYesMarginLabel	logMAR margin of the last line read co	number			
			97/98/99	Missing		
	RightEyeYesNumLettersMissed	total number of letters missed or incor	number			
			97/98/99			
	LeftEyePinholes	(with pinholes) is the participant able	1	Yes		
			2	No- form vision only		
			3	No- no vision		
			97/98/99	Missing		
	LeftEyePinholesYesMarginLabel	logMAR margin of the last line read co	number			
			97/98/99	Missing		
	LeftEyePinholesYesNumLettersMissed	total number of letters missed or incor	number			
			97/98/99			
	RightEyePinholes	(with pinholes) is the participant able	1	Yes		
			2	No- form vision only		
			3	No- no vision		
			97/98/99	Missing		
	RightEyePinholesYesMarginLabel	logMAR margin of the last line read co	number			
			97/98/99	Missing		
	RightEyePinholesYesNumLettersMissed	total number of letters missed or incor	number			
			97/98/99			
	CALC_LogMARLeftEye	no pinhole score	number			
			97/98/99			
	CALC_LogMARRightEye	no pinhole score	number			
			97/98/99			
	CALC_LogMARLeftEyePinholes	with pinholes score	number			
			97/98/99			
	CALC_LogMARRightEyePinholes	with pinholes	number			
			97/98/99			

Required Field (please X)	Variable	Variable Label	Values	Value Label	comment
	SF-36HealthSurvey	In general how would you say your health is?'	1	Excellent	
			2	Very Good	
			3	Good	
			4	Fair	
			5	Poor	
			97/98/99	Missing	
	healthComparedToAYearAgo	Compared to one year ago, how would your rate your health in general now?	1	Much better now than one year ago	
			2	Somewhat better now than one year ago	
			3	About the same as one year ago	
			4	Somewhat worse now than one year ago	
			5	Much worse now than one year ago	
			97/98/99	Missing	
	VigorousActivities_SF36Activities	...does you health now limit you in these activities? If so how much?'	1	Limited a lot	
	ModerateActivities_SF36Activities		2	Limited a little	
	LiftingOrCarryingActivities_SF36Activities		3	Not limited at all	
	ClimbingSeveralStairsActivities_SF36Activities		97/98/99	Missing	
	ClimbingStairsActivities_SF36Activities				
	BendingKneelingStoopingActivities_SF36Activities				
	WalkingMoreThanAMile_SF36Activities				
	WalkingSeveralBlocksActivities_SF36Activities				
	WalkingOneBlockActivities_SF36Activities				
	BathingOrDressingYourself_SF36Activities				
	HealthIssuesReducedTimeSpentOnWork	During the past 4 weeks, have you had any of the following problems with your work	1	Yes	
	HealthIssuesAccomplishedLess		2	No	
	HealthIssuesLimitedOtherActivities		97/98/99	Missing	
	HealthIssuesDifficultiesPerformingWork				
	EmotionalIssuesReducedTimeSpentOnWork	During the past 4 weeks, have you had any of the following problems with your work	1	Yes	
	EmotionalIssuesAccomplishedLess		2	No	
	EmotionalIssuesDidntDoWorkAsCarefully		97/98/99	Missing	
	HealthInterferedActivities	During the past 4 weeks, to what extent has your physical health or emotional proble	1	Not at all	
			2	Slightly	
			3	Moderately	
			4	Quite a bit	
			5	Extremely	
			97/98/99	Missing	
	BodilyPain	How much bodily pain have you had during the past 4 weeks?'	1	None	
			2	Very mild	
			3	Mild	
			4	Moderate	
			5	Severe	
			6	Very severe	
			97/98/99	Missing	
	PainInterfereWithWork	During the past 4 weeks, how much did pain interfere with your normal work (includi	1	Not at all	
			2	A little bit	
			3	Moderately	
			4	Quite a bit	
			5	Extremely	
			97/98/99	Missing	
	FeelFullOfPep_FeelingResponses	How much time during the past 4 weeks have/did you ...?'	1	All of the Time	
	FeelNervous_FeelingResponses		2	Most of the Time	
	FeelDown_FeelingResponses		3	A Good Bit of the Time	
	FeltCalm_FeelingResponses		4	Some of the Time	
	ALotOfEnergy_FeelingResponses		5	A Little of the Time	
	FeltDownhearted_FeelingResponses		6	None of the Time	
	FeelWornOut_FeelingResponses		97/98/99	Missing	
	BeenHappy_FeelingResponses				
	FeelTired_FeelingResponses				
	HowOftenHealthInterferedActivities	During the past 4 weeks, how much of the time has your physical health or emotional	1	All of the time	
			2	Most of the time	
			3	Some of the time	
			4	A little of the time	
			5	None of the time	
			97/98/99	Missing	
	SickEasier_HealthExpectation	How True or False is each of the following statements for you?'	1	Definitely True	
	HealthyasAnybody_HealthExpectation		2	Mostly True	
	HealthToGetWorse_HealthExpectation		3	Don't Know	
	HealthIsExcellent_HealthExpectation		4	Mostly False	
			5	Definitely False	
			97/98/99	Missing	
	CALC_AvgSF36PhysicalFunctioning	total SF36 score for each domain	number		
	CALC_AvgSF36RoleLimitationPhysicalHealth				
	CALC_AvgSF36RoleLimitationEmotionalProblems				
	CALC_AvgSF36EnergyOrFatigue				
	CALC_AvgSF36EmotionalWellbeing				
	CALC_AvgSF36SocialFunctioning				
	CALC_AvgSF36Pain				
	CALC_AvgSF36GeneralHealth				
	CALC_AvgSF36HealthChange				

Required Field (please X)	Variable	Variable Label	Values	Value Label	comment
	MoCAlternatingTrailMaking	number letter sequencing	1	correct	
	MoCAVisuoconsturctinalCube	completion of 3D cube	2	incorrect	
	MoCAVisuoconsturctinalContour	drawing of clock face outline	97/98/99	Missing	
	MoCAVisuoconsturctinalHands	placement of clock hands			
	MoCAVisuoconsturctinalNumbers	placement of clock numbers			
	MocANamingLion		1	correct	
	MocANamingRhino		2	incorrect	
	MocANamingCamel		97/98/99	Missing	
	MoCADigitsBackward	repetition of digits forward	1	correct	
	MoCADigitsForward	repetition of digits backwards	2	incorrect	
			97/98/99	Missing	
	MoCAVigalence	recognition of 'A' in letter sequence	1	0-1 error	
			2	>1 error	
			97/98/99	Missing	
	MoCASerial	number of correct subtractions of 7 starting	0	correct subtractions	
			1		
			2		
			3		
			4		
			5		
			97/98/99	Missing	
	MoCASentenceRepetitionOne	repeat sentence	1	correct	
	MoCASentenceRepetitionTwo	repeat sentence	2	incorrect	
			97/98/99	Missing	
	MoCAVerbalFluency	tell me as many words as you can beginning	number		
			97/98/99	Missing	
	MoCAAbstractionOne	what is the likeness of train and bicycle?	1	correct	
	MoCAAbstractionTwo	what is the likeness of ruler and watch	2	incorrect	
			97/98/99	Missing	
	MoCADelayedRecallFace	recall of words read earlier	1	recalled	
	MoCADelayedRecallVelvet		2	not recalled	
	MoCADelayedRecallChurch		3	incorrect	
	MoCADelayedRecallDaisy		97/98/99	Missing	
	MoCADelayedRecallRed				
	MoCAOrientationDate	orientation to time and place	1	correct	
	MoCAOrientationMonth		2	incorrect	
	MoCAOrientationYear		97/98/99	Missing	
	MoCAOrientationDay				
	MoCAOrientationPlace				
	MoCAOrientationCity				
	MoCAOrientationEducation	years of formal education	1	<13	
			2	>=13	
			97/98/99	Missing	
	CALC_MoCA	score out of 30 >=26 'normal'	0-30		
			97/98/99	Missing	

Required Field (please X)	Variable	Variable Label	Values	Value Label comment
	MyocardialInfarction	Have you ever had a heart attack	1	yes
	CongestiveHeartFailure	Have you ever been treated for heart failure?	2	no
	PeripheralVascularDisease	Have you had an operation to unclog or bypass the art	3	not sure
	CerebrovascularAccident	Have you had a stroke, cerebrovascular accident, bloo	97/98/99	Missing
	Hemiplegia	Do you have difficulty moving an arm or leg as a result of the stroke or cerebrovascular accident?		
	Asthma	Do you have asthma?		
	AsthmaMedicines	Do you take medications for your asthma?'	2	no
			1	yes, only with flare ups of my asthma
			3	yes, I take medicine regularly even when I'm not having a flare up
			97/98/99	Missing
	emphysema	Do you have emphysema, chronic bronchitis, or chron	1	yes
			2	no
			3	not sure
			97/98/99	Missing
	emphysemaMedicines	Do you take medicines for your lung disease?'	2	no
			1	yes, only with flare ups
			3	yes, I take medicine regularly even when I'm not having a flare up
			97/98/99	Missing
	UlcerDisease	Do you have stomach ulcers or peptic ulcer disease diagnosed by endoscopy or upper gi or barium swallow study?		
	HasDiabetes	Do you have diabetes?	1	Yes, treated by modifying my diet
			2	No
			3	Yes, treated by medications taken by mouth
			4	Yes, treated by insulin
			97/98/99	Missing
	DiabetesCausedProblems	Has the diabetes caused any of the following problems	1	Problems with kidneys
			2	Problems with eyes, treated by an ophthalmologist
			3	unsure
			97/98/99	Missing
	ProblemsWithKidneys	Have you ever had problems with your kidneys?'	1	Poor kidney function (blood tests show high creatinine)
			2	No
			3	Have used hemodialysis or peritoneal dialysis
			4	Have received kidney transplantation
			97/98/99	Missing
	ConnectiveTissueDisese	Do you have Rheumatoid Arthritis?	1	Yes
			2	No
			3	Not sure
			97/98/99	Missing
	ConnectiveTissueDiseaseMedications	Do you take medications for it regularly?'	1	Yes
			2	No
			3	Not sure
			97/98/99	Missing
	ConnectiveTissueDiseaseType	do you have....?'	1	Lupua
			2	Polymyalgia rheumatica
			3	neither lupus nor polymalgia
			4	not sure
			97/98/99	Missing
	Dementia	Do you have any of the following conditions..?	1	Yes
	Cirrhosis		2	No
	Leukemia		3	Not sure
	Lymphoma		97/98/99	Missing
	Aids			
	Cancer			
		Has the cancer spread or metastasized to other parts c	1	Yes
	CancerSpread		2	No
			3	Not sure
			97/98/99	Missing
	CALC_KatzCormbidity	total score plus age based on the Charlson index scorii	number	
	Hypertension	Does the participant's GP record identify...	1	Yes
	1b. Any atherosclerotic disease		2	No
	1c. Ischaemic heart disease		3	Not sure
	Any atherosclerotic disease		2	No
	Ischaemic heart disease		97/98/99	Missing
	Cerebrovascular disease			
	Peripheral vascular disease			
	Heart failure			
	Atrial fibrillation			
	Atrial flutter			
	Osteoarthritis			
	Cervical or lumbar spondylosis			
	Rheumatoid arthritis			
	Other arthritis (specified)			
	Arthritis (type not specified)			
	Joint replacement			
	Cataract			
	Cataract surgery			
	Age related macular degeneration			
	Glaucoma Diabetic eye disease			
	Registered blind			
	Registered partially sighted			
	Any cancer			
	Any cancer, excluding nonmelanoma skin ca			
	Any cancer <5 years since diagnosis			
	Chronic obstructive pulmonary disease (COP			
	Asthma			
	Other respiratory disease			
	Diabetes mellitus			
	Hypothyroidism			
	Hyperthyroidism			
	Dementia			
	Parkinson's disease			
	Anxiety			
	Depression			
	Anaemia: WHO criteria for haemoglobin con			
	Anaemia: Joosten's criterion for haemoglobi			
	Osteoporosis			
	Fractured hip, wrist or backbone			
	Renal function using modification of diet in r		1	Normal/mildly reduced
			2	Moderately reduced (stage 3††)
			3	Severely reduced (Stage 4††)
			4	Very severely reduced (stage 5††)
			97/98/99	Missing
	Calculate total number of diseases	Total number of diseases from GP record (excluding os	Number	
			97/98/99	Missing

Required Field (please X)	Variable	Variable Label	Values	Value Label	comment
	FallsInLast12Months	in the last 12 months have you had a fall	1	Yes	
			2	no	
			3	not sure	
			97/98/99	missing	
	HowManyFalls	How mnay time s have you fallen in the l	number		
			97/98/99	missing	
	FallsFractures	...have you broken any bones/had and fra	1	Yes	
			2	no	
			3	not sure	
			97/98/99	missing	
	FallsBrokenBones	How many times has a fall resulted in a b	number		
			97/98/99	missing	
	FallsAccidentEmergency	...did you go to Aand E follwing a fall?	1	Yes	
			2	no	
			3	not sure	
			97/98/99	missing	
	FallsGetAandE	How many times did you attend A&E	number		
			97/98/99	missing	
	FallsStayOvernight	...were you admitted to a hospital followi	1	Yes	
			2	no	
			3	not sure	
			97/98/99	missing	
	FallsGetAdmited	How many times were you admitted to a	number		
			97/98/99	missing	
	FallsSeenGP	Have you (or your carer) ever seen your (	1	Yes	
			2	no	
			3	not sure	
			97/98/99	missing	
	FallsSpecialist	Have you ever seen a falls specialist?'	1	Yes	
			2	no	
			3	not sure	
			97/98/99	missing	
	LossOfConfidence	Have your falls caused any of the followi	1	Yes	
	WorryAboutFalling		2	no	
	GoingOutLessOften		3	not sure	
	IncreaseCareReceive		97/98/99	missing	

Required Field (please X)	Variable	Variable Label	Values	Value Label	comment
	TookPresMed	Number of prescribed medications	number		
			97/98/99	Missing	
	Medication*_MedicationName	Name of prescribed medication 1-30	verbatim		
			97/98/99	Missing	
	Medication*_Dosage	Dosage of prescribed medicationsverbatim	number		
			97/98/99	Missing	
	Medication*_Frequency	Frequency of prescribed medication	number		
			97/98/99	Missing	
	**TookNonPresMed**	Number of non-prescribed medicines, vitamin or mineral supplement	number		
			97/98/99	Missing	
	**NonPresMedication*_NonPresMedicationName**	Name of non-prescribed medication 1-30	verbatim		
			97/98/99	Missing	
	**NonPresMedication*_NonPresMedicationDosage**	Dosage of non-prescribed medicationsverbatim	number		
			97/98/99	Missing	
	**NonPresMedication*_NonPresMedicationFrequency**	Frequency of non-prescribed medication	number		
			97/98/99	Missing	
	**COUNT_PrescribedMedication**		number		
	**COUNT_NonPrescribedMedication**		number		

Required Field (please X)	Variable	Variable Label	Values	Value Label	comment
	WalkAroundOutside_NEADLPartOne	...what have you *actually*done in the l	0	not at all	
	ClimbStairs_NEADLPartOne		1	with help	
	GetInAndOutOfCar_NEADLPartOne		2	on your own with difficulty	
	WalkOverUnevenGround_NEADLPartOne		3	on your own	
	CossRoads_NEADLPartOne		97/98/99	Missing	
	TravelOnPublicTransport_NEADLPartOne				
	ManageToFeedYourself_NEADLPartOne				
	MakeYourselfAHotDrink_NEADLPartOne				
	TakeHotDrinksFromOneDrinksToAnother_NEADLPartOne				
	DoTheWashingUp_NEADLPartOne				
	MakeYourselfAHotSnack_NEADLPartOne				
	ManageOwnMoney_NEADLPartTwo	...what have you *actually*done in the l	0	no	
	WashItemsOfClothing_NEADLPartTwo		1	with help	
	DoOwnHousework_NEADLPartTwo		2	on your own with difficulty	
	DoOwnShopping_NEADLPartTwo		3	on your own	
	DoFullClothesWash_NEADLPartTwo		97/98/99	Missing	
	ReadNewspapersOrBooks_NEADLPartTwo				
	UseTelephone_NEADLPartTwo				
	WriteLetters_NEADLPartTwo				
	GoOutSocially_NEADLPartTwo				
	ManageGarden_NEADLPartTwo				
	Drive_NEADLPartTwo				
	CALC_NEADL	Total NEADL. Higher score = more ind	number 0-66		
			97/98/99	Missing	
	Feeding	Do you have any difficulty with the fol	0	uanble	
			1	needs help cutting, spreading butter or requires modified diet	
			2	independent	
			97/98/99	Missing	
	Bathing		0	dependent	
			1	independent (or in shower)	
			97/98/99	Missing	
	Grooming		0	needs help with personal care	
			1	independent face/hair/teeth/shaving	
			97/98/99	Missing	
	Dressing		0	dependent	
			1	needs help but can do about half unaided	
			2	independent including buttons and zips	
			97/98/99	Missing	
	Bowels		0	incontinent or needs enemas	
			1	occasional accident	
			2	continent	
			97/98/99	Missing	
	Bladder		0	unable (or catherterised and unable to manage alone)	
			1	occasional accident	
			2	continent	
			97/98/99	Missing	
	ToiletUse		0	dependent	
			1	needs some help but can so somethings alone	
			2	independent (on/off an wiping)	
			97/98/99	Missing	
	Transfers		0	unable, no sitting balance	
			1	major help (one or two people physical) can sit up	
			2	minor help (verbal or physical)	
			3	independent	
			97/98/99	Missing	
	Mobility		0	immobile or <50 yards	
			1	wheelchair independent including corners >50 yards	
			2	walks with the help of one person (verbal or physical) >50 yards	
			3	independent but may use any walking aide >50 yards	
			97/98/99	Missing	
	Stairs		0	unable	
			1	needs help (verbal physical, carrying aid)	
			2	independent	
			97/98/99	Missing	
	CALC_Barthel_Index	Total BARTHEL. Higher score means m	number 0-20		
			97/98/99	Missing	

Required Field (please X)	Variable	Variable Label	Values	Value Label	comment
	HeightWeighttimeofDay	time measurements are taken	hh.mm		
	Demispan	from middel of collar bone to index finger	number cm		
	DemispanHeight	Calculation of height from demispan	number cm		
	Height		number cm		
	Weight		number kg		
	BodyFatPercentage		number %		
	FatMass		number kg		
	FatFreeMass		number kg		
	BodyWaterPercentage		number %		
	MuscleMass		number kg		
	BoneMass		number kg		
	CALC_DemiSpanHeight				
	CALC_BMI	mass (kg) /demi span height (m)2	number		
	WeightLoss	In the last year, have you lost more than 10lb[illegible]	1	yes	
			2	no	
			3	not sure	
			97/98/99	Missing	
	SittingBloodPresssure		yes	1	
			no	2	
			97/98/99	Missing	
	ReasonNoSittingBloodPressure		verbatim		
			1	Participant refused	
			3	Cuff the wrong size	
			4	Monitor malfunction	
			5	StandingBloodPressureTaken	
	SittingSystolic	BP reading	number mm Hg		
	Sitting Diastolic	BP reading	97/98/99	Missing	
	SittingPulse	pulse			
	StandingBloodPresssure		yes	1	
			no	2	
			97/98/99	Missing	
	ReasonNoStandingBloodPressure		verbatim		
	StandingSystolic	BP reading	number mm Hg		
	Standing Diastolic	BP reading	97/98/99	Missing	
	StandingPulse	pulse			
	DominantHand	Which is the dominant hand?	1	right	
			2	left	
	DominantHandFirstAttempt	grip strength in dominant hand	number kPA		
	DominantHandSecondAttempt				
	DominantHandThirdAttempt				
	NondominantHandFirstAttempt	grip strength in passive hand	number kPA		
	NondominantHandSecondAttempt				
	NondominantHandThirdAttempt				
	CALC_DominantMeanGripStrength	mean grip strength dominant hand	number kPA		
	CAL_NonDominantMeanGripStrength	mean grip strength passive hand			

Required Field (please X)	Variable	Variable Label	Values	Value Label	comment	
	WalkTime	over a 3 metre distance	number seconds			
			97/98/99	Missing		
	ExhaustionPartA	How often in the last week did you feel 'everything I did was an effort?'	0	rarely or none of the time		
			1	some or a little of the time		
			2	moderate amount of the time		
			3	most of the time		
			97/98/99	Missing		
	ExhaustionPartB	How often in the last week did you feel 'I could not get going'?'	0	rarely or none of the time		
			1	some or a little of the time		
			2	moderate amount of the time		
			3	most of the time		
			97/98/99	Missing		
	TUGTSkipped	was the TUGT missed out?	1	yes		
			2	no		
	TUGNotDoneReason	reason for skipping TUGT	verbatim			
	chairUsed		1	yes		
			2	no		
	ChairHeight	height of the chair used to stand up from	number mm			
	AbleToGetUpFromChair		1	yes		
			2	no		
			97/98/99	Missing		
	TimeToGetUp	time taken to complete in seconds	number secs.			
			97/98/99	Missing		
	**WalkingAidUsed**	walking aid used to complete the TUGT	1	Independent		
			2	1x walking stick		
			3	2x walking sticks		
			4	Walking Zimmer frame		
			5	Wheeled Zimmer frame		
			6	3-wheeled walker		
			7	4-wheeled walker		
			8	Kitchen trolley		
			97/98/99	Missing		

Required Field (please X)	Variable	Variable Label	Values	Value Label	comment
	ClinicalFrailtyResearcher	Researcher frailty assessment based on observatic	1	very fit; robust, active, energetic, well motivated and fit. These people commonly exercise regularlry and are in the most fit group for their age	
			2	well; without active disease, but less fit htan people in category 1	
			3	well; with terated comorbid disease. Disease symptoms are controlled compared with those in category 4	
			4	apparently vulnerable; although not frankly dependent, these people commonly complain of beign 'slowed up' or have disease symptoms	
			5	mildly frail; with limited dependence on others for instrumental activities of daily living	
			6	moderately frail; help is needed with both instrumental and non-instrumental activities of daily living	
			7	severely frail; completely dependent on others for the activities of daily living or terminally ill	
			99	missing	
	**ClinicalFrailtyIsClinicianPresent**	Is a registrar present to complete 2nd part of the a	1	yes	
			2	no	
	**ClinicalFrailtyClinican**	Clinician frailty assessment based on observation	1	very fit; robust, active, energetic, well motivated and fit. These people commonly exercise regularlry and are in the most fit group for their age	
			2	well; without active disease, but less fit htan people in category 1	
			3	well; with terated comorbid disease. Disease symptoms are controlled compared with those in category 4	
			4	apparently vulnerable; although not frankly dependent, these people commonly complain of beign 'slowed up' or have disease symptoms	
			5	mildly frail; with limited dependence on others for instrumental activities of daily living	
			6	moderately frail; help is needed with both instrumental and non-instrumental activities of daily living	
			7	severely frail; completely dependent on others for the activities of daily living or terminally ill	
			99	missing	
	CALC_FriedTotalScore	Fried frailty score	0	not frail	
			1	pre frail	
			2	pre frail	
			3	mild frailty	
			4	moderate frailty	
			5	severe frailty	
			97/98/99	missing	
	PenFromHand	Can you take this oen from my hand if I hold it her	1	yes	
			2	no	
			97/98/99	missing	
	BustrainTimetable	Are you able to use a bus or train timetable?'	1	yes	
			2	no	
			97/98/99	missing	
	SitUprightforTwoHours	Are you able to sit upright in a chair for two hours	1	yes	
			2	no	
	CoinFromTable	Are you able to pick up a 10p coin from a table?'	1	yes	
			2	no	
			97/98/99	missing	
	EFSCognition	draw 10 past 11 on clock face	0	no errors	
			1	minor spacing errors	
			2	other errors	
			97/98/99	missing	
	EFSAdmitted	How many hospital admissions in the past year	0	0	
			1	1 or 2	
			2	>2	
			97/98/99	missing	
	EFSSocialSupport	can you count on someone?	0	always	
			1	sometimes	
			2	never	
			97/98/99	missing	
	EFSForgetMedication	At times do you forget to take your prescription m	0	no	
			1	yes	
			97/98/99	missing	
	EFSNutrition	Have you lost weight and clothes become loose?	0	no	
			1	yes	
			97/98/99	missing	
	EFSMood	Do you often feel sad or depressed?'	0	no	
			1	yes	
			97/98/99	missing	
	EFSContinence	Do you have a problem losing control of urine whe	0	no	
			1	yes	
			97/98/99	missing	
	CALC_EdmontonFrailScale	total score out of 17	0-5	not frail	
			6-7	vulnerable	
			8-9	mild frailty	
			10-11	moderate frailty	
			12-17	severe frailty	
			97/98/99	missing	
	EFIGPScore	data extracted from GP summary records			
	Fit (eFI score 0 - 0.12)	: People who have no or few long-term conditions that are usually well controlled. This group would mainly be independent in day to day living activities.			
	Mild frailty (eFI score 0.13 – 0.24)	: People who are slowing up in older age and may need help with personal activities of daily living such as finances, shopping, transportation.			
	Moderate Frailty (eFI score 0.25 – 0.36)	: People who have difficulties with outdoor activities and may have mobility problems or require help with activites such as washing and dressing.			
	Severe Frailty (eFI score > 0.36)	: People who are often dependent for personal cares and have a range of long-term conditions/multimorbidity. Some of this group may be medically stable but others can be unstable and at risk of dying within 6 - 12 months			
	99	missing			
	CALC_ELSAFrailtyIndex				
	0-10	very fit			
	11-14	well			
	15-24	vulnerable			
	>=25	frail			
	missing 99				
	http://www.elsa-project.ac.uk/publicationDetails/id/7167				

Required Field (please X)	Variable	Variable La	Values	Value Label	comment		
	Sleep	Have you sl	1	Yes			
			2	No			
			3	Not sure			
			97/98/99	Missing			
	HealthRelatedMobility	EQ5D	1	no problems walking about			
			2	slight problems			
			3	moderate problems			
			4	severe problems			
			5	unable to walk			
			97/98/99	Missing			
	HealthRelatedSelfCare		1	no problems washing or dressing			
			2	slight problems			
			3	moderate problems			
			4	severe problems			
			5	unable to wash or dress			
			97/98/99	Missing			
	HealthRelatedUsualActivities		1	no problems doing usual activities			
			2	slight problems			
			3	moderate problems			
			4	severe problems			
			5	unable to do usual activities			
			97/98/99	Missing			
	HealthRelatedPain		1	no pain or discomfort			
			2	slight pain or discomfort			
			3	moderate pain or discomfort			
			4	severe pain or discomfort			
			5	extreme pain or discomfort			
			97/98/99	Missing			
	HealthRelatedAnxiety		1	not anxious or depressed			
			2	slightly anxious or depressed			
			3	moderately anxious or depressed			
			4	severely anxious or depressed			
			5	extremely anxious or depressed			
			97/98/99	Missing			
	CALC_EQ5D5L	total EQ5D score					

Required Field (please X)	Variable	Variable Label	Values	Value Label	comment
	PainStoppedActivity	has pain...	0	no	
	PainStoppedClimbing		2	yes	
	PainStoppedWalkingMoreThan200		97/98/99	Missing	
	PainStoppedWalkingLessThan200				
	PainCutDownActivities				
	PainAccomplishingLess				
	PainLimitedWork				
	PainWorkRequireExtraEffort				
	PainTroubleSleeping				
	PainPreventSocialActivities				
	PainToday	on a scale of 0-10 with 0	number 0-10		
	PainLast7Days		97/98/99	Missing	
	CALC_GeriatricPainMeasure	Geriatric Pain Measure t	number 0-40		
			97/98/99	Missing	

Required Field (please X)	Variable	Variable Label	Values	Value Label	comment		
	SomeoneToTalkTo_LoninessScale	...the extent to which the statements apply	1	yes			
	MissHavingCloseFriend_LoninessScale		2	more or less			
	SenseOfEmptiness_LoninessScale		3	no			
	PeopleICanLeanOn_LoninessScale		97/98/99	missing			
	MissCompanyOfOthers_LoninessScale						
	CircleOfFriendsLimited_LoninessScale						
	PeopleITrustCompletely_LoninessScale						
	PeopleIFeelCloseTo_LoninessScale						
	MissHavingPeopleAround_LoninessScale						
	FeelRejected_LoninessScale						
	CallFriendsWheneverINeed_LoninessScal						
	CALC_emotionalLoneliness	yes or more or less on ? 2,3,5,6,9,10	number				
	CALC_missingemotionalLoneliness	number of mising items					
	CALC_socialLoneliness	no or more or less on ?1,4,7,8,11					
	CALC_misssingsocialLoneliness	number of missing items					
	CALC_totalLonliness	De Jong Gierveld scale sum of emotional and social loneliness	0-2	not lonely			
			3-8	moderately lonely			
			9-10	severely lonely			
			11	very serverly lonely			
			97/98/99	Missing			
	CALC_LonelinessScore	loneliness category	1	Not lonely			
			2	Moderately lonely			
			3	Severely lonely			
			4	Very severely lonely			
			97/98/99	Missing			
1	SatisfiedWithLife	basically satisfied with life	0	yes		1	yes
2	DroppedActivities	dropped many activities and interests	1	no		0	no
3	FeelEmpty	feel life is empty	97/98/99	Missing		97/98/99	Missing
4	GetBored	often get bored					
5	GetBored2 -?goodspirits?	in good spirits most of the time					
6	SomethingBadGoingToHappen	afraid that something bad is going to happen					
7	FeelHappy	feel happy most of the time					
8	FeelHelpless	often feel helpless					
9	PreferToStayAtHome	prefer to stay at home rather than going out and doing things					
10	ProblemsWithMemory	more problems with memory than most					
11	WonderfulToBeAlive	think it's wonderful to be alive					
12	Worthless	worthless the way you are now					
13	FullOfEnergy	full of energy					
14	SituationIsHopeless	situation is hopeless					
15	BetterOffThanYou	most people better off than you					
	GeriatricDepressionScale	total from 15 questions reverse score for items 1,5,7,11,13 >=5 suggests depressed	number 0-15				
			97/98/99	Missing			

Required Field (please X)	Variable	Variable Label	Values	Value Label	comment
	BounceBackQuickly_BriefResilienceScale	agreement with resiliance	1	strongly agree	
	HardTimeThroughStressfulEvents_BriefResilienceScale		2	agree	
	LongToRecoverFromStress_BriefResilienceScale		3	neutral	
	SnapBackFromSomethingBad_BriefResilienceScale		4	disagree	
	DifficultTimesLittleTrouble_BriefResilienceScale		5	strongly disagree	
	TimeToGetOverSetBacks_BriefResilienceScale		97/98/99	Missing	
	CALC_briefResilianceScale	sum/6 items reverse scorir	number 1-6 higer=more resiliant		
			97/98/99	Missing	
	SolveDifficultProblems_SelfEfficacyScale	agreement with general se	1	not at all true	
	WaysToGetWhatIWant_SelfEfficacyScale		2	hardly true	
	AccomplishMyGoals_SelfEfficacyScale		3	moderately true	
	DealWithUnexpectedEvents_SelfEfficacyScale		4	exactly true	
	HandleUnforeseenSituations_SelfEfficacyScale		97/98/99	Missing	
	SolveMostProblems_SelfEfficacyScale				
	RemainCalm_SelfEfficacyScale				
	FindSeveralSolutions_SelfEfficacyScale				
	ThinkOfASolution_SelfEfficacyScale				
	HandleWhateverComes_SelfEfficacyScale				
	CALC_SelfEfficacyScale	Total self-efficacy score- hi	number 10-40		
			97/98/99	Missing	

NB: Individual items in ELSA are pulled from other assessm				
WalkingOneBlock_SF-36Activities				
SitUprightForTwoHours				
AbleToGetUpFromChair				
ClimbingSeveralStairs_SF-36Activities				
ClimbingStairs_SF-36Activities				
BendingKneelingStooping_SF-36Activities				
PenFromHand				
ModerateActivities_SF-36Activities				
VigorousActivities_SF-36Activities				
CoinFromTable				
HealthRelatedSelfCare				
HealthRelatedMobility				
Bathing				
Feeding				
Transfers				
ToiletUse				
BustrainTimetable				
MakeYourslefAHotSnack				
DoOwnShopping_NEADLPartTwo				
UseTelephone_NEADLPartTwo				
EFSForgetMedication				
ManageOwnMoney_NEADLPartTwo				
DoOwnHousework_NEADLPartTwo				
ExhaustionPartA				
Sleep				
GoodSpirits				
WonderfulToBeAlive				
FeelHappy				
ExhaustionPartB				
Hypertension				
Atherosclerotic				
MyocardialInfarction				
HeartFailure				
DiabetesMellitus				
Cerebrovascular				
COPD				
RespiratoryAsthma				
Osteoporosis				
ExcludingNonmelanoma				
Parkinson				
NeurologicalDementia				
eyesight 4 & 5				
hearing & vision 6 & 7				
FallsInLast12Months				
FracturedHipWristBackbone				
Joint				
geriatric pain measure 3 or				
MoCAOrientationDate				
MoCAOrientationMonth				
MoCAOrientationYear				
MoCAOrientationDay				
MoCASentenceRepetitionOne				
MoCAVerbalFluency				
delayed recall combines 5 questions				
SF-36HealthSurvey'				
GeriatricDepressionScale >= 5				
CALC_totalLonliness) >= 3				
AtrialFibrillation				
AtrialFlutter				
Osteoarthritis				
RheumatoidArthritis				
OtherArthritis				
Arthritis				
Anxiety				
Depression				
RecognisingPeople				
ReadingNewsprint				
HearingInQuietRoom				
HearingInLoudRoom				
PainStoppedWalkingMoreThan200				
PainStoppedWalkingLessThan200				
MoCADelayedRecallFace				
MoCADelayedRecallVelvet				
MoCADelayedRecallChurch				
MoCADelayedRecallDaisy				
MoCADelayedRecallRed				
CALC_ELSAFrailtyIndex				

NB: Individual items in Fried are pulled from other assessment data where possible to avoid overburdening the participant								
CALC_DemiSpanHeight								
CALC_BMI								
WalkTime								
WeightLoss								
ExhaustionPartA								
ExhaustionPartB								
DominantHand								
DominantHandFirstAttempt								
DominantHandSecondAttempt								
DominantHandThirdAttempt								
NonDominantHandFirstAttempt								
NonDominantHandSecondAttempt								
NonDominantHandThirdAttempt								
CALC_DominantMeanGripStrength								
CALC_NonDominantMeanGripStrength								
CALC_FriedTotalScore								

Enquiries to lesley.brown@bthft.nhs.uk
Project number: [office use only]

CARE75+ DATA REQUEST FORM

This form is to be used for all data request purposes including; sampling, preparatory work and research.

The request contact will be responsible for the transfer, storage and governance of the data in line with the data sharing agreement (appendix 1).

All sections *must* be completed.

1. Office use only

Data request number	
Date of request	
Date of review by DRRC	
Date of DRRC query	
Date of query review	
Data sent	

____

2. Request contact

Study Name:	
Requested by:	
Contact:	
Email	
Telephone	
Job role	
Organisation	
Date of request	
Date required	

For peer review only - http://bmjopen.bmj.com/site/about/guidelines.xhtml

Enquiries to lesley.brown@bthft.nhs.uk
Project number: [office use only]

3. Data Handling. If anyone else is expected to handle (i.e. view/analyse/transfer/store) these data in association with the study named in section 1, please list them here:

Name	Affiliation	Title	Role in the project

For peer review only

4. Purpose of request

Sampling i.e. participant contacts	
Scoping exercise e.g feasibility/protocol development	
Research e.g. analysis for funded/approved projects	

5. Research question and brief summary of research (350 words)

6. What type of data do you require?

Individual identifiable data (contains personal details)	
Pseudo-anonymised (contains unique id for data linkage)	
Anonymised (contains no identifiable details)	

For peer review only - http://bmjopen.bmj.com/site/about/guidelines.xhtml

Enquiries to lesley.brown@bthft.nhs.uk
Project number: [office use only]

7. What stage do you require (NB: full data will not be available for all participants)

Baseline	
Six month follow-up	
12 month follow-up	
24 month follow-up	
48 month follow-up	
Latest time-point	

8. Selection criteria

Included if:	
But excluded if:	

9. Specific data items required

Date of Assessment required?		
**Data dictionary sheet title**	**Variable name** (please cut and paste from data dictionary)	
Contact information		
Personal details		
Housing, Living Circumstance		
Education, Occupation		
Family Data		
Formal and Informal Support		
Smoking, Alcohol		
Hearing, Eyesight		
SF-36		

For peer review only

Enquiries to lesley.brown@bthft.nhs.uk
Project number: [office use only]

MoCA	
Co-morbidities	
Falls	
Medications	
Activities of Daily Living	
Height, Weight, BP, Grip	
Timed Up and Go, Walking	
Frailty	
Quality of Life and Sleep	
Pain	
Loneliness, Depression	
Resilience, Self-Efficacy	

10. Please can you provide details of your intended output (for example, publication or report)

For peer review only

BMJ Open

The Community Ageing Research 75+ Study (CARE75+): an experimental ageing and frailty research cohort

Journal:	*BMJ Open*
Manuscript ID	bmjopen-2018-026744.R1
Article Type:	Protocol
Date Submitted by the Author:	23-Nov-2018
Complete List of Authors:	Heaven, Anne; Bradford Institute for Health Research, Academic Unit of Elderly Care & Rehabilitation<br>Brown, Lesley; Bradford Institute for Health Research, Academic Unit of Elderly Care & Rehabilitation<br>Young, John; University of Leeds, Academic Unit of Elderly Care & Rehabilitation<br>Teale, Elizabeth; University of Leeds, Academic Unit of Elderly Care & Rehabilitation<br>Hawkins, Rebecca; University of Leeds, Academic Unit of Elderly Care and Rehabilitation<br>Splisbury, Karen; University of Leeds, School of Healthcare<br>Mountain, Gail; University of Bradford, Centre for Applied Dementia Studies<br>Young, T; The University of Sheffield, School of Health and Related Research<br>Goodwin, Victoria; University of Exeter, PenCLAHRC<br>Hanratty, Barbara; Newcastle University, Institute of Health and Society<br>Chew-Graham, Carolyn; University of Keele, Research Institute for Primary Care & Health Sciences<br>Brundle, Caroline; Bradford Institute for Health Research, Academic Unit of Elderly Care & Rehabilitation<br>Mahmood, Farhat; Bradford Institute for Health Research, Academic Unit of Elderly Care & Rehabilitation<br>Jacob, Ikhlaq; Bradford Institute for Health Research, Academic Unit of Elderly Care & Rehabilitation<br>Daffu-O'Reilly, Amrit; University of Leeds, Academic Unit of Midwifery, Social Work, Pharmacy and Counselling and Psychotherapy<br>Clegg, Andrew; University of Leeds, Academic Unit of Elderly Care & Rehabilitation
**Primary Subject Heading**:	Geriatric medicine
Secondary Subject Heading:	Epidemiology, Health services research
Keywords:	frailty, CARE75+, cohort, TwiCs, ageing

SCHOLARONE™
Manuscripts

The Community Ageing Research 75+ Study (CARE75+): an experimental ageing and frailty research cohort

Authors: Anne Heaven[1], Lesley Brown[1], John Young[1], Elizabeth Teale[1], Rebecca Hawkins[1], Karen Spilsbury[2], Gail Mountain[3], Tracey Young[4], Victoria Goodwin[5], Barbara Hanratty[6], Carolyn A. Chew Graham[7], Caroline Brundle[1], Farhat Mahmood[1], Ikhlaq Jacob[1], Amrit Daffu-O'Reilly[8], Andrew Clegg[1]

[1]Academic Unit of Elderly Care & Rehabilitation, University of Leeds, Bradford Institute for Health Research, Bradford Teaching Hospitals NHS Foundation Trust, Bradford, BD9 6RJ, and NIHR CLAHRC Yorkshire & Humber

[2]Academic Unit: Adult, Child and Mental Health Nursing, School of Healthcare, University of Leeds, LS2 9JT, and NIHR CLAHRC Yorkshire & Humber

[3]Centre for Applied Dementia Studies, Faculty of Health Studies, Bradford University, BD7 1DP, and NIHR CLAHRC Yorkshire & Humber

[4] School of Health and Related Research, University of Sheffield, Regent Court, 30 Regent St, Sheffield, S1 4DA, and NIHR CLAHRC Yorkshire & Humber

[5] NIHR CLAHRC South West Peninsula, University of Exeter Medical School, EX1 2LU

[6] Institute of Health & Society, Newcastle University Institute for Ageing, Biomedical Research Building, Newcastle University, NE4 5PL

[7]Research Institute for Primary Care & Health Sciences, Keele University, Staffordshire ST5 5BG and NIHR CLAHRC West Midlands

[8] Academic Unit of Midwifery, Social Work, Pharmacy and Counselling and Psychotherapy, University of Leeds, LS2 9JT

Email addresses

anne.heaven@bthft.nhs.uk

lesley.brown@bthft.nhs.uk

For peer review only

john.young@bthft.nhs.uk

elizabeth.teale@bthft.nhs.uk

r.hawkins@leeds.ac.uk

k.spilsbury@leeds.ac.uk

g.mountain@bradford.ac.uk

t.a.young@sheffield.ac.uk

v.goodwin@exeter.ac.uk

barbara.hanratty@newcastle.ac.uk

c.a.chew-graham@keele.ac.uk

caroline.brundle@bthft.nhs.uk

farhat.mahmood@bthft.nhs.uk

ikhlaq.jacob@bthft.nhs.uk

a.k.daffuoreilly@leeds.ac.uk

a.p.clegg@leeds.ac.uk

Corresponding author: Andrew Clegg (a.p.clegg@leeds.ac.uk)

Word count: 4,710 words (main manuscript)

For peer review only

For peer review only - http://bmjopen.bmj.com/site/about/guidelines.xhtml

Abstract

Introduction: The Community Ageing Research 75+ Study (CARE75+) is a longitudinal cohort study collecting an extensive range of health, social and economic data, with a focus on frailty, independence and quality of life in older age. CARE75+ is the first international experimental frailty research cohort designed using Trial within Cohort (TwiCs) methodology, to align applied epidemiological research with clinical trial evaluation of interventions to improve the health and wellbeing of older people living with frailty.

Methods and analysis: Prospective cohort study using a trial within cohort (TwiCs) design. One thousand community-dwelling older people (≥75years) will be recruited from UK general practices. Nursing home residents, those with an estimated life expectancy of three months or less and people receiving palliative care will be excluded. Data collection assessments will be face-to-face in the person's home at baseline, six months, 12 months, 24 month and 48 months, including assessments of frailty, cognition, mood, health-related quality of life, comorbidity, medications, resilience, loneliness, pain and self-efficacy. A modified protocol for follow-up by telephone or web-based will be offered at six months. Consent will be sought for data linkage and invitations to additional studies, including intervention studies using the TwiCs design. A blood sample bio-bank will be established for future basic science studies.

Ethics and dissemination: CARE75+ was approved by the NRES Committee Yorkshire & the Humber - Bradford Leeds 10th October 2014 (14/YH/1120). Formal written consent is sought if an individual is willing to participate and has capacity to provide informed consent. Consultee assent is sought if an individual lacks capacity.

Study results will be disseminated in peer-reviewed scientific journals and scientific conferences. Key study results will be summarised and disseminated to all study participants

For peer review only

For peer review only - http://bmjopen.bmj.com/site/about/guidelines.xhtml

via newsletters, local older people's publications and local engagement events. Results will be reported on a bespoke CARE75+ website.

Trial registration

ID ISRCTN16588124 (date of registration 26th February 2016 - retrospectively registered; date of first participant recruitment January 2015)

Strengths and limitations of this study

- CARE75+ is a prospective cohort study recruiting older people aged 75 and over, designed using Trial within Cohort (TwiCs) methods, collecting an extensive range of demographic, health and socioeconomic data at baseline, six, 12, 24 and 48 months.
- Our recruitment strategy, including home consent visits, home assessments, and use of researchers with community language skills, is designed to optimise the recruitment of older people across the frailty spectrum.
- CARE75+ will recruit participants from a variety of ethnic backgrounds and those with advanced frailty who are often underrepresented in research.
- Care home residents are not eligible for the study, aligned with the TwiCs design, meaning that findings cannot be generalised to this group of especially frail older people.
- CARE75+ is a cohort of high strategic relevance, which will help shape future UK and international health and research policy in ageing and frailty.

For peer review only

Introduction

Global ageing demographic projections indicate that there will be two billion people aged over 65 worldwide by 2050 [1, 2]. Frailty is an especially problematic expression of population ageing, with profound implications for planning and delivery of health and social care services globally. It is a condition characterised by loss of biological reserves, failure of homeostatic mechanisms and increased vulnerability to adverse outcomes following relatively minor stressor events [3, 4]. Thus, a mild infection, new medication, or minor surgery can result in a sudden, disproportionate change in health status or functional status for an older person with frailty, for example a change from independence to dependence, a fall, or development of delirium. Frailty is also associated with an increased risk of a range of adverse outcomes, including future disability, admission to hospital, long-term care residence and mortality [5].

To date, the healthcare response to frailty has been predominantly reactive and secondary care based. However, there is increasing recognition that frailty should be identified and managed as a long-term condition with preventative and proactive care models [6-8]. Furthermore, with the widespread introduction of robustly developed tools to detect frailty in primary care such as the electronic Frailty Index (eFI) based on routinely available primary care electronic health record (EHR) data in the UK [9], primary care teams can now more readily and reliably identify older people with frailty within their patient populations. These novel approaches are providing opportunities to develop and deliver services according to frailty status rather than chronological age.

Improved management of frailty requires an integrated approach spanning primary care, secondary care and social services that incorporates consideration of frailty transitions and health trajectories. Where possible, integrated care pathways should be developed and implemented based on suitably targeted, evidence-based interventions. Although

For peer review only - http://bmjopen.bmj.com/site/about/guidelines.xhtml

recruitment to ageing and frailty observational research studies has historically been relatively high [10, 11], recruitment rates to clinical trials of frailty interventions have frequently been low.

The Trial within Cohort (TwiCs) design [12] is an innovative research methodology that has the potential to enhance participation of older people with frailty in a range of studies including clinical trials, and to increase the capacity to conduct high quality frailty research [13]. The TwiCs design has several key features including the establishment of an observational cohort to both provide longitudinal data and function as a recruitment platform for multiple trials and other research studies. Each individual trial uses random selection of some (not all) participants from the cohort; intervention-centred information and consent is applied. The process aims to replicate the real world of routine health care by taking informed consent only from those randomised to receive an intervention, as the ongoing cohort study provides a natural control group.

Methods and analysis

Aim

Our aim is to establish a longitudinal cohort of older people to investigate frailty, disability and quality of life in older age and to act a recruitment platform for future studies (sub-studies) to enable the development and evaluation of interventions to improve outcomes for older people.

Patient and public involvement

We have established a Frailty Oversight Group (FOG) as a central component of the CARE75+ study. The FOG comprises a core reference group of four key individuals with links to local community organisations involved in the support of older people living with frailty, and a minority ethnic group advocate from the local authority. The FOG play a key

For peer review only - http://bmjopen.bmj.com/site/about/guidelines.xhtml

role in developing research questions for the cohort, including reviewing any proposed data analyses or nested studies.

The FOG had close involvement in developing and piloting the outcome assessment schedule for the study, highlighting the need to include measures that extend beyond traditional health domains into areas such as loneliness and resilience in later life. The FOG contributed to the development of all study materials, including invitation letters and participant information sheets, to ensure alignment with the needs of older people. Results are disseminated widely to participants, including through regular newsletters and an annual celebration event.

Design

A multi-site, community-based cohort study using a TwiCs design [12].

Inclusion criteria

Community dwelling older people aged ≥75 years.

Exclusion criteria

People with terminal cancer, life expectancy of three months or less and people in receipt of palliative care services will be excluded. Care home residents and people living at home who are bedbound will be excluded. However, we will attempt to follow-up people who transition to a care home during the course of the study.

Assessments

The CARE75+ assessment includes detailed information on the demographic, health and social circumstances of participants. An extensive range of measures are collected using validated instruments, including assessments of frailty, cognition, mood, health-related

For peer review only - http://bmjopen.bmj.com/site/about/guidelines.xhtml

quality of life, comorbidity, medications, resilience, loneliness and self-efficacy (table 1). The selected measures have been carefully chosen to ensure that CARE75+ includes measures with the necessary validity, reliability and responsiveness to enable both applied epidemiological investigation and randomised trial evaluation of future interventions to improve outcomes.

List of current assessments

- Demographic information (age, sex, ethnicity, marital status, living circumstances, housing type, education, previous occupation).
- Family networks and informal support (self-report).
- Resource use: GP, hospital and outpatient admissions. Use of aids and adaptations (self-report)
- Formal care (self-report).
- Smoking habits & alcohol consumption (self-report).
- Vision LogMar Vision test [14] (Thompson Software Solutions) [15].
- Hearing (the Whispered Voice test) [16].
- Sleep (self-report).
- Medication (prescribed) details (name, dose, frequency) will be collected from Primary Care Electronic Health Records (EHR). Non-prescribed medication will be self-reported.
- Cognitive function assessed using the Montreal Cognitive Assessment (MoCA) [17], a brief cognitive assessment instrument. The MoCA assesses different cognitive domains: attention and concentration; executive function; memory; language; conceptual thinking; calculations; and orientation. The total possible score is 30, with higher scores indicating better cognitive function, and a score of ≥26 considered normal.

For peer review only - http://bmjopen.bmj.com/site/about/guidelines.xhtml

- Comorbidities data, collected via the primary care electronic health record (EHR), and by self-report using the Katz comorbidity questionnaire [18]. This questionnaire asks questions on various health conditions requiring a 'yes' or 'no' response.
- General health and health related quality of life, using the RAND short-form 36-Item Health Survey (SF36) [19] which includes 36 questions spanning eight health domains: physical functioning; bodily pain; role limitations due to physical health problems; role limitations due to personal or emotional problems; general mental health; social functioning; energy/fatigue; and general health perceptions. It also includes a single item that provides an indication of perceived change in health. The SF36 enables calculation of Physical Component Summary (PCS) and Mental Component Summary (MCS) scores, and derivation of an overall health utility score, the short-form 6 dimension score (SF6D) suitable for use in economic evaluations [20].
- Health related quality of life using the EuroQol five dimension health questionnaire (five-level version) EQ5D-5L [21]. The EQ5D-5L five dimensions are: mobility, self-care, usual activities, pain/discomfort and anxiety/depression. Each dimension has 5 levels of severity: no problems, slight problems, moderate problems, severe problems and extreme problems. The scores for each of the five dimensions are combined in a five digit number representing health status that can be converted into a utility index (0 for dead, 1 for perfect health and negative values for states worse than death) for use in economic evaluations.
- Basic activities of daily living (ADL) using the Barthel index (BI) [22]. The BI assesses functional status on a 20 point scale by recording ability to complete ten basic activities of daily living; bathing, bladder function, bowel function, dressing, feeding, grooming, mobility, stairs, toilet use and transfers. Higher scores indicate greater independence.

For peer review only - http://bmjopen.bmj.com/site/about/guidelines.xhtml

- Instrumental ADL, measured using the Nottingham Extended Activities of Daily Living (NEADL) scale [23]. The NEADL includes questions on everyday activities in the domains of mobility, kitchen, domestic and leisure and is scored between 0 and 66, with higher scores indicating greater independence.
- Measures of frailty:
  - Research standard 60 item frailty index, based on the cumulative deficit model of frailty [24], and previously validated as part of the English Longitudinal Study of Ageing (ELSA) [25]. The frailty index score is calculated an equally weighted proportion of the number of deficits present in an individual relative to the total possible.
  - The phenotype model of frailty, based on the five physical characteristics as reported in the original Cardiovascular Health Study (slow walking speed, weight loss, exhaustion, weak grip strength, low energy expenditure) [3]. Slow walking speed is assessed by a timed three metre walk and results stratified by height and gender using values described in the original Cardiovascular Health Study, from which the phenotype model was derived [3]. Weight loss is determined by the following question. “In the last year, have you lost more than 10 pounds unintentionally?” Exhaustion is identified using the following questions: “How often in the last week do you feel that everything you did was an effort?” and “could not get going?”. Responses are: rarely or none of the time (< 1 day) = 0; some or a little of the time (1 - 2 days) = 1; moderate amount of the time (3 - 4 days) = 2; most of the time = 3. If the participant answers “2” or “3” to either question they meet the criterion for exhaustion. Hand grip strength is assessed using a Jamar dynamometer and stratified using criteria from the Cardiovascular Health Study [3] with the mean of three attempts calculated for the dominant and non-dominant hand. Low activity is assessed using data obtained from the Physical Activity domain of the SF36

For peer review only

[19]. Those with no characteristics are identified as fit, one or two characteristics as pre-frail and three to five characteristics as frail.

- The seven category Clinical Frailty Scale (CFS) [24], which is a validated measure of frailty based on clinical descriptors and pictographs, designed for specialist and non-specialist use in routine clinical practice. The CFS is an ordinal measure, with scores ranging from one (fit) to seven (severe frailty).
- The Edmonton Frail Scale (EFS) [26], which is a validated frailty measure designed for specialist and non-specialist use that records information on nine frailty domains (cognition, general health, functional independence, social support, medication use, nutrition, mood, continence, functional performance). The EFS is scored out of a total of 17, with higher scores indicating increasing frailty.
- The electronic frailty index (eFI) score [9], based on the cumulative deficit model of frailty, including 36 variables recorded in the primary care EHR as part of routine care. The eFI score is calculated as an equally weighted proportion of the number of deficits present in an individual relative to the total possible. The eFI enables identification of frailty categories (fit, mild frailty, moderate frailty, severe frailty) and is obtained directly from the primary care EHR.

- Height weight and body composition: researcher assessment using bio-impedance scales (Marsden BFA-220P Body fat analyser). Weight loss is obtained by self-report at baseline and calculated from previously recorded weight data at follow-up time-points.
- Blood pressure (Life source auto inflation blood pressure monitor): sitting (three times), standing (once).
- Mobility, calculated using the timed-up-and-go-test (TUGT) [27]. The TUGT assesses a person's mobility and requires both static and dynamic balance. It measures the

For peer review only

time that a person takes to rise from a chair, walk three metres, turn around, walk back to the chair, and sit down. A person's usual walking aid is used if needed. People completing the test in less than 20 seconds tend to be independently mobile, able to get in and out of a chair without assistance and climb stairs. People completing the test in 20-29 seconds demonstrate greater variability in mobility, balance and functional ability. Completion of the TUGT in 30 seconds or more identifies people likely to require assistance with getting in and out of a chair, climbing stairs and leaving the house.

- Pain, measured using the Geriatric Pain Measure Short Form [28]. This questionnaire includes items of pain intensity (current and last 7 days), and dichotomous items on how pain is impacting on a person's mobility, ability to accomplish tasks and to sleep. Items are combined to derive an overall summary score.
- Loneliness recorded using the 11 item De Jong Gierveld Loneliness scale [29]. Sub-categories of social and emotional loneliness are calculated and a total score is derived enabling identification of categories: not lonely; moderately lonely; severely lonely; very severely lonely.
- Resilience, measured using the Brief Resilience scale (BRS) [30]. The six items in the BRS include five response options, enabling calculation of an overall score ranging from 1 to 6, with higher scores indicating greater resilience.
- Self-efficacy, measured using the General Self-Efficacy Scale [31]. This scale lists ten items with four response options enabling generation of a summary score ranging from 10 to 40, with higher scores indicating greater resilience.
- Low mood, assessed using the Geriatric Depression Scale Short-Form with a score of ≥5 indicating an abnormal low mood state [32].
- Self-reported falls.

For peer review only - http://bmjopen.bmj.com/site/about/guidelines.xhtml

- Full blood count (Leeds & Bradford sties only): haemoglobin and mean cell volume; red blood cell (RBC) count; mean cell haemoglobin concentration; mean cell haemoglobin; RBC distribution width, white blood cell count (including neutrophils, lymphocytes; monocytes; eosinophils; basophils); and platelets
- Frozen blood aliquots (Leeds & Bradford sites only) for future biochemical analysis, including:
  - Routine biochemistry and haematology: renal profile; liver profile; serum albumin; bone profile; glucose; glycosylated haemoglobin; lipid profile; uric acid; clotting.
  - Endocrine function: cortisol; thyroid function; IGF-1; DHEAS; testosterone; oestradiol; vitamin D; PTH; neuronal specific protein.
  - Immune function: highly sensitive CRP; inflammatory cytokines; rheumatoid factor; markers of immunosenescence.
  - Nutritional markers: vitamin A; vitamins B2, B6, B12; vitamin C; ferritin; folate; homocysteine.
  - Biomarkers of ageing: DNA repair capacity; telomere length; markers of oxidative stress.
  - Genetic markers: DNA; RNA; plasma.

The CARE75+ data dictionary is available as an appendix file (see Additional file 1).

Assessment schedule

Participants will be assessed at baseline, six, 12, 24 and 48 months. Face-to-face assessments will be conducted in the participant's home. The feasibility of a modified, telephone-based or web-based assessment protocol will be tested at the six month time point for participants who are willing and able to undertake assessments in the alternative formats.

For peer review only

For peer review only - http://bmjopen.bmj.com/site/about/guidelines.xhtml

The assessment schedule for CARE75+ (baseline, six, 12, 24 and 48 months) has been carefully designed to accelerate the frailty translational research pathway by aligning robust epidemiological investigation with the typical follow-up schedule for feasibility and definitive trials of interventions.

Sample size

The CARE75+ study will generate a comprehensive dataset for applied epidemiological research and will act as a recruitment platform for additional studies (sub-studies), including qualitative studies as well as randomised controlled trials (RCTs) using TwiCs methods. Therefore, the initial recruitment target is based on appropriate sample size calculations for pilot RCTs of interventions to inform the design of future definitive RCTs alongside applied epidemiological investigation of modifiable component of frailty.

Previous observational studies involving older people with frailty have identified that between 600 - 1000 participants are required for reliable estimates of the main effects [33]. Following an initial pilot phase involving 200 participants to test recruitment methods and gather data on rates of assent to participation in future trials, we plan to recruit 1000 participants over a four year period. Previous observational studies involving the oldest old have reported 18 month attrition rates of around 25% due to mortality and withdrawal of consent [10]. As our cohort will include older people with frailty who are at increased risk of adverse outcomes we plan to recruit a minimum of 250 participants per year thereafter, to maintain a legacy cohort for future clinical trials. Findings from the CARE75+ study will inform the design of a future definitive experimental frailty research cohort of sufficient size to nest a series of definitive intervention trials targeted at a range of potentially modifiable components of frailty, including people living with different frailty severity grades.

For peer review only

For peer review only - http://bmjopen.bmj.com/site/about/guidelines.xhtml

Recruitment

We will work with general practices to identify and recruit participants in primary care. Following initial piloting of recruitment methods in Bradford and Leeds, West Yorkshire, we will extend recruitment to other practices in England, using the skills and experience of staff within the National Institute for Health Research Clinical Research Networks (NIHR CRN).

The CARE75+ recruitment, consent, assessment and follow-up process is summarised in a study flowchart (figure 1).

For peer review only

Participant contact

Potential participants will be posted a study invitation pack containing a letter of invitation, a user-friendly participant information leaflet with photographs of the research staff involved in the home visits, and a supporting letter from their general practice. Potential participants who are not interested in participating in the study will be invited to contact their general practice to opt out. If potential participants do not opt out, contact details of eligible participants will be provided to the research team via a secure email system. The invitation letter will be followed up after two weeks with a telephone call from a researcher to discuss the study in more detail. If initial interest is expressed, the researcher and potential participant will arrange a home visit for an in-depth discussion of the study, where informed, written consent to participate will be sought.

The recruitment methods take into account the range of physical and cognitive challenges encountered by older people. Experience from previous cohort studies involving older people with frailty, disability and cognitive impairment has demonstrated that direct telephone calls or in-person visits are the only reliable methods of finding out whether potential participants are interested in participating, and may be preferred because they are seen as less of a burden [33]. Recruitment procedures will ensure that an older person with frailty receives all

For peer review only - http://bmjopen.bmj.com/site/about/guidelines.xhtml

the necessary information to make an informed decision about participation. Procedures have been developed in close partnership with lay representatives through our Patient and Public Involvement Frailty Oversight Group [34], established as part of the National Institute for Health Research Collaboration for Leadership in Applied Health Research and Care, Yorkshire and Humber (NIHR CLAHRC Yorkshire & Humber) programme.

Participant consent

Following initial telephone contact, researchers will visit participants who express an interest in participation and verbally explain the study in detail, including providing a comprehensive study information leaflet. Potential participants will be able to have an advocate, family member or friend present and will be offered 48 hours to reflect on the information before deciding to consent. For individuals whose first language is not English, a community language speaking researcher will be assigned where possible, or a suitable advocate identified.

Researchers will assess an individual's capacity to consent in accordance with the Mental Capacity Act (MCA) [35]. Formal written consent will be sought if an individual is willing to participate and has capacity to provide informed consent. The consent form will detail all processing and disclosure of the information collected including data analysis, data linkage, providing contact details to future researchers, and the storage and use of blood samples. Some components of the consent will be optional (for example taking and storing blood, consenting to be approached about other studies). Written consultee assent will be sought if individual participants do not have capacity to consent. Independent consent to participate will be obtained for participation in any future trial.

For peer review only

For peer review only - http://bmjopen.bmj.com/site/about/guidelines.xhtml

Data collection methods

We plan face-to-face data collection, but we will test the feasibility of telephone or web-based modified data collection procedure for participants who are willing, and able, at the six month time point.

Prescribed medications, comorbidity data and eFI scores will be obtained from general practice EHRs, extracted using standardised reporting templates developed for the SystmOne [36] and EMISWeb [37] primary care EHR systems.

All data will be collected using a bespoke electronic data capture application (EDCA), the CARE75+app developed and tested by Tigerteam Software Ltd[a]. Blood samples will be collected at baseline and 12 months from participants in the Bradford and Leeds sites.

Research staff training

Research staff will undertake a bespoke training programme, depending on skills and experience, including: the Mental Capacity Act [35]; research with older people; phlebotomy and safeguarding vulnerable adults. Additionally, staff will receive training in completion of the individual assessment measures and data entry into the Electronic Data Capture Application (EDCA).

Plans to promote participant retention and complete follow-up

We will seek broad and enduring consent for data linkage and use of collected data following withdrawal or death, aligned with Medical Research Council (MRC) guidelines for maximising the use of cohort data [38].

We will post newsletters to participants at least twice a year to provide study updates and encourage continued engagement. We will hold annual engagement events, where feasible

For peer review only - http://bmjopen.bmj.com/site/about/guidelines.xhtml

to do so, and promote the study locally via affiliated newsletters (e.g. Age UK Voice magazine) and local forums.

Data entry, coding, security and storage

The EDCA will comprise two main components: a Data Collection Application (DCA) and Back Office System (BOS) containing personal identifiable information. The DCA will run on Microsoft Windows platform using an encrypted embedded database to temporarily store data. The BOS database will be on a Microsoft SQL server hosted at Bradford Teaching Hospitals NHS Foundation Trust (BTHFT). All data will be captured off-line in the community. Data will be uploaded regularly to ensure no identifiable data remains on the portable device for longer than 48 hours. Named researchers will have access to the individual details only whilst data collection takes place. A participant's details will only be released to one researcher at a time via the BOS management system. Access to modules and functions of both the DCA and BOS will governed by usernames, passwords and role specific access permissions, to maximise data security.

Remote site data (outside BTHFT) and the on-line completion forms (optional 6 month follow-up protocol) will be transferred to the BIHR-CARE database via the web application auecr.bradfordhospitals.nhs.uk hosted on the web server bhts-bihrweb. The site will be protected by SSL certificates, to encrypt the transfer of data over the internet. Access to the web application https://auecr.bradfordhospitals.nhs.uk on the server bhts-bihrweb will be restricted and protected by the Threat Management Gateway (TMG) software and SSL certificates. Remote site administrators and researchers will only have access to their own local participants.

Access to the BIHR-CARE database information will be based on role specific permissions. The chief investigator and project manager will have access to all data, at all levels for

For peer review only - http://bmjopen.bmj.com/site/about/guidelines.xhtml

administration and governance purposes. Local site administrators will have access to local participant details. Researchers will have access to individual (site specific) case information only at the time of data collection. Researchers will have a maximum of three participants available on portable devices (laptops) at any one time. Pathology laboratory staff will have access to blood sample data entry pages only. Statisticians and other members of the CARE75+ research team will only have access to pseudo anonymised i.e. those with unique identifiers for use in data linkage or anonymous data. Individual participants will be limited to access to a blank follow up questionnaire to complete and submit. All submitted data is final and data access is only available to the Super Administrator at BTHFT.

Data quality

Data quality will be enhanced by integral features of the data capture software, which will identify missing data and outlying values in real time. The software will automatically calculate the total scores for composite assessments. This will increase research efficiency and research data quality by reducing resource required for data cleansing, coding for analysis and reduce inputting errors.

Statistical methods

We plan interim data analyses after the completion of each stage i.e. baseline, 6, 12, 24 and 48 month follow-up of the study. We will assess frailty transitions using multivariate statistical methods. We will estimate health and social care resource use associated with frailty using economic modelling techniques.

We will conduct applied epidemiological investigation of the association between potentially modifiable components of frailty and outcomes, including: how pain modifies the association between frailty and disability; how resilience modifies the association between frailty and disability; and the association between frailty, mood and outcomes. We will investigate

For peer review only - http://bmjopen.bmj.com/site/about/guidelines.xhtml

construct and criterion validity of a range of tests collected [39]. We will assess frailty transitions using transition modelling. We will estimate health and social care resource use associated with frailty using economic modelling techniques.

Methods for any additional analyses (subgroup and adjusted analyses)

Data will be made available to external investigators upon request and reviewed by the CARE75+ Data Request Review Committee (DRRC), comprising the Chief Investigator, CARE75+ project manager, database manager, an independent member and independent lay representative from the Frailty Oversight Group [34].

The ethnic diversity of our planned recruitment sites will enable the investigation of ageing, frailty and disability in different cultural contexts.

Missing data

Methods for dealing with missing data will depend on the amount of missing data and patterns of missingness for individual variables as part of individual analyses. We will undertake sensitivity analysis to investigate the impact of missing data and we will explore the use of appropriate imputation methods.

Ethics and dissemination

This study was approved by the NRES Committee Yorkshire & the Humber - Bradford Leeds on the 10th October 2014 (14/YH/1120). CARE75+ is an observational study with low risk to participants. Cohort governance will be provided by the National Institute for Health Research Collaboration for Leadership in Applied Health Research and Care Yorkshire & Humber (NIHR CLAHRC YH) Frailty Theme [40] Operational Group comprised of the Theme Leads, Theme Manger, Project Managers and co-applicants. Independent scrutiny will be provided by the Frailty Oversight Group [34], which is comprised of lay members with

For peer review only - http://bmjopen.bmj.com/site/about/guidelines.xhtml

For peer review only

networks into the wider community of older people in Bradford. Day to day monitoring, including data quality checks and validations will be the responsibility of a dedicated database manager.

Access to data

BTHFT will be the data controller for CARE75+. Data will be made available to external researchers in accordance with CARE75+ data sharing protocols following review of the CARE75+ data dictionary (supplementary file 1) and completion of the CARE75+ data request form (supplementary file 2), review by the DRRC and completion of a data sharing transfer agreement.

Ancillary and post-study care

We anticipate that some participants may have potentially unmet care needs and may wish to discuss these with the researcher. We will ensure that researchers are able to signpost participants to local statutory and voluntary organisations (e.g. Age UK), or request a GP referral for social services assessment so that appropriate plans can be made for ongoing care.

Safeguarding issues identified during the assessment visits will be reported to the Research Project Manager who will then take advice from the Adult Safeguarding Co-ordinator in the relevant local authorities.

Dissemination policy

Study results will be disseminated in peer-reviewed scientific journals and submitted for consideration at local, national and international scientific conferences. Key study results will be summarised and disseminated to all study participants via newsletters, local older

For peer review only

For peer review only - http://bmjopen.bmj.com/site/about/guidelines.xhtml

people's publication (e.g. Voice magazine, Age UK) and local engagement events. Results will be reported on a bespoke CARE75+ website.

Research outputs using data from the CARE75+ study will be required to acknowledge the data source and funder using standardised wording. Additionally, studies involving participants identified from the cohort (sub-studies) will be required to acknowledge the CARE75+ cohort in all reports. The full protocol and participant level dataset will be made available to not-for-profit investigators. Enquiries should be made to the CARE75+ Chief Investigator and will be reviewed by the DRRC.

Discussion

CARE75+ will use novel TwiCs methodology to align applied epidemiological research into ageing and frailty with clinical trials of interventions, potentially accelerating the translational research pathway in this important area.

We describe methods to recruit a cohort of older people and collect an extensive range of health, social and economic outcome data. We plan to collect a range of validated measurements of frailty in CARE75+, including the eFI, which has been made available to every general practice in England through a national implementation project, facilitating the rapid translation of research findings into clinical practice. Our recruitment strategy, including home consent visits, home assessments, and use of researchers with community language skills, is designed to optimise the recruitment of older people across the frailty spectrum and from a variety of ethnic backgrounds, including those with advanced frailty who are often underrepresented in research. Care home residents are not eligible for the study, aligned with the TwiCs design, meaning that findings cannot be generalised to this group of especially frail older people.

For peer review only - http://bmjopen.bmj.com/site/about/guidelines.xhtml

Our vision for CARE75+ is a cohort of high strategic relevance, which will help shape future UK and international health and research policy in ageing and frailty.

Author contributions

Anne Heaven (AH): substantial contribution to the conception and design of the work; drafting the work and critical revisions; approval of final manuscript; accountable for all aspects of the work

Lesley Brown (LB): substantial contribution to the conception and design of the work; drafting the work and critical revisions; approval of final manuscript; accountable for all aspects of the work

John Young (JB): substantial contribution to the conception and design of the work; drafting the work and critical revisions; approval of final manuscript; accountable for all aspects of the work

Elizabeth Teale (ET) substantial contribution to the conception and design of the work; drafting the work and critical revisions; approval of final manuscript; accountable for all aspects of the work

Rebecca Hawkins (RH): substantial contribution to the conception and design of the work; drafting the work and critical revisions; approval of final manuscript; accountable for all aspects of the work

Karen Spilsbury (KS): substantial contribution to the conception and design of the work; drafting the work and critical revisions; approval of final manuscript; accountable for all aspects of the work

Gail Mountain (GM): substantial contribution to the conception and design of the work; drafting the work and critical revisions; approval of final manuscript; accountable for all aspects of the work

For peer review only

Tracey Young (TY): substantial contribution to the conception and design of the work; drafting the work and critical revisions; approval of final manuscript; accountable for all aspects of the work

Victoria Goodwin (VG): substantial contribution to the conception and design of the work; drafting the work and critical revisions; approval of final manuscript; accountable for all aspects of the work

Barbara Hanratty (BH): substantial contribution to the conception and design of the work; drafting the work and critical revisions; approval of final manuscript; accountable for all aspects of the work

Carolyn A. Chew Graham (CCG): substantial contribution to the conception and design of the work; drafting the work and critical revisions; approval of final manuscript; accountable for all aspects of the work

Caroline Brundle (CB): substantial contribution to the conception and design of the work; acquisition of data for the work; drafting the work and critical revisions; approval of final manuscript; accountable for all aspects of the work

Farhat Mahmood (FM): substantial contribution to the conception and design of the work; acquisition of data for the work; drafting the work and critical revisions; approval of final manuscript; accountable for all aspects of the work

Ikhlaq Jacob (IJ): substantial contribution to the conception and design of the work; acquisition of data for the work; drafting the work and critical revisions; approval of final manuscript; accountable for all aspects of the work

Amrit Daffu-O'Reilly (ADOR): substantial contribution to the conception and design of the work; acquisition of data for the work; drafting the work and critical revisions; approval of final manuscript; accountable for all aspects of the work

Andrew Clegg (AC): substantial contribution to the conception and design of the work; acquisition of data for the work; drafting the work and critical revisions; approval of final manuscript; accountable for all aspects of the work

For peer review only - http://bmjopen.bmj.com/site/about/guidelines.xhtml

Funding

This research was funded by the NIHR CLAHRC Yorkshire and Humber - www.clahrc-yh.nihr.ac.uk (study funding number IS-CLA-0113-10020) and supported by the NIHR CLAHRC South West Peninsula and West Midlands CLAHRC. The views and opinions expressed are those of the author(s), and not necessarily those of the NHS, the NIHR or the Department of Health and Social Care.

Acknowledgements

We are especially grateful to the substantial contributions of members of our Frailty Oversight Group: Anne Grice, Marilyn Foster, Chris McDermott, David Walker, Akhlak Rauf and Ernie Lloyd, who helped to finalise the protocol, test the assessment procedures and reviewed study materials. We would also like to thank the Newcastle 85+ study team for their invaluable advice and support in setting up CARE75+. Finally, we would like to acknowledge staff from the National Institute for Health Research Clinical Research Networks (NIHR CRNs) in North East & North Cumbria, South West Peninsula, West Midlands, and Yorkshire & Humber and all participating general practices for their support and enthusiasm in recruiting participants.

Competing interests

None.

Word count

4,710 words (main manuscript)

Additional Files

Additional file 1

For peer review only

Excel .xls

CAREDataDictionaryPub

Data collected during CARE75+ assessments, including all variable and value names and labels.

Figure 1

For peer review only

For peer review only - http://bmjopen.bmj.com/site/about/guidelines.xhtml

References

1. Kinsella KG, Phillips DR. Global aging: The challenge of success. Vol. 60. 2005: Population Reference Bureau Washington, DC.
2. United Nations. The World at Six Billion. 1999. http://www.un.org/esa/population/publications/sixbillion/sixbilpart1.pdf. Accessed 15 Feb 2018.
3. Fried, L.P., et al., Frailty in older adults: evidence for a phenotype. J Gerontol A Biol Sci Med Sci, 2001. **56**(3): p. M146-56.
4. Walston, J., et al., Research agenda for frailty in older adults: toward a better understanding of physiology and etiology: summary from the American Geriatrics Society/National Institute on Aging Research Conference on Frailty in Older Adults. J Am Geriatr Soc, 2006. **54**(6): p. 991-1001.
5. Clegg, A., et al., Frailty in elderly people. Lancet, 2013. **381**(9868): p. 752-62.
6. Harrison, J.K., et al., Managing frailty as a long-term condition. Age Ageing, 2015. **44**(5): p. 732-5.
7. Multimorbidity: clinical assessment and management (NG56). National Institute for Health and Care Excellence, September 2016. NICE, London.
8. Fit for Frailty Part 2: Developing, commissioning and managing services for people living with frailty in community settings. British Geriatrics Society & Royal College of General Practioners. 2014.
9. Clegg, A., et al., Development and validation of an electronic frailty index using routine primary care electronic health record data. Age Ageing, 2016. **45**(3): p. 353-60.
10. Davies, K., et al., Engaging the oldest old in research: lessons from the Newcastle 85+ study. BMC Geriatr, 2010. **10**: p. 64.
11. der Wiel, A.B., et al., A high response is not essential to prevent selection bias: results from the Leiden 85-plus study. J Clin Epidemiol, 2002. **55**(11): p. 1119-25.
12. Relton, C., et al., Rethinking pragmatic randomised controlled trials: introducing the "cohort multiple randomised controlled trial" design. BMJ, 2010. **340**: p. 963-967.
13. Clegg, A., et al., Improving recruitment of older people to clinical trials: use of the cohort multiple randomised controlled trial design. Age Ageing, 2015. **44**(4): p. 547-50.
14. Evans, J.R., et al., Prevalence of visual impairment in people aged 75 years and older in Britain: results from the MRC trial of assessment and management of older people in the community. Br J Ophthalmol, 2002. **86**(7): p. 795-800.
15. Electronic LogMar Vision Test, Thompson Software Soultions; http://www.thomson-software-solutions.com/test-chart-lite. Accessed 15 Feb 2018.
16. Pirozzo, S., T. Papinczak, and P. Glasziou, Whispered voice test for screening for hearing impairment in adults and children: systematic review. BMJ, 2003. **327**(7421): p. 967.
17. Nasreddine, Z.S., et al., The Montreal Cognitive Assessment, MoCA: a brief screening tool for mild cognitive impairment. J Am Geriatr Soc, 2005. **53**(4): p. 695-9.
18. Katz, J.N., et al., Can comorbidity be measured by questionnaire rather than medical record review? Med Care, 1996. **34**(1): p. 73-84.
19. Hays, R.D., C.D. Sherbourne, and R.M. Mazel, The RAND 36-Item Health Survey 1.0. Health Econ, 1993. **2**(3): p. 217-27.
20. Brazier, J., J. Roberts, and M. Deverill, The estimation of a preference-based measure of health from the SF-36. J Health Econ, 2002. **21**(2): p. 271-92.
21. EuroQoL. EQ-5D-5L. Available from: https://euroqol.org/eq-5d-instruments/eq-5d-5l-about/. Accessed 15 Feb 2018.
22. Mahoney, F.I. and D.W. Barthel, Functional Evaluation: The Barthel Index. Md State Med J, 1965. **14**: p. 61-5.
23. Lincoln, N.B. and J.R. Gladman, The Extended Activities of Daily Living scale: a further validation. Disabil Rehabil, 1992. **14**(1): p. 41-3.

For peer review only - http://bmjopen.bmj.com/site/about/guidelines.xhtml

24. Rockwood, K., et al., A global clinical measure of fitness and frailty in elderly people. CMAJ, 2005. **173**(5): p. 489-95.
25. Marshall, A., et al., Cohort differences in the levels and trajectories of frailty among older people in England. J Epidemiol Community Health, 2015. **69**(4): p. 316-21.
26. Rolfson, D.B., et al., Validity and reliability of the Edmonton Frail Scale. Age Ageing, 2006. **35**(5): p. 526-9.
27. Podsiadlo, D. and S. Richardson, The timed "Up & Go": a test of basic functional mobility for frail elderly persons. J Am Geriatr Soc, 1991. **39**(2): p. 142-8.
28. Blozik, E., et al., Geriatric Pain Measure short form: development and initial evaluation. J Am Geriatr Soc, 2007. **55**(12): p. 2045-50.
29. De Jong Gierveld, J. and T. Van Tilburg, The De Jong Gierveld short scales for emotional and social loneliness: tested on data from 7 countries in the UN generations and gender surveys. Eur J Ageing, 2010. **7**(2): p. 121-130.
30. Smith, B.W., et al., The brief resilience scale: assessing the ability to bounce back. Int J Behav Med, 2008. **15**(3): p. 194-200.
31. Luszczynska, A., U. Scholz, and R. Schwarzer, The general self-efficacy scale: multicultural validation studies. J Psychol, 2005. **139**(5): p. 439-57.
32. Yesavage, J.A., Geriatric Depression Scale. Psychopharmacol Bull, 1988. **24**(4): p. 709-11.
33. Collerton, J., et al., The Newcastle 85+ study: biological, clinical and psychosocial factors associated with healthy ageing: study protocol. BMC Geriatr, 2007. **7**: p. 14.
34. Heaven, A., et al., Keeping it credible in cohort multiple Randomised Controlled Trials: the Community Ageing Research 75+ (CARE 75+) study model of patient and public involvement and engagement. Res Involv Engagem, 2016. **2**: p. 30.
35. Department of Health, Mental Capacity Act. 2005, London, HMSO.
36. tpp. Systmone. https://www.tpp-uk.com/products/systmonline Accessed 15 Feb 2018.
37. emis health. EMIS web. https://www.emishealth.com/products/emis-web/?tab=primary-care. Accessed 15 Feb 2018.
38. MRC. Maximising the value of UK population cohorts. 2014 https://www.mrc.ac.uk/publications/browse/maximising-the-value-of-uk-population-cohorts/. Accessed 15 Feb 2018.
39. Brundle, C., et al., Convergent validity of the electronic frailty index. Age Ageing, 2018.
40. CLAHRC Yorkshire & Humber. Primary care-based management of frailty in older people. 2017 07/12/17]; Available from: http://clahrc-yh.nihr.ac.uk/our-themes/primary-care-based-management-of-frailty-in-older-people. Accessed 15 Feb 2018.

For peer review only

Domain	Measures
Sociodemographic	Age<br>Gender<br>Housing type<br>Room temperature<br>Education<br>Occupation<br>Qualifications<br>Family information<br>Formal support<br>Informal support<br>Smoking<br>Alcohol
Anthropometrics	Height<br>Weight<br>BMI<br>Bioelectric impedance analysis (BIA)
General health data	Blood pressure<br>Hearing<br>Vision<br>Comorbidities<br>Medications<br>Falls<br>Sleep
Frailty	English Longitudinal Study of Ageing Frailty Index (ELSA FI)<br>Electronic frailty index (eFI)<br>Phenotype model<br>Clinical frailty scale, 7-category version<br>Edmonton frail scale
Health-related quality of life	Short-form 36 item health questionnaire (SF36)<br>Euroqol 5-dimension health questionnaire, 5-level version (EQ5D-5L)
Cognition	Montreal cognitive assessment (MoCA)
Activities of daily living	Barthel index<br>Nottingham extended activities of daily living (NEADL)

For peer review only - http://bmjopen.bmj.com/site/about/guidelines.xhtml

Domain	Measures
Mobility	Timed-up-and-go test<br>Gait speed<br>Walking aid
Muscle strength	Grip strength
Pain	Geriatric pain measure
Loneliness	De Jong Gierveld Loneliness Scale
Depression	Geriatric depression scale
Resilience	Brief resilience scale
Self-efficacy	General self-efficacy scale

Table 1. Domains and associated measures included in CARE75+ assessment schedule

For peer review only

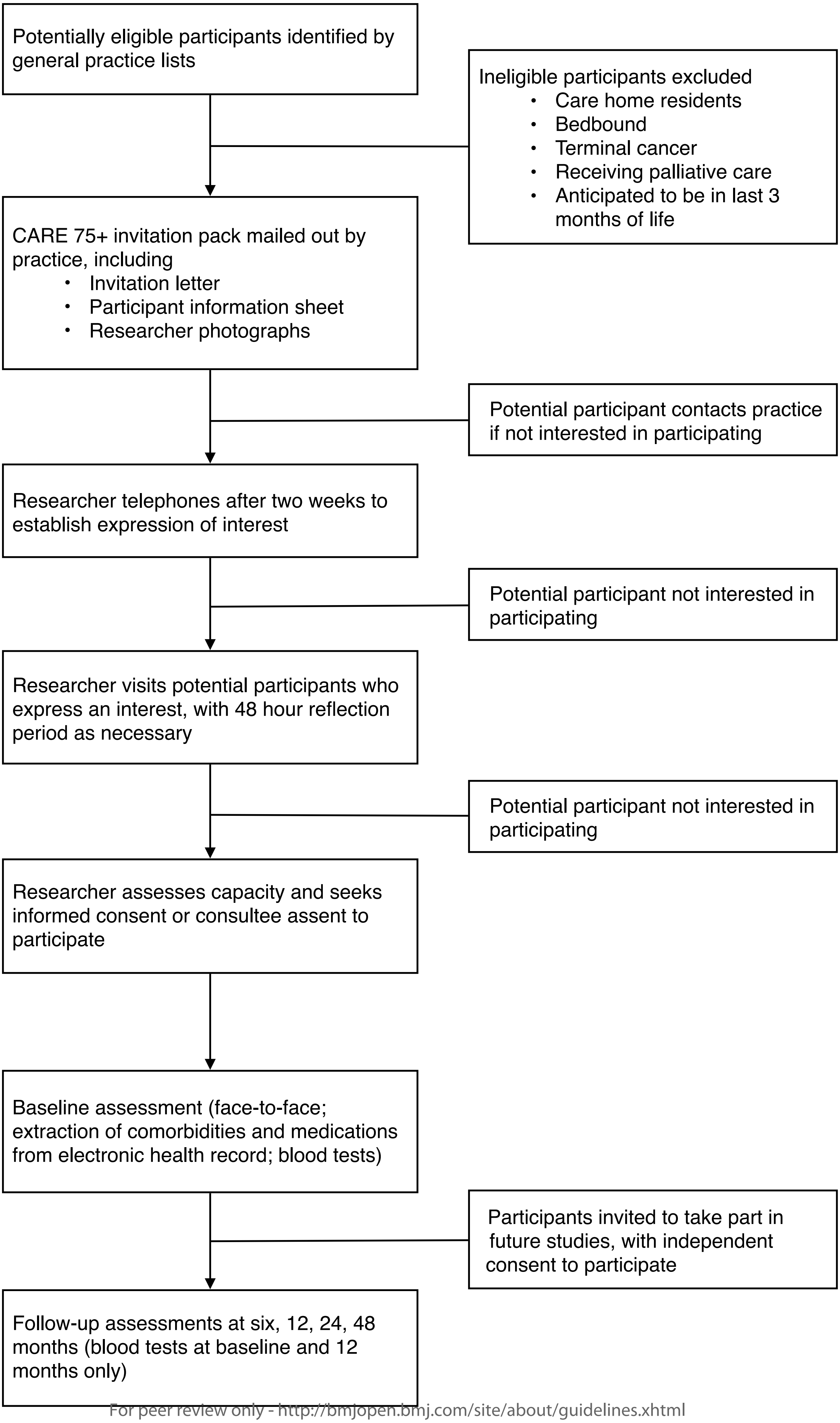

For peer review only - http://bmjopen.bmj.com/site/about/guidelines.xhtml

1. Data items are grouped in tabs
2. Please indicate which items you require using **CUT AND PASTE (plain text) TO POPULATE** the SampleRequestForm.
3. EVERY item must have a variable name to be extracted. **DO NOT** only state 'all'
3. Additions / ammendments to data requests will require the submission of a new SampleRequestForm with the additions/ammendments highlighted

NB:

Any variable with an associated 'valid' column has been checked for an acceptable level of missing data. Valid=1.

Missing data is recorded as follows:

	Not applicable will be recorded in the dataset as -95
	Unable will be recorded in the dataset as -96
	Unwilling will be recorded in the dataset as -97
	Unavailable information will be recorded in the dataset as -98
	All other missing blank fields will be -99

Required Field (please X)	Variable	Variable Label	Values	Value Label	comment
	participant_id	unique participant id for linkage			
	nhs_number				
	registration_date	date of upload to database			
	surname				
	forenames				
	title				
	address_l1				
	address_l2				
	address_l3				
	address_l4				
	address_l5				
	postcode				
	home_telephone				
	work_telephone				
	mobile_telephone				
	SixMonthFollowUpPreference	how the participant wc	1	face to face	
			2	on-line	
			3	telephone	
			9	no preference	
	EmailAddress		verabtim		

Required Field (please X)	Variable	Variable Label	Values	Value Labels	comment
	participantid	unique identification number	100000+		
	Site	unique site number	Null	Bradford Leeds	
	NHSNumber	NHS assigned number	verbatim		
	surname	participant surname	verbatim		
	forenames	participant forenames	verbatim		
	gp_code	participant's general practice	verbatim		
	status	study status of participant	0	Not contacted	
			1	Attempt contact again	
			2	Consent visit	
			99	OOS	
			100	Baseline stage, Visit {0}	
			101	Baseline stage, COMPLETE	
			200	6 month stage, Visit {0}	
			201	6 month stage, COMPLETE	
			300	12 month stage, Visit {0}	
			301	12 month stage, COMPLETE	
			400	24 month stage, Visit {0}	
			401	24 month stage, COMPLETE	
			500	48 month stage, Visit {0}	
			501	48 month stage, COMPLETE	
			600	Post 48 month stage, Visit {0}	
			601	Post 48 month stage, COMPLETE	
			999	OOS	
	StartOfStageDate	date and time that data is changed entered in nev	yyyy-mm-dd / hh/mm/ss		
	dob	participant's date of birth	yyyy/mm/dd		
	date_of_death	participant's date of death	yyyy/mm/dd		
	sex	participant's gender	1	male	
			2	female	
	ethnicity	participant's ethnic origin	1	white	
			2	mixed white /black caribbean	
			3	mixed white / asian	
			4	mixed white / black african	
			5	other mixed	
			6	black african	
			7	asian indian	
			8	asian bangladeshi	
			9	asian pakistani	
			10	other asian	
			11	black caribbean	
			12	other black	
			13	chinese	
			14	other	
	Otherethnicity		verbatim		
	MaritalStatus	participant's martial status	1	Single, that is never married	
			2	Married	
			3	Remarried	
			4	Separated but still legally married	
			5	Divorced	
			6	Widowed	
			97/98/99	missing	
	FutureStudies_ParticipantConsent	interest in taking part in future studies	1	Yes	
			2	No	
			97/98/99	missing	
			2	no	
			97/98/99	missing	
	otherStudies	are you currently participating in any other resear	1	yes	
			2	no	
			97/98/99	missing	
	otherStudiesYes	details of other studies	verbatim		

Required Field (please X)	Variable	Variable Label	Values	Value Label	comment
	HouseType	housing type	1	Bungalow	
			2	Semi-detached house	
			3	Detached house	
			4	Terraced house	
			5	Flat (which floor)	
			6	Sheltered housing (which floor)	
			7	Extra care housing	
			8	Care home (residential)	
			9	Care home (nursing)	
			97/98/99	Missing	
	Temp	temperature in c of the ro	verbatim		
	OtherRoomTemp	are all your rooms a simila	1	yes	
			2	no	
			97/98/99	missing	
	WhyNotRoomsSimilarTemp	reason for temperature di	verbatim		
	LivingCircumstances	participant's living circum	1	lives alone	
			2	lives with partner/spouse	
			3	lives with family	
			4	lives with a friend	
			5	lives with other residents of a care home	
			97/98/99	missing	
	OwnHome	Participant/husband/wife	1	Yes	
			2	No	
			97/98/99	missing	
	HowLongAtCurrentAddress	How long lived at current	Verbatim		
	GoOutAloneInDay	Go out alone in day	1	Yes	
			2	No	
			97/98/99	missing	
	GoOutAloneInNight	Go out alone in night	1	Yes	
			2	No	
			97/98/99	missing	
	HowSafeDoYouFeelDuringDay	How safe (would) feel wal	1	Very safe	NB this area=within 15 min walk
			2	Fairly safe	
			3	A bit unsafe	
			4	Very unsafe	
			97/98/99	missing	
	HowSafeDoYouFeelDuringNight	How safe (would) feel wal	1	Very safe	NB this area=within 15 min walk
			2	Fairly safe	
			3	A bit unsafe	
			4	Very unsafe	
			97/98/99	missing	
Yes value as follows as adjacent question					
1	ConcernsGettingOutNone	What concerns, if any, stop you getting out & about			
2	ConcernsGettingOutTraffic				
3	ConcernsGettingOutAntiSocialBeha		97/98/99	missing	
4	ConcernsGettingOutFalling				
5	ConcernsGettingOutCrime				
6	ConcernsGettingOutOther				
	ConcernsGettingOutOtherDetails		Verbatim		
	HowSafeDoYouFeelCrossingRoads	How safe feel crossing roa	1	Very safe	
			2	Fairly safe	
			3	A bit unsafe	
			4	Very unsafe	
			97/98/99	missing	
	HowWorriedAboutBeingVictimOfC	How worried about being	1	Very	
			2	Fairly	
			3	Not very	
			4	Not at all	
			97/98/99		

Required Field (please X)	Variable	Variable Label	Values	Value Label	comment
	ageEducation	how old were you when you finished full time continuous education '	number		
	PaidWork	what was the last paid work that you did?'	number		
	Qualifications	what was the highest educational qualification you attained?'	1	GCSE	
			2	HNS/HND	
			3	diploma	
			4	AS and A level	
			5	bachelor's degree	
			6	postgraduate	
			7	no qualifications	
			97/98/99	missing	
	VoluntaryWork	do you currently do any voluntary work?'	1	yes	
			2	no	
			97/98/99	missing	

Required Field (please X)	Variable	Variable Label	Values	Value Label	comment
	HaveBrothers	did/do you have any…	number		
	HaveSisters	did/do you have any…	97/98/99	missing	
	HaveSons	did/do you have any…			
	HaveDaughters	did/do you have any…			
	HaveGrandsons	did/do you have any…			
	HaveGranddaughters	did/do you have any…			
	BrotherFirstName	did/do you have any…	verbatim		
	SisterFirstName	did/do you have any…			
	SonFirstName	did/do you have any…			
	DaughterFirstName	did/do you have any…			
	GrandsonFirstName	did/do you have any…			
	GraddaughterFirstName	did/do you have any…			
	BrotherAliveorDead	did/do you have any…	1	alive	
	SisterAliveorDead	did/do you have any…	2	dead	
	SonAliveorDead	did/do you have any…	97/98/99	missing	
	DaughterAliveorDead	did/do you have any…			
	GrandsonAliveorDead	did/do you have any…			
	GranddaughterAliveorDead	did/do you have any…			

Required Field (please X)	Variable	Variable Label	Values	Value Label	comment
		InformalSupportLift	If you needed a lift to somewhere urgently, could you ask someone for help?'	1	Yes
		InformalSupportIll	If you wer ill in bed and needed help at home, could you ask someone for help?'	2	No
				97/98/99	Missing
		informal SupportCrisisNumberof PeopleforSupport	If you had a serious personal crisis, how many people could you turn to for comfort and support?'	number	
				97/98/99	Missing
		informalSupporPeopleLiveClose	How many of these people live within a 15-20 minute drive, if any?'	number	
				97/98/99	Missing
		InformalSupportBrother	Can you tellme the people to whom you could turn to for help (you can choose more than one)?'	1	yes
		InformalSupportSister		2	no
		InformalSupportDaughter		97/98/99	missing
		InformalSupportSon			
		InformalSupportGranddaughter			
		InformalSupportGrandson			
		InformalSupportFriend			
		InformalSupportNeighbour			
		InformalSupportOther		verbatim	
		informalSupportLastFourWeeks	approximately how many hours of informal suport have you had in the last four weeks? (unpaid)	number	
				97/98/99	Missing
		ServicesVisitWarden_ServicesVisitsFrequency	In the last 4 weeks, have you seen or had a visit from, or to, any of the following services? If so, how often?'	1	Several times a day
		ServicesVisitHomecare_ServicesVisitsFrequency		2	Once a day
		ServicesVisitNightattendentsitter_ServicesVisitsFrequency		3	One or more times a week
		ServicesVisitDaysitter_ServicesVisitsFrequency		4	Less than once a week
		ServicesVisitMealsProvision_ServicesVisitsFrequency		5	No contact
		ServicesVisitCommunityNurse_ServicesVisitsFrequency		6	Don't know
		ServicesVisitPhysiotherapist_ServicesVisitsFrequency		7	Not applicable
		ServicesVisitOccupationalTherapist_ServicesVisitsFrequency		97/98/99	Missing
		ServicesVisitChiropodist_ServicesVisitsFrequency			
		ServicesVisitSpeachTherapist_ServicesVisitsFrequency			
		ServicesVisitDietician_ServicesVisitsFrequency			
		ServicesVisitSocialWorker_ServicesVisitsFrequency			
		FormalSupportLastFourWeeks	Approximately how many hours of formal support have you had in the last four weeks?'	number	
				97/98/99	Missing
		GPVisit	In the last 4 weeks have you been to see or had a visit from a GP?'	1	Yes
		OutpatientClinic	In the last 4 weeks have you attended an outpatients clinic?'	2	No
				97/98/99	Missing
		GPVisitHowOften	number of visits	number	
		OutpatientClinicHowOften	number of visits	number	
				97/98/99	Missing
		AidAtHomeGrabRails	Do you use any of the following adi/adaptations to help you at home (>1)	1	yes
		AidAtHomeToiletSeat		2	yes
		AidAtHomeBathSeat		3	yes
		AidAtHomeReclinerChair		4	yes
		AidAtHomeBedRail		5	yes
		AidAtHomeBedRisers		6	yes
		AidAtHomeHoist		7	yes
		AidAtHomeHelpingHand		8	yes
		AidAtHomeKeySafe		9	yes
		AidAtHomePendantAlarm		10	yes
		AidAtHomeStairLift		11	yes
		AidAtHomeOutsideStep		12	yes
		AidAtHomeWheelchair		13	yes
				0	no
				97/98/99	Missing

Required Field (please X)	Variable	Variable Label	Values	Value Label	comment		
	Smoker	Have you ever smoked (this can include cigaretters, roll-ups, piopes or cigars)?					
			1	Yes			
			2	No			
			97/98/99	Missing			
	SmokeRecently	Do you smoke at all nowadays'?					
			1	Yes			
			2	No			
			97/98/99	Missing			
	HowManyCigarettes	How many cigarettes as day do y	number				
			97/98/99	Missing			
	HowMnayRollUpsOrPipes	If you smoke, or have smoked, ro	number				
			97/98/99	Missing			
	HowManyYears	Approximately how mnay years h	number				
			97/98/99	Missing			
	CALC_CigarettePackYears	Lifetime exposure					
			number				
			97/98/99	Missing			
	CALC_TobaccoPackYears	Lifetime exposure					
			number				
			97/98/99	Missing			
	drinkAlcohol	Do you ever drink alcohol incuding drinks you brew at home?'					
			1	Yes			
			2	No			
			97/98/99	Missing			
	AlcoholLast12Months	...how often have you had na alcoholoic drink of any kind during the last 12 months?'					
			1	almost every day			
			2	five or 6 days a week			
			3	three or four days a week			
			4	once or twice a week			
			5	once or twice a month			
			6	once every couple of months			
			7	once or twice a year			
			8	not at all in the last 12 months			
			9	unsure			
			97/98/99	Missing			
	AlcoholLast7Days	how many days out of the last 7	number				
			97/98/99	Missing			
	smallGlassOfWine	Thinking about the last 7 days, h	number				
	standardGlassOfWine		97/98/99	Missing			
	LargeGlassOfWine						
	lowerStrengthBeer						
	botlleOfBeer						
	canOfBeer						
	Alcopop						
	SingleSpirit						

Required Field (please X)	Variable	Variable Label	Values	Value Label	comment
	HearingAid	Do you have a hearing aid?'			
			1	Yes	
			2	No	
			3	Don't know	
			97/98/99	Missing	
	HearingAidUsage	How often do you use a hearing aid?'			
			1	Always	
			2	Regularly	
			3	Only on special occasions	
			4	Never	
			97/98/99	Missing	
	HearingTest	Have you had a hearing test in the last year?'			
			1	Yes	
			2	No	
			3	Don't know	
			97/98/99	Missing	
	DifficultyHearing	Interviewer's assessment of participant's difficulty hearing so far			
			1	No difficulty	
			2	Some difficulty	
			3	Unable to hear at all	
			97/98/99	Missing	
	HearingInQuietRoom	Do you have difficultly hearing someo	1	Yes	
			2	No	
			3	Don't know	
			97/98/99	Missing	
	HearingInLoudRoom	Do you find it difficult to follow a conv	1	Yes	
			2	No	
			3	Don't know	
			97/98/99	Missing	
	WhisperTestResult	Whisper hearing test result	1	Pass	
			2	Fail	
			97/98/99	Missing	
	GlassesOrLenses	Do you use glasses/contact lenses?'	1	Yes	
			2	No	
			3	Don't know	
			97/98/99	Missing	
	GlassesOrLensesUsedFor	purpose of glasses/lenses	1	Distance	
			2	Reading	
			3	Both distance & reading	
			4	Don't know	
			97/98/99	Missing	
	EyesightTested	...eyesight tested by an optician in the	1	Yes	
			2	No	
			3	Don't know	
			97/98/99	Missing	
	RecognisingPeople	....difficulty recognisign a friend across	1	Yes	
			2	No	
			3	Don't know	
			97/98/99	Missing	
	ReadingNewsprint	...difficulty reading ordinary newsprint	1	Yes	
			2	No	
			3	Don't know	
			97/98/99	Missing	
	BlindorPartiallySighted	Registered blind or partially sighted	1	Yes	
			2	No	
			3	Don't know	
			97/98/99	Missing	
	LeftEye	(no pinholes) is the participant able to	1	Yes	
			2	No- form vision only	
			3	No- no vision	
			97/98/99	Missing	
	LeftEyeYesMarginLabel	logMAR margin of the last line read co	number		
			97/98/99	Missing	
	LeftEyeYesNumLettersMissed	total number of letters missed or incor	number		
			97/98/99		
	RightEye	(no pinholes) is the participant able to	1	Yes	
			2	No- form vision only	
			3	No- no vision	
			97/98/99	Missing	
	RightEyeYesMarginLabel	logMAR margin of the last line read co	number		
			97/98/99	Missing	
	RightEyeYesNumLettersMissed	total number of letters missed or incor	number		
			97/98/99		
	LeftEyePinholes	(with pinholes) is the participant able	1	Yes	
			2	No- form vision only	
			3	No- no vision	
			97/98/99	Missing	
	LeftEyePinholesYesMarginLabel	logMAR margin of the last line read co	number		
			97/98/99	Missing	
	LeftEyePinholesYesNumLettersMissed	total number of letters missed or incor	number		
			97/98/99		
	RightEyePinholes	(with pinholes) is the participant able	1	Yes	
			2	No- form vision only	
			3	No- no vision	
			97/98/99	Missing	
	RightEyePinholesYesMarginLabel	logMAR margin of the last line read co	number		
			97/98/99	Missing	
	RightEyePinholesYesNumLettersMissed	total number of letters missed or incor	number		
			97/98/99		
	CALC_LogMARLeftEye	no pinhole score	number		
			97/98/99		
	CALC_LogMARRightEye	no pinhole score	number		
			97/98/99		
	CALC_LogMARLeftEyePinholes	with pinholes score	number		
			97/98/99		
	CALC_LogMARRightEyePinholes	with pinholes	number		
			97/98/99		

Required Field (please X)	Variable	Variable Label	Values	Value Label	comment
	SF-36HealthSurvey	In general how would you say your health is?'	1	Excellent	
			2	Very Good	
			3	Good	
			4	Fair	
			5	Poor	
			97/98/99	Missing	
	healthComparedToAYearAgo	Compared to one year ago, how would your rate your health in general now?	1	Much better now than one year ago	
			2	Somewhat better now than one year ago	
			3	About the same as one year ago	
			4	Somewhat worse now than one year ago	
			5	Much worse now than one year ago	
			97/98/99	Missing	
	VigorousActivities_SF36Activities	...does you health now limit you in these activities? If so how much?'	1	Limited a lot	
	ModerateActivities_SF36Activities		2	Limited a little	
	LiftingOrCarryingActivities_SF36Activities		3	Not limited at all	
	ClimbingSeveralStairsActivities_SF36Activities		97/98/99	Missing	
	ClimbingStairsActivities_SF36Activities				
	BendingKneelingStoopingActivities_SF36Activities				
	WalkingMoreThanAMile_SF36Activities				
	WalkingSeveralBlocksActivities_SF36Activities				
	WalkingOneBlockActivities_SF36Activities				
	BathingOrDressingYourself_SF36Activities				
	HealthIssuesReducedTimeSpentOnWork	During the past 4 weeks, have you had any of the following problems with your work	1	Yes	
	HealthIssuesAccomplishedLess		2	No	
	HealthIssuesLimitedOtherActivities		97/98/99	Missing	
	HealthIssuesDifficultiesPerformingWork				
	EmotionalIssuesReducedTimeSpentOnWork	During the past 4 weeks, have you had any of the following problems with your work	1	Yes	
	EmotionalIssuesAccomplishedLess		2	No	
	EmotionalIssuesDidntDoWorkAsCarefully		97/98/99	Missing	
	HealthInterferedActivities	During the past 4 weeks, to what extent has your physical health or emotional proble	1	Not at all	
			2	Slightly	
			3	Moderately	
			4	Quite a bit	
			5	Extremely	
			97/98/99	Missing	
	BodilyPain	How much bodily pain have you had during the past 4 weeks?'	1	None	
			2	Very mild	
			3	Mild	
			4	Moderate	
			5	Severe	
			6	Very severe	
			97/98/99	Missing	
	PainInterfereWithWork	During the past 4 weeks, how much did pain interfere with your normal work (includi	1	Not at all	
			2	A little bit	
			3	Moderately	
			4	Quite a bit	
			5	Extremely	
			97/98/99	Missing	
	FeelFullOfPep_FeelingResponses	How much time during the past 4 weeks have/did you ...?'	1	All of the Time	
	FeelNervous_FeelingResponses		2	Most of the Time	
	FeelDown_FeelingResponses		3	A Good Bit of the Time	
	FeltCalm_FeelingResponses		4	Some of the Time	
	ALotOfEnergy_FeelingResponses		5	A Little of the Time	
	FeltDownhearted_FeelingResponses		6	None of the Time	
	FeelWornOut_FeelingResponses		97/98/99	Missing	
	BeenHappy_FeelingResponses				
	FeelTired_FeelingResponses				
	HowOftenHealthInterferedActivities	During the past 4 weeks, how much of the time has your physical health or emotional	1	All of the time	
			2	Most of the time	
			3	Some of the time	
			4	A little of the time	
			5	None of the time	
			97/98/99	Missing	
	SickEasier_HealthExpectation	How True or False is each of the following statements for you?'	1	Definitely True	
	HealthyasAnybody_HealthExpectation		2	Mostly True	
	HealthToGetWorse_HealthExpectation		3	Don't Know	
	HealthIsExcellent_HealthExpectation		4	Mostly False	
			5	Definitely False	
			97/98/99	Missing	
	CALC_AvgSF36PhysicalFunctioning	total SF36 score for each domain	number		
	CALC_AvgSF36RoleLimitationPhysicalHealth				
	CALC_AvgSF36RoleLimitationEmotionalProblems				
	CALC_AvgSF36EnergyOrFatigue				
	CALC_AvgSF36EmotionalWellbeing				
	CALC_AvgSF36SocialFunctioning				
	CALC_AvgSF36Pain				
	CALC_AvgSF36GeneralHealth				
	CALC_AvgSF36HealthChange				

Required Field (please X)	Variable	Variable Label	Values	Value Label	comment
	MoCAlternatingTrailMaking	number letter sequencing	1	correct	
	MoCAVisuoconsturctinalCube	completion of 3D cube	2	incorrect	
	MoCAVisuoconsturctinalContour	drawing of clock face outline	97/98/99	Missing	
	MoCAVisuoconsturctinalHands	placement of clock hands			
	MoCAVisuoconsturctinalNumbers	placement of clock numbers			
	MocANamingLion		1	correct	
	MocANamingRhino		2	incorrect	
	MocANamingCamel		97/98/99	Missing	
	MoCADigitsBackward	repetition of digits forward	1	correct	
	MoCADigitsForward	repetition of digits backwards	2	incorrect	
			97/98/99	Missing	
	MoCAVigalence	recognition of 'A' in letter sequence	1	0-1 error	
			2	>1 error	
			97/98/99	Missing	
	MoCASerial	number of correct subtractions of 7 starting	0	correct subtractions	
			1		
			2		
			3		
			4		
			5		
			97/98/99	Missing	
	MoCASentenceRepetitionOne	repeat sentence	1	correct	
	MoCASentenceRepetitionTwo	repeat sentence	2	incorrect	
			97/98/99	Missing	
	MoCAVerbalFluency	tell me as many words as you can beginning	number		
			97/98/99	Missing	
	MoCAAbstractionOne	what is the likeness of train and bicycle?	1	correct	
	MoCAAbstractionTwo	what is the likeness of ruler and watch	2	incorrect	
			97/98/99	Missing	
	MoCADelayedRecallFace	recall of words read earlier	1	recalled	
	MoCADelayedRecallVelvet		2	not recalled	
	MoCADelayedRecallChurch		3	incorrect	
	MoCADelayedRecallDaisy		97/98/99	Missing	
	MoCADelayedRecallRed				
	MoCAOrientationDate	orientation to time and place	1	correct	
	MoCAOrientationMonth		2	incorrect	
	MoCAOrientationYear		97/98/99	Missing	
	MoCAOrientationDay				
	MoCAOrientationPlace				
	MoCAOrientationCity				
	MoCAOrientationEducation	years of formal education	1	<13	
			2	>=13	
			97/98/99	Missing	
	CALC_MoCA	score out of 30 >=26 'normal'	0-30		
			97/98/99	Missing	

Required Field (please X)	Variable	Variable Label	Values	Value Label comment
	MyocardialInfarction	Have you ever had a heart attack	1	yes
	CongestiveHeartFailure	Have you ever been treated for heart failure?	2	no
	PeripheralVascularDisease	Have you had an operation to unclog or bypass the art	3	not sure
	CerebrovascularAccident	Have you had a stroke, cerebrovascular accident, bloo	97/98/99	Missing
	Hemiplegia	Do you have difficulty moving an arm or leg as a result of the stroke or cerebrovascular accident?		
	Asthma	Do you have asthma?		
	AsthmaMedicines	Do you take medications for your asthma?'	2	no
			1	yes, only with flare ups of my asthma
			3	yes, I take medicine regularly even when I'm not having a flare up
			97/98/99	Missing
	emphysema	Do you have emphysema, chronic bronchitis, or chron	1	yes
			2	no
			3	not sure
			97/98/99	Missing
	emphysemaMedicines	Do you take medicines for your lung disease?'	2	no
			1	yes, only with flare ups
			3	yes, I take medicine regularly even when I'm not having a flare up
			97/98/99	Missing
	UlcerDisease	Do you have stomach ulcers or peptic ulcer disease diagnosed by endoscopy or upper gi or barium swallow study?		
	HasDiabetes	Do you have diabetes?	1	Yes, treated by modifying my diet
			2	No
			3	Yes, treated by medications taken by mouth
			4	Yes, treated by insulin
			97/98/99	Missing
	DiabetesCausedProblems	Has the diabetes caused any of the following problem:	1	Problems with kidneys
			2	Problems with eyes, treated by an ophthalmologist
			3	unsure
			97/98/99	Missing
	ProblemsWithKidneys	Have you ever had problems with your kidneys?'	1	Poor kidney function (blood tests show high creatinine)
			2	No
			3	Have used hemodialysis or peritoneal dialysis
			4	Have received kidney transplantation
			97/98/99	Missing
	ConnectiveTissueDisese	Do you have Rheumatoid Arthritis?	1	Yes
			2	No
			3	Not sure
			97/98/99	Missing
	ConnectiveTissueDiseaseMedications	Do you take medications for it regularly?'	1	Yes
			2	No
			3	Not sure
			97/98/99	Missing
	ConnectiveTissueDiseaseType	do you have....?'	1	Lupua
			2	Polymyalgia rheumatica
			3	neither lupus nor polymalgia
			4	not sure
			97/98/99	Missing
	Dementia	Do you have any of the following conditions..?	1	Yes
	Cirrhosis		2	No
	Leukemia		3	Not sure
	Lymphoma		97/98/99	Missing
	Aids			
	Cancer			
		Has the cancer spread or metastasized to other parts (	1	Yes
	CancerSpread		2	No
			3	Not sure
			97/98/99	Missing
	CALC_KatzCormbidity	total score plus age based on the Charlson index scorii	number	
	Hypertension	Does the participant's GP record identify...	1	Yes
	1b. Any atherosclerotic disease		2	No
	1c. Ischaemic heart disease		3	Not sure
	Any atherosclerotic disease		2	No
	Ischaemic heart disease		97/98/99	Missing
	Cerebrovascular disease			
	Peripheral vascular disease			
	Heart failure			
	Atrial fibrillation			
	Atrial flutter			
	Osteoarthritis			
	Cervical or lumbar spondylosis			
	Rheumatoid arthritis			
	Other arthritis (specified)			
	Arthritis (type not specified)			
	Joint replacement			
	Cataract			
	Cataract surgery			
	Age related macular degeneration			
	Glaucoma Diabetic eye disease			
	Registered blind			
	Registered partially sighted			
	Any cancer			
	Any cancer, excluding nonmelanoma skin ca			
	Any cancer <5 years since diagnosis			
	Chronic obstructive pulmonary disease (COP			
	Asthma			
	Other respiratory disease			
	Diabetes mellitus			
	Hypothyroidism			
	Hyperthyroidism			
	Dementia			
	Parkinson's disease			
	Anxiety			
	Depression			
	Anaemia: WHO criteria for haemoglobin con			
	Anaemia: Joosten's criterion for haemoglobi			
	Osteoporosis			
	Fractured hip, wrist or backbone			
	Renal function using modification of diet in r		1	Normal/mildly reduced
			2	Moderately reduced (stage 3††)
			3	Severely reduced (Stage 4††)
			4	Very severely reduced (stage 5††)
			97/98/99	Missing
	Calculate total number of diseases	Total number of diseases from GP record (excluding os	Number	
			97/98/99	Missing

Required Field (please X)	Variable	Variable Label	Values	Value Label	comment
	FallsInLast12Months	in the last 12 months have you had a fall	1	Yes	
			2	no	
			3	not sure	
			97/98/99	missing	
	HowManyFalls	How mnay time s have you fallen in the l	number		
			97/98/99	missing	
	FallsFractures	...have you broken any bones/had and fra	1	Yes	
			2	no	
			3	not sure	
			97/98/99	missing	
	FallsBrokenBones	How many times has a fall resulted in a b	number		
			97/98/99	missing	
	FallsAccidentEmergency	...did you go to Aand E follwing a fall?	1	Yes	
			2	no	
			3	not sure	
			97/98/99	missing	
	FallsGetAandE	How many times did you attend A&E	number		
			97/98/99	missing	
	FallsStayOvernight	...were you admitted to a hospital followi	1	Yes	
			2	no	
			3	not sure	
			97/98/99	missing	
	FallsGetAdmited	How many times were you admitted to a	number		
			97/98/99	missing	
	FallsSeenGP	Have you (or your carer) ever seen your (	1	Yes	
			2	no	
			3	not sure	
			97/98/99	missing	
	FallsSpecialist	Have you ever seen a falls specialist?'	1	Yes	
			2	no	
			3	not sure	
			97/98/99	missing	
	LossOfConfidence	Have your falls caused any of the followi	1	Yes	
	WorryAboutFalling		2	no	
	GoingOutLessOften		3	not sure	
	IncreaseCareReceive		97/98/99	missing	

Required Field (please X)	Variable	Variable Label	Values	Value Label	comment
	TookPresMed	Number of prescribed medications	number		
			97/98/99	Missing	
	Medication*_MedicationName	Name of prescribed medication 1-30	verbatim		
			97/98/99	Missing	
	Medication*_Dosage	Dosage of prescribed medicationsverbatim	number		
			97/98/99	Missing	
	Medication*_Frequency	Frequency of prescribed medication	number		
			97/98/99	Missing	
	**TookNonPresMed**	Number of non-prescribed medicines, vitamin or mineral supplement	number		
			97/98/99	Missing	
	**NonPresMedication*_NonPresMedicationName**	Name of non-prescribed medication 1-30	verbatim		
			97/98/99	Missing	
	**NonPresMedication*_NonPresMedicationDosage**	Dosage of non-prescribed medicationsverbatim	number		
			97/98/99	Missing	
	**NonPresMedication*_NonPresMedicationFrequency**	Frequency of non-prescribed medication	number		
			97/98/99	Missing	
	**COUNT_PrescribedMedication**		number		
	**COUNT_NonPrescribedMedication**		number		

Required Field (please X)	Variable	Variable Label	Values	Value Label	comment
	WalkAroundOutside_NEADLPartOne	...what have you *actually* done in the l	0	not at all	
	ClimbStairs_NEADLPartOne		1	with help	
	GetInAndOutOfCar_NEADLPartOne		2	on your own with difficulty	
	WalkOverUnevenGround_NEADLPartOne		3	on your own	
	CossRoads_NEADLPartOne		97/98/99	Missing	
	TravelOnPublicTransport_NEADLPartOne				
	ManageToFeedYourself_NEADLPartOne				
	MakeYourselfAHotDrink_NEADLPartOne				
	TakeHotDrinksFromOneDrinksToAnother_NEADLPartOne				
	DoTheWashingUp_NEADLPartOne				
	MakeYourselfAHotSnack_NEADLPartOne				
	ManageOwnMoney_NEADLPartTwo	...what have you *actually* done in the l	0	no	
	WashItemsOfClothing_NEADLPartTwo		1	with help	
	DoOwnHousework_NEADLPartTwo		2	on your own with difficulty	
	DoOwnShopping_NEADLPartTwo		3	on your own	
	DoFullClothesWash_NEADLPartTwo		97/98/99	Missing	
	ReadNewspapersOrBooks_NEADLPartTwo				
	UseTelephone_NEADLPartTwo				
	WriteLetters_NEADLPartTwo				
	GoOutSocially_NEADLPartTwo				
	ManageGarden_NEADLPartTwo				
	Drive_NEADLPartTwo				
	CALC_NEADL	Total NEADL. Higher score = more ind	number 0-66		
			97/98/99	Missing	
	Feeding	Do you have any difficulty with the fol	0	uanble	
			1	needs help cutting, spreading butter or requires modified diet	
			2	independent	
			97/98/99	Missing	
	Bathing		0	dependent	
			1	independent (or in shower)	
			97/98/99	Missing	
	Grooming		0	needs help with personal care	
			1	independent face/hair/teeth/shaving	
			97/98/99	Missing	
	Dressing		0	dependent	
			1	needs help but can do about half unaided	
			2	independent including buttons and zips	
			97/98/99	Missing	
	Bowels		0	incontinent or needs enemas	
			1	occasional accident	
			2	continent	
			97/98/99	Missing	
	Bladder		0	unable (or catherterised and unable to manage alone)	
			1	occasional accident	
			2	continent	
			97/98/99	Missing	
	ToiletUse		0	dependent	
			1	needs some help but can so somethings alone	
			2	independent (on/off an wiping)	
			97/98/99	Missing	
	Transfers		0	unable, no sitting balance	
			1	major help (one or two people physical) can sit up	
			2	minor help (verbal or physical)	
			3	independent	
			97/98/99	Missing	
	Mobility		0	immobile or <50 yards	
			1	wheelchair independent including corners >50 yards	
			2	walks with the help of one person (verbal or physical) >50 yards	
			3	independent but may use any walking aide >50 yards	
			97/98/99	Missing	
	Stairs		0	unable	
			1	needs help (verbal physical, carrying aid)	
			2	independent	
			97/98/99	Missing	
	CALC_Barthel_Index	Total BARTHEL. Higher score means m	number 0-20		
			97/98/99	Missing	

Required Field (please X)	Variable	Variable Label	Values	Value Label	comment
	HeightWeighttimeofDay	time measurements are taken	hh.mm		
	Demispan	from middel of collar bone to index finger	number cm		
	DemispanHeight	Calculation of height from demispan	number cm		
	Height		number cm		
	Weight		number kg		
	BodyFatPercentage		number %		
	FatMass		number kg		
	FatFreeMass		number kg		
	BodyWaterPercentage		number %		
	MuscleMass		number kg		
	BoneMass		number kg		
	CALC_DemiSpanHeight				
	CALC_BMI	mass (kg) /demi span height (m)2	number		
	WeightLoss	In the last year, have you lost more than 10lb	1	yes	
			2	no	
			3	not sure	
			97/98/99	Missing	
	SittingBloodPresssure		yes	1	
			no	2	
			97/98/99	Missing	
	ReasonNoSittingBloodPressure		verbatim		
			1	Participant refused	
			3	Cuff the wrong size	
			4	Monitor malfunction	
			5	StandingBloodPressureTaken	
	SittingSystolic	BP reading	number mm Hg		
	Sitting Diastolic	BP reading	97/98/99	Missing	
	SittingPulse	pulse			
	StandingBloodPresssure		yes	1	
			no	2	
			97/98/99	Missing	
	ReasonNoStandingBloodPressure		verbatim		
	StandingSystolic	BP reading	number mm Hg		
	Standing Diastolic	BP reading	97/98/99	Missing	
	StandingPulse	pulse			
	DominantHand	Which is the dominant hand?	1	right	
			2	left	
	DominantHandFirstAttempt	grip strength in dominant hand	number kPA		
	DominantHandSecondAttempt				
	DominantHandThirdAttempt				
	NondominantHandFirstAttempt	grip strength in passive hand	number kPA		
	NondominantHandSecondAttempt				
	NondominantHandThirdAttempt				
	CALC_DominantMeanGripStrength	mean grip strength dominant hand	number kPA		
	CAL_NonDominantMeanGripStrength	mean grip strength passive hand			

Required Field (please X)	Variable	Variable Label	Values	Value Label	comment
	WalkTime	over a 3 metre distance	number seconds		
			97/98/99	Missing	
	ExhaustionPartA	How often in the last week did you feel 'everything I did was an effort?'	0	rarely or none of the time	
			1	some or a little of the time	
			2	moderate amount of the time	
			3	most of the time	
			97/98/99	Missing	
	ExhaustionPartB	How often in the last week did you feel 'I could not get going'?'	0	rarely or none of the time	
			1	some or a little of the time	
			2	moderate amount of the time	
			3	most of the time	
			97/98/99	Missing	
	TUGTSkipped	was the TUGT missed out?	1	yes	
			2	no	
	TUGNotDoneReason	reason for skipping TUGT	verbatim		
	chairUsed		1	yes	
			2	no	
	ChairHeight	height of the chair used to stand up from	number mm		
	AbleToGetUpFromChair		1	yes	
			2	no	
			97/98/99	Missing	
	TimeToGetUp	time taken to complete in seconds	number secs.		
			97/98/99	Missing	
	**WalkingAidUsed**	walking aid used to complete the TUGT	1	Independent	
			2	1x walking stick	
			3	2x walking sticks	
			4	Walking Zimmer frame	
			5	Wheeled Zimmer frame	
			6	3-wheeled walker	
			7	4-wheeled walker	
			8	Kitchen trolley	
			97/98/99	Missing	

Required Field (please X)	Variable	Variable Label	Values	Value Label comment
	ClinicalFrailtyResearcher	Researcher frailty assessment based on observatic	1	very fit; robust, active, energetic, well motivated and fit. These people commonly exercise regularlry and are in the most fit group for their age
			2	well; without active disease, but less fit htan people in category 1
			3	well; with terated comorbid disease. Disease symptoms are controlled compared with those in category 4
			4	apparently vulnerable; although not frankly dependent, these people commonly complain of beign 'slowed up' or have disease symptoms
			5	mildly frail; with limited dependence on others for instrumental activities of daily living
			6	moderately frail; help is needed with both instrumental and non-instrumental activities of daily living
			7	severely frail; completely dependent on others for the activities of daily living or terminally ill
			99	missing
	**ClinicalFrailtyIsClinicianPresent**	Is a registrar present to complete 2nd part of the a	1	yes
			2	no
	**ClinicalFrailtyClinican**	Clinician frailty assessment based on observation	1	very fit; robust, active, energetic, well motivated and fit. These people commonly exercise regularlry and are in the most fit group for their age
			2	well; without active disease, but less fit htan people in category 1
			3	well; with terated comorbid disease. Disease symptoms are controlled compared with those in category 4
			4	apparently vulnerable; although not frankly dependent, these people commonly complain of beign 'slowed up' or have disease symptoms
			5	mildly frail; with limited dependence on others for instrumental activities of daily living
			6	moderately frail; help is needed with both instrumental and non-instrumental activities of daily living
			7	severely frail; completely dependent on others for the activities of daily living or terminally ill
			99	missing
	CALC_FriedTotalScore	Fried frailty score	0	not frail
			1	pre frail
			2	pre frail
			3	mild frailty
			4	moderate frailty
			5	severe frailty
			97/98/99	missing
	PenFromHand	Can you take this oen from my hand if I hold it her	1	yes
			2	no
			97/98/99	missing
	BustrainTimetable	Are you able to use a bus or train timetable?'	1	yes
			2	no
			97/98/99	missing
	SitUprightforTwoHours	Are you able to sit upright in a chair for two hours'	1	yes
			2	no
	CoinFromTable	Are you able to pick up a 10p coin from a table?'	1	yes
			2	no
			97/98/99	missing
	EFSCognition	draw 10 past 11 on clock face	0	no errors
			1	minor spacing errors
			2	other errors
			97/98/99	missing
	EFSAdmitted	How many hospital admissions in the past year	0	0
			1	1 or 2
			2	>2
			97/98/99	missing
	EFSSocialSupport	can you count on someone?	0	always
			1	sometimes
			2	never
			97/98/99	missing
	EFSForgetMedication	At times do you forget to take your prescription m	0	no
			1	yes
			97/98/99	missing
	EFSNutrition	Have you lost weight and clothes become loose?	0	no
			1	yes
			97/98/99	missing
	EFSMood	Do you often feel sad or depressed?'	0	no
			1	yes
			97/98/99	missing
	EFSContinence	Do you have a problem losing control of urine whe	0	no
			1	yes
			97/98/99	missing
	CALC_EdmontonFrailScale	total score out of 17	0-5	not frail
			6-7	vulnerable
			8-9	mild frailty
			10-11	moderate frailty
			12-17	severe frailty
			97/98/99	missing
	EFIGPScore	data extracted from GP summary records		
	Fit (eFI score 0 - 0.12)	: People who have no or few long-term conditions that are usually well controlled. This group would mainly be independent in day to day living activities.		
	Mild frailty (eFI score 0.13 – 0.24)	: People who are slowing up in older age and may need help with personal activities of daily living such as finances, shopping, transportation.		
	Moderate Frailty (eFI score 0.25 – 0.36)	: People who have difficulties with outdoor activities and may have mobility problems or require help with activites such as washing and dressing.		
	Severe Frailty (eFI score > 0.36)	: People who are often dependent for personal cares and have a range of long-term conditions/multimorbidity. Some of this group may be medically stable but others can be unstable and at risk of dying within 6 - 12 months		
	99	missing		
	CALC_ELSAFrailtyIndex			
	0-10	very fit		
	11-14	well		
	15-24	vulnerable		
	>=25	frail		
	missing 99			
	http://www.elsa-project.ac.uk/publicationDetails/id/7167			

Required Field (please X)	Variable	Variable La	Values	Value Label	comment
	Sleep	Have you sl	1	Yes	
			2	No	
			3	Not sure	
			97/98/99	Missing	
	HealthRelatedMobility	EQ5D	1	no problems walking about	
			2	slight problems	
			3	moderate problems	
			4	severe problems	
			5	unable to walk	
			97/98/99	Missing	
	HealthRelatedSelfCare		1	no problems washing or dressing	
			2	slight problems	
			3	moderate problems	
			4	severe problems	
			5	unable to wash or dress	
			97/98/99	Missing	
	HealthRelatedUsualActivities		1	no problems doing usual activities	
			2	slight problems	
			3	moderate problems	
			4	severe problems	
			5	unable to do usual activities	
			97/98/99	Missing	
	HealthRelatedPain		1	no pain or discomfort	
			2	slight pain or discomfort	
			3	moderate pain or discomfort	
			4	severe pain or discomfort	
			5	extreme pain or discomfort	
			97/98/99	Missing	
	HealthRelatedAnxiety		1	not anxious or depressed	
			2	slightly anxious or depressed	
			3	moderately anxious or depressed	
			4	severely anxious or depressed	
			5	extremely anxious or depressed	
			97/98/99	Missing	
	CALC_EQ5D5L	total EQ5D score			

Required Field (please X)	Variable	Variable Label	Values	Value Label	comment
	**PainStoppedActivity**	has pain…	0	no	
	**PainStoppedClimbing**		2	yes	
	**PainStoppedWalkingMoreThan200**		97/98/99	Missing	
	**PainStoppedWalkingLessThan200**				
	**PainCutDownActivities**				
	**PainAccomplishingLess**				
	**PainLimitedWork**				
	**PainWorkRequireExtraEffort**				
	**PainTroubleSleeping**				
	**PainPreventSocialActivities**				
	**PainToday**	on a scale of 0-10 with 0	number 0-10		
	**PainLast7Days**		97/98/99	Missing	
	**CALC_GeriatricPainMeasure**	Geriatric Pain Measure to	number 0-40		
			97/98/99	Missing	

Required Field (please X)	Variable	Variable Label	Values	Value Label	comment		
	SomeoneToTalkTo_LoninessScale	...the extent to which the statements apply	1	yes			
	MissHavingCloseFriend_LoninessScale		2	more or less			
	SenseOfEmptiness_LoninessScale		3	no			
	PeopleICanLeanOn_LoninessScale		97/98/99	missing			
	MissCompanyOfOthers_LoninessScale						
	CircleOfFriendsLimited_LoninessScale						
	PeopleITrustCompletely_LoninessScale						
	PeopleIFeelCloseTo_LoninessScale						
	MissHavingPeopleAround_LoninessScale						
	FeelRejected_LoninessScale						
	CallFriendsWheneverINeed_LoninessScal						
	CALC_emotionalLoneliness	yes or more or less on ? 2,3,5,6,9,10	number				
	CALC_missingemotionalLoneliness	number of mising items					
	CALC_socialLoneliness	no or more or less on ?1,4,7,8,11					
	CALC_misssingsocialLoneliness	number of missing items					
	CALC_totalLonliness	De Jong Gierveld scale sum of emotional and social loneliness	0-2	not lonely			
			3-8	moderately lonely			
			9-10	severely lonely			
			11	very serverly lonely			
			97/98/99	Missing			
	CALC_LonelinessScore	loneliness category	1	Not lonely			
			2	Moderately lonely			
			3	Severely lonely			
			4	Very severely lonely			
			97/98/99	Missing			
1	SatisfiedWithLife	basically satisfied with life	0	yes		1	yes
2	DroppedActivities	dropped many activities and interests	1	no		0	no
3	FeelEmpty	feel life is empty	97/98/99	Missing		97/98/99	Missing
4	GetBored	often get bored					
5	GetBored2 -?goodspirits?	in good spirits most of the time					
6	SomethingBadGoingToHappen	afraid that something bad is going to happen					
7	FeelHappy	feel happy most of the time					
8	FeelHelpless	often feel helpless					
9	PreferToStayAtHome	prefer to stay at home rather than going out and doing things					
10	ProblemsWithMemory	more problems with memory than most					
11	WonderfulToBeAlive	think it's wonderful to be alive					
12	Worthless	worthless the way you are now					
13	FullOfEnergy	full of energy					
14	SituationIsHopeless	situation is hopeless					
15	BetterOffThanYou	most people better off than you					
	GeriatricDepressionScale	total from 15 questions reverse score for items 1,5,7,11,13 >=5 suggests depressed	number 0-15				
			97/98/99	Missing			

Required Field (please X)	Variable	Variable Label	Values	Value Label	comment
	BounceBackQuickly_BriefResilienceScale	agreement with resiliance	1	strongly agree	
	HardTimeThroughStressfulEvents_BriefResilienceScale		2	agree	
	LongToRecoverFromStress_BriefResilienceScale		3	neutral	
	SnapBackFromSomethingBad_BriefResilienceScale		4	disagree	
	DifficultTimesLittleTrouble_BriefResilienceScale		5	strongly disagree	
	TimeToGetOverSetBacks_BriefResilienceScale		97/98/99	Missing	
	CALC_briefResilianceScale	sum/6 items reverse scorir	number 1-6 higer=more resiliant		
			97/98/99	Missing	
	SolveDifficultProblems_SelfEfficacyScale	agreement with general se	1	not at all true	
	WaysToGetWhatIWant_SelfEfficacyScale		2	hardly true	
	AccomplishMyGoals_SelfEfficacyScale		3	moderately true	
	DealWithUnexpectedEvents_SelfEfficacyScale		4	exactly true	
	HandleUnforeseenSituations_SelfEfficacyScale		97/98/99	Missing	
	SolveMostProblems_SelfEfficacyScale				
	RemainCalm_SelfEfficacyScale				
	FindSeveralSolutions_SelfEfficacyScale				
	ThinkOfASolution_SelfEfficacyScale				
	HandleWhateverComes_SelfEfficacyScale				
	CALC_SelfEfficacyScale	Total self-efficacy score- hi	number 10-40		
			97/98/99	Missing	

NB: Individual items in ELSA are pulled from other assessm
WalkingOneBlock_SF-36Activities
SitUprightForTwoHours
AbleToGetUpFromChair
ClimbingSeveralStairs_SF-36Activities
ClimbingStairs_SF-36Activities
BendingKneelingStooping_SF-36Activities
PenFromHand
ModerateActivities_SF-36Activities
VigorousActivities_SF-36Activities
CoinFromTable
HealthRelatedSelfCare
HealthRelatedMobility
Bathing
Feeding
Transfers
ToiletUse
BustrainTimetable
MakeYourslefAHotSnack
DoOwnShopping_NEADLPartTwo
UseTelephone_NEADLPartTwo
EFSForgetMedication
ManageOwnMoney_NEADLPartTwo
DoOwnHousework_NEADLPartTwo
ExhaustionPartA
Sleep
GoodSpirits
WonderfulToBeAlive
FeelHappy
ExhaustionPartB
Hypertension
Atherosclerotic
MyocardialInfarction
HeartFailure
DiabetesMellitus
Cerebrovascular
COPD
RespiratoryAsthma
Osteoporosis
ExcludingNonmelanoma
Parkinson
NeurologicalDementia
eyesight 4 & 5
hearing & vision 6 & 7
FallsInLast12Months
FracturedHipWristBackbone
Joint
geriatric pain measure 3 or
MoCAOrientationDate
MoCAOrientationMonth
MoCAOrientationYear
MoCAOrientationDay
MoCASentenceRepetitionOne
MoCAVerbalFluency
delayed recall combines 5 questions
SF-36HealthSurvey'
GeriatricDepressionScale >= 5
CALC_totalLonliness) >= 3
AtrialFibrillation
AtrialFlutter
Osteoarthritis
RheumatoidArthritis
OtherArthritis
Arthritis
Anxiety
Depression
RecognisingPeople
ReadingNewsprint
HearingInQuietRoom
HearingInLoudRoom
PainStoppedWalkingMoreThan200
PainStoppedWalkingLessThan200
MoCADelayedRecallFace
MoCADelayedRecallVelvet
MoCADelayedRecallChurch
MoCADelayedRecallDaisy
MoCADelayedRecallRed
CALC_ELSAFrailtyIndex

NB: Individual items in Fried are pulled from other assessment data where possible to avoid overburdening the participant								
CALC_DemiSpanHeight								
CALC_BMI								
WalkTime								
WeightLoss								
ExhaustionPartA								
ExhaustionPartB								
DominantHand								
DominantHandFirstAttempt								
DominantHandSecondAttempt								
DominantHandThirdAttempt								
NonDominantHandFirstAttempt								
NonDominantHandSecondAttempt								
NonDominantHandThirdAttempt								
CALC_DominantMeanGripStrength								
CALC_NonDominantMeanGripStrength								
CALC_FriedTotalScore								

Enquiries to lesley.brown@bthft.nhs.uk
Project number: [office use only]

CARE75+ DATA REQUEST FORM

This form is to be used for all data request purposes including; sampling, preparatory work and research.

The request contact will be responsible for the transfer, storage and governance of the data in line with the data sharing agreement (appendix 1).

All sections *must* be completed.

1. Office use only

Data request number	
Date of request	
Date of review by DRRC	
Date of DRRC query	
Date of query review	
Data sent	

For peer review only

---

2. Request contact

Study Name:	
Requested by:	
Contact:	
Email	
Telephone	
Job role	
Organisation	
Date of request	
Date required	

For peer review only - http://bmjopen.bmj.com/site/about/guidelines.xhtml

Enquiries to lesley.brown@bthft.nhs.uk
Project number: [office use only]

3. Data Handling. If anyone else is expected to handle (i.e. view/analyse/transfer/store) these data in association with the study named in section 1, please list them here:

Name	Affiliation	Title	Role in the project

4. Purpose of request

Sampling i.e. participant contacts	
Scoping exercise e.g feasibility/protocol development	
Research e.g. analysis for funded/approved projects	

5. Research question and brief summary of research (350 words)

6. What type of data do you require?

Individual identifiable data (contains personal details)	
Pseudo-anonymised (contains unique id for data linkage)	
Anonymised (contains no identifiable details)	

For peer review only - http://bmjopen.bmj.com/site/about/guidelines.xhtml

Enquiries to lesley.brown@bthft.nhs.uk
Project number: [office use only]

7. What stage do you require (NB: full data will not be available for all participants)

Baseline	
Six month follow-up	
12 month follow-up	
24 month follow-up	
48 month follow-up	
Latest time-point	

8. Selection criteria

Included if:	
But excluded if:	

9. Specific data items required

Date of Assessment required?		
**Data dictionary sheet title**	**Variable name** (please cut and paste from data dictionary)	
Contact information		
Personal details		
Housing, Living Circumstance		
Education, Occupation		
Family Data		
Formal and Informal Support		
Smoking, Alcohol		
Hearing, Eyesight		
SF-36		

For peer review only - http://bmjopen.bmj.com/site/about/guidelines.xhtml

Enquiries to lesley.brown@bthft.nhs.uk
Project number: [office use only]

MoCA	
Co-morbidities	
Falls	
Medications	
Activities of Daily Living	
Height, Weight, BP, Grip	
Timed Up and Go, Walking	
Frailty	
Quality of Life and Sleep	
Pain	
Loneliness, Depression	
Resilience, Self-Efficacy	

10. Please can you provide details of your intended output (for example, publication or report)

For peer review only
